# 1,2,3-Triazole-linked DNA biosensors: molecular design to real-time applications

**DOI:** 10.1039/d6ra06492b

**Published:** 2026-09-22

**Authors:** Gaurav Bhatoe, Nancy George, Gurjaspreet Singh, Anshu, Jandeep Singh

**Affiliations:** a School of Chemical Engineering and Physical Sciences, Lovely Professional University Phagwara-144411 Punjab India singhjandeep@gmail.com; b Department of Chemistry and Centre of Advanced Studies in Chemistry, Panjab University Chandigarh-160014 India

## Abstract

Many aspects of modern life rely on rapid, accurate, and selective detection methodologies, especially for heavy toxins, pathogens, biomolecules, metal ions, and miRNAs. Thus, there is a need for reliable and versatile advanced biosensing platforms that can meet these challenges. CuAAC-assisted systems have emerged as efficient tools for constructing DNA biosensors due to their high efficiency, bioorthogonality, and site-specific conjugation capabilities. This review highlights the role of copper(i)-catalyzed azide–alkyne cycloaddition (CuAAC) in DNA biosensing platforms. Emphasis is placed on the use of CuAAC-aided 1,2,3-triazoles for different linkages, surface modifications and dual-strand linkages. Furthermore, the integration of click chemistry with nanomaterials, DNA-based structures, DNAzymes, CRISPR, and ATRP for signal amplification and highly sensitive detection is discussed. Various signal transduction mechanisms are included, such as fluorescence, electroluminescence, and electrochemical signal outputs. In addition, the review evaluates recent discoveries, underlying mechanisms and current limitations, enabling a more in-depth understanding of how click chemistry is reforming DNA-based biosensing. Overall, this work underscores the power of 1,2,3-triazole to enhance next-generation biosensors with improved sensitivity, selectivity, and real-world applicability.

## Abstract 2

This review highlights CuAAC in DNA biosensing, using 1,2,3-triazole for linkage, surface and dual-strand modification, integrated with nanomaterials, DNA structures, DNAzymes, CRISPR and ATRP for signal amplification and precise detection.

## Graphical abstract

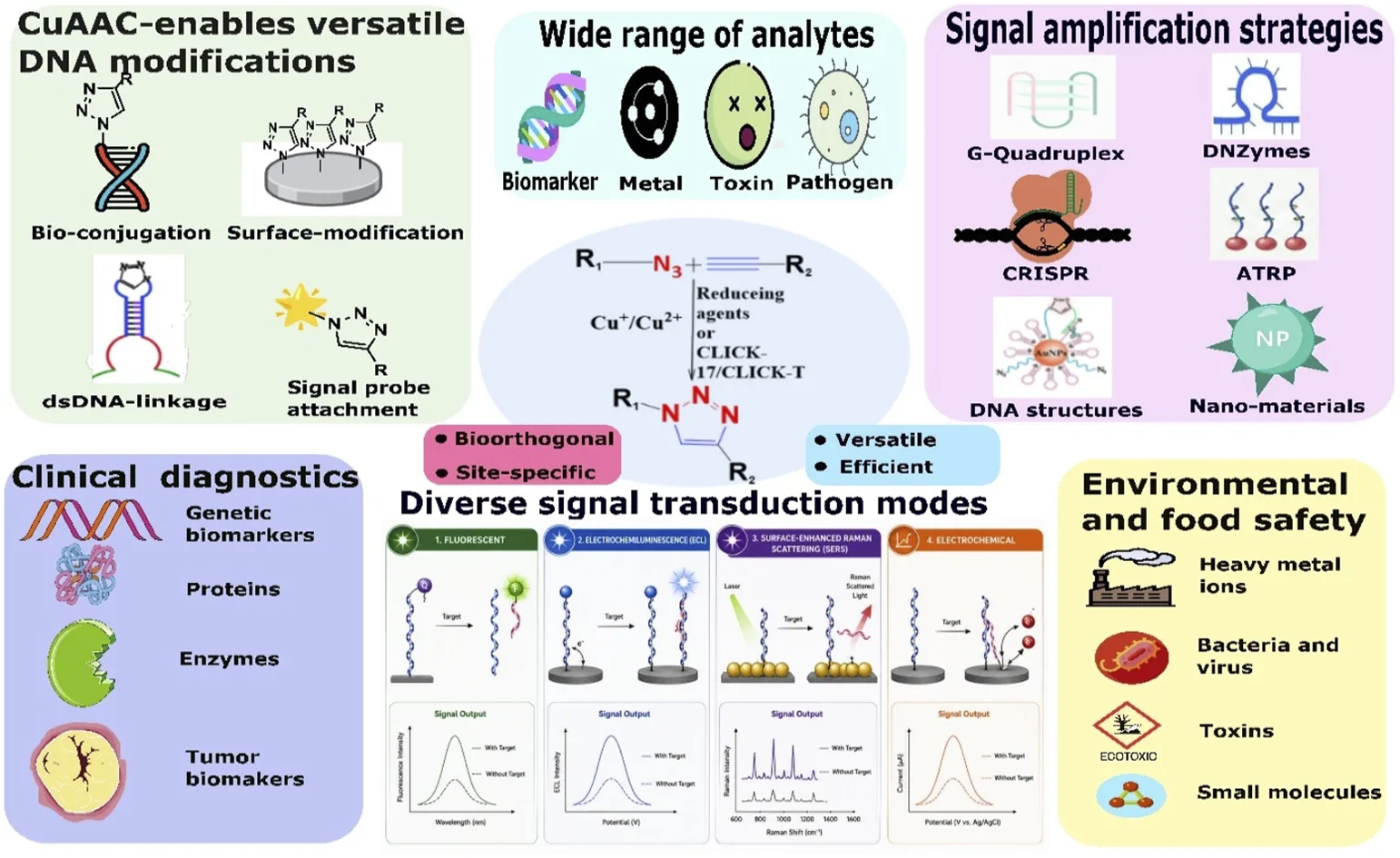

## Introduction

The need for rapid, selective, and on-site identification of many targets to achieve a disease-free and hazardous-material-free environment for humans has been a major goal for scientists across generations.^1,2^ It has led to the discovery of numerous conventional methods for the early and facile detection of diseases and toxins, such as the use of polymerase chain reaction (PCR), enzyme-linked immunosorbent assay (ELISA), high-performance liquid chromatography (HPLC), gas chromatography, and mass spectrometry. However, these techniques are not always available to developing or under-developing countries and distant locales owing to the high instrumentation cost and the requirement for skilled operators.^3–5^ To address this, biosensing has become a popular analytical approach for detecting targets in food, the environment and living systems.^6^ Biosensors typically integrate a biological recognition element with a transducer that converts a biochemical activity into a measurable signal (Fig. 1).^7,8^ Owing to their rapid, sensitive, low-cost, point-of-care, and real-time detection capabilities, biosensors have attracted significant attention in the last decade as the field of analytical science has grown.^9–11^

**Fig. 1:**
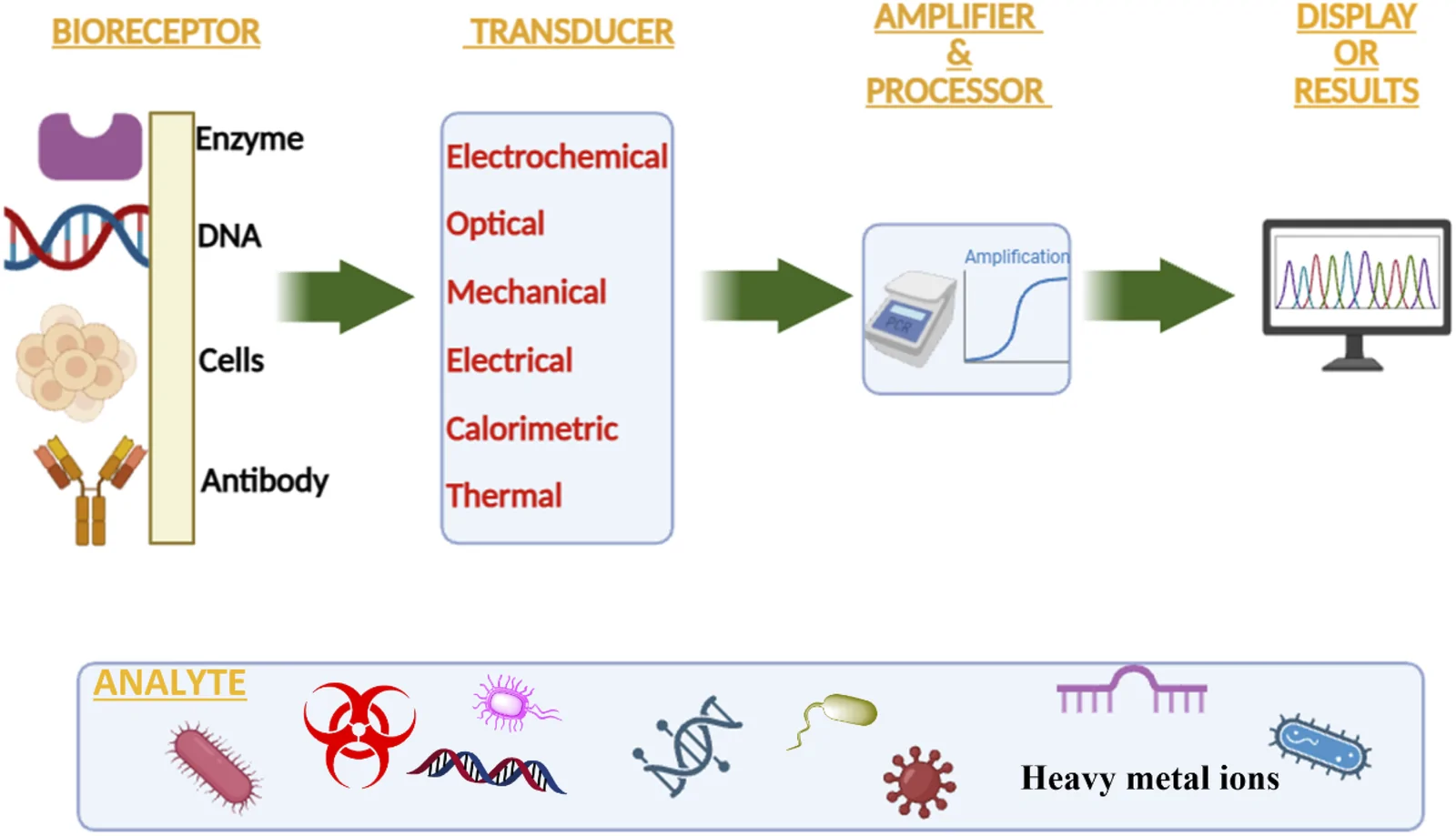
Schematic of the general principle of biosensing, with analyte detection by a bioreceptor, signal transduction, and the formation of a detectable analytical response.

Among the various types of biosensors, DNA (deoxyribonucleic acid) biosensors have gained prominence due to the exceptional molecular recognition ability of DNA.^12^ The base pairing rules bind each DNA strand to its complementary sequence, similar to a lock-and-key mechanism, making it unique; this allows scientists to tune and design DNA hybridization processes for selective target detection.^12–14^ Furthermore, the structural flexibility of DNA, including its ability to form double strands, hairpins, and G-quadruplexes, as well as its ability to attach to other molecules, aids in biosensing.^13–16^ The selective binding of DNA to target strands enables the precise detection of pathogens, genetic mutations, and toxins.^12^

Despite these unique advantages, conventional DNA biosensors are limited due to the poor immobilization of DNA probes to the sensor surface and the attachment of signal probes to the sensor resulting in poor stability, random orientation and weak linkages of probes to sensors.^17–19^ The simple colourimetric, fluorescence, and electrochemical signal outputs pose challenges for DNA biosensors due to poor signal generation and probe linkages, which restrict efficiency and reproducibility.^20,21^ Conventional DNA biosensors are thus often insufficient for real-life and complex sample analysis, achieving poor sensitivity and selectivity.^22^

To address these challenges, click chemistry has emerged as a solution for DNA biosensors, owing to its robust, efficient, and site-specific conjugation.^23,24^ In 2001, the high-yielding, fast and versatile reactions were introduced by Sharpless and co-workers, and after their discovery, they became highly efficient and widely used due to their reliability, regioselectivity, and biocompatibility.^23,25^ The biocompatibility of click chemistry made it useful for biological and chemical fields. More specifically, the copper(i)-catalyzed azide–alkyne cycloaddition (CuAAC) reaction product, 1,2,3-triazole, allows robust and bio-orthogonal attachment for DNA probes, signal tags, and recognition elements on sensor surfaces, which not only provides stability but also improves electron transfer and signal transduction efficiency.^26–28^

In the past decade, although advanced research has demonstrated the use of click chemistry in DNA biosensor fabrication, comprehensive, well-defined explanations of 1,2,3-triazole in DNA biosensors from different mechanistic perspectives remain limited. These biosensors have shown promising results for real-world complex sample analysis, with impressive recovery rates yet to be fully realised. Recent developments in the CLICK-17 DNAzyme-catalysed click reaction remain underexplored. Moreover, click-aided polymerisation for signal probes, 1,2,3-triazole-based probes and surface linkages, and DNA quadruplex formation are still underexplored. This review aims to summarise the contributions of 1,2,3-triazole in DNA biosensors for different signal amplification strategies and signal transduction methods. The application of click-assisted DNA biosensors in the medical and environmental fields covers a major part of this review. The review focuses on 1,2,3-triazole DNA biosensors for target detection in real-time samples such as biological fluids and environmental matrices, where these click-assisted sensors reduce false positives or copper sequestration in uncontrolled samples by the use of reducing agents that maintain the Cu(i)/Cu(ii) equilibrium. Prospects are outlined to guide the next generation of scientists in the discovery and development of DNA biosensors based on click chemistry.

### Roles of 1,2,3-triazole in DNA biosensors

Click chemistry has emerged as an exceptionally fruitful tool across multiple disciplines, owing to its ideal behavior in biological and analytical applications, its inertness and thermodynamic stability, facile surface modification, bioconjugation, and resistance to enzymatic degradation.^29–32^ Unlike other coupling methods, 1,2,3-triazole synthesis requires mild conditions and proceeds with high efficiency and regioselectivity, enabling its use to functionalize DNA strands and sensing interfaces.^33–35^ Subsequently, 1,2,3-triazole-based conjugations have been integrated with DNA systems to enhance biosensors with respect to signal transduction, sensitivity, selectivity and other amplification strategies. Given these characteristics, 1,2,3-triazole has become a crucial tool for the development of sophisticated DNA biosensing platforms.^36–39^ In this section, 1,2,3-triazole-based modifications reported for DNA-based biosensors are discussed, including CuAAC for surface modification and probe attachments.

#### 1,2,3-Triazole-based surface linkage

Given that 1,2,3-triazole is a robust molecular linker, CuAAC has been used widely for the fabrication and modulation of DNA biosensors. In DNA-based biosensors, the conjugation of DNA to the surface of a carrier or to an electrode for signal amplification requires an efficient and stable approach.^40^ The most common and highly preferred linkage methods involve thiol–gold (Au–S) and amine-carboxyl linkages, and the CuAAC reaction approach. However, while Au–S is limited to gold electrodes, and amine-carboxyl is useful in carbon electrodes only,^41,42^ click conjugation provides a versatile linkage service by combining two nanoparticles to merge into one aggregated structure (Fig. 2).^43,44^

**Fig. 2:**
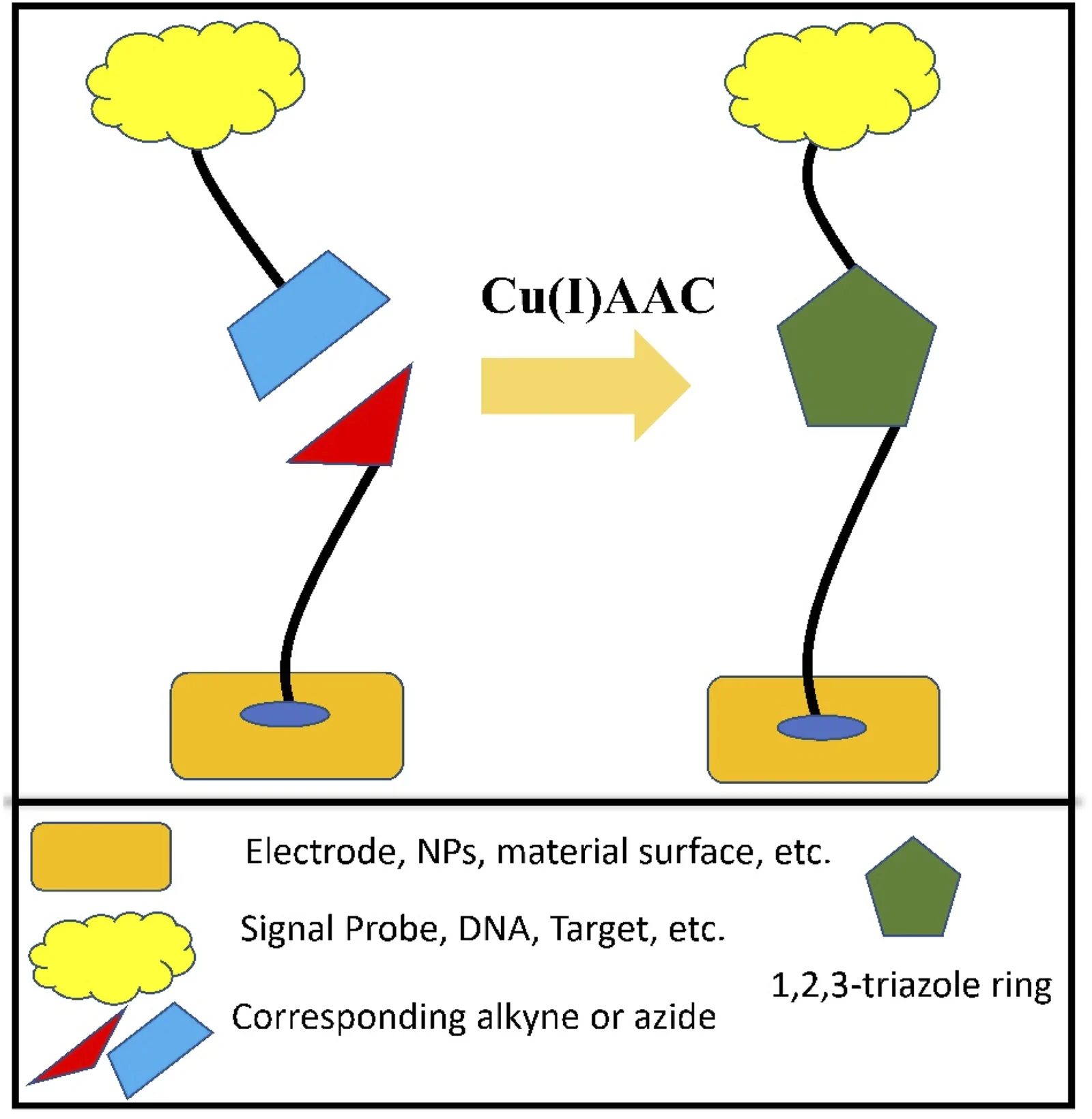
Principle of linkage through 1,2,3-triazole for biosensors.

Chen *et al.* synthesised a microparticle-counting immunosensor based on PDA nanoparticle-mediated click chemistry for the sensitive detection of ochratoxin A (OTA) with a detection range of 0.5–800 ng mL^−1^ and an LOD of 0.2 ng mL^−1^. The polydopamine nanoparticles conjugated with secondary antibody (Ab_2_) served as an immune carrier that had a strong ability to chelate with copper(ii) ions. The PDA-Ab_2_ conjugated with ochratoxin A in a 96-well plate, with its concentration inversely controlled by the availability of copper(ii) ions. At the same time, some copper(ii) ions were reduced to copper(i) by l-ascorbate sodium salt, which further catalyses the click reaction to fuse alkyne- and azide-functionalized polystyrene (PS) microspheres to form aggregated PS clusters. The numbers of aggregated PS clusters and dispersed PS clusters were recorded through particle counting technology. The approach was used for the analysis of barley and wheat samples, with recovery rates of 91.5–103.4%, and high precision and accuracy. Although the sensor could not detect multiple targets, it was sufficiently accurate for the detection of trace targets of ochratoxin A in food and environmental samples (Fig. 3).^45^

**Fig. 3:**
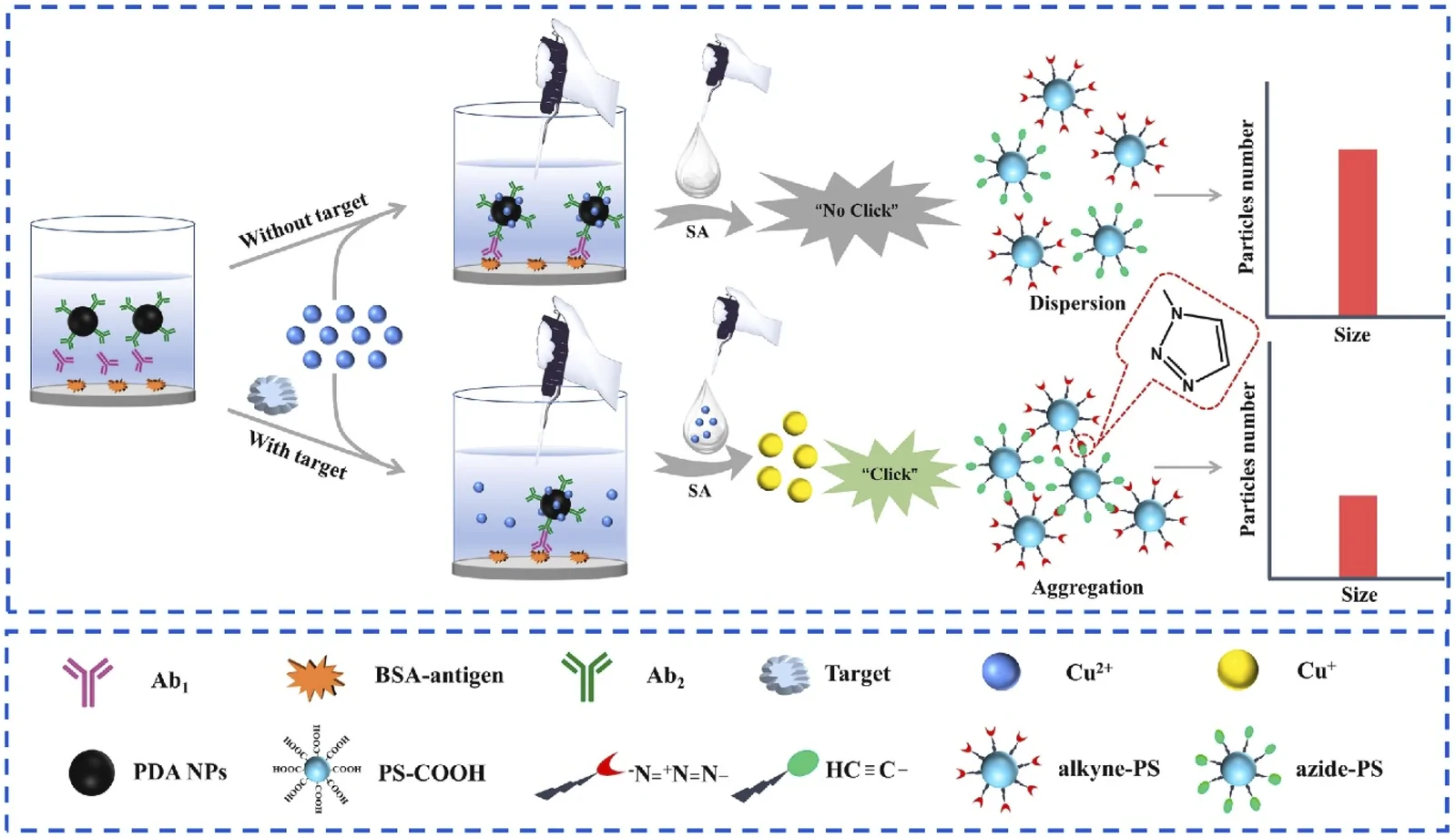
Schematic of a microparticle-counting immunosensor employing polydopamine nanoparticle-facilitated click chemistry between azide/alkyne functionalized polystyrene microspheres^45^ (adapted/reproduced from ref. 45 with permission from Elsevier, copyright 2022).

Scientists utilized click conjugation for surface linkages; however, covalent linkages such as Au–S are also used for attachment of azide or alkyne groups to surfaces.^42^

Lei *et al.* synthesised an electrochemiluminescent (ECL) nanosensor based on CoFe_2_O_4_ magnetic nanoparticles (MNPs) modified with gold nanoparticles functionalized by azido-terminated thiolated strands (DNA1). These strands were linked to DNA2 (terminal alkynyl) *via* click chemistry, forming the 1,2,3-triazole derivative ring in the presence of copper(ii) ions. The initiator strands of DNA1 were designed to trigger a hybridization chain reaction (HCR), resulting in the formation of a long, nicked dsDNA structure. An amino-terminated perylene derivative was introduced that facilitated electrostatic adsorption, intermolecular hydrophobic interactions and π–π stacking. This biosensor exhibited ultra-sensitivity toward copper(ii) ions, which were successfully detected in real biological samples, including human hair solution. By employing click chemistry in quasi-homogeneous environments, these ECL nanosensors demonstrated great sensitivity, superior selectivity, and good reproducibility, underscoring their potential as a practical platform for reliable detection of other clinically relevant analytes.^46^

In another independent study, Lupoi *et al.* engineered an electrochemical biosensor for diclofenac detection with an LOD of 17.95 µM. In this biosensor, aryl diazonium salts with different protecting groups were covalently functionalized onto the GCE and subsequently underwent click conjugation with an azide-bearing aptamer. Consequently, 1,2,3-triazole conjugation resulted in surface modification for linkage to the recognition probe. Further, target diclofenac binds to the aptamer and results in an electrochemical redox change. This biosensor was also used to study aptamer spacing distribution over the electrode with different protecting groups and their impact on biosensing.^47^ Similarly, Lupoi and co-authors fabricated another biosensor based on a graphene field-effect transistor as an electrode surface for diclofenac detection. The biosensor used a similar click-assisted mechanism with similar working phenomena and signal amplification in electronic response, with an LOD of 3.1 × 10^−3^ µM (Fig. 4).^48^

**Fig. 4:**
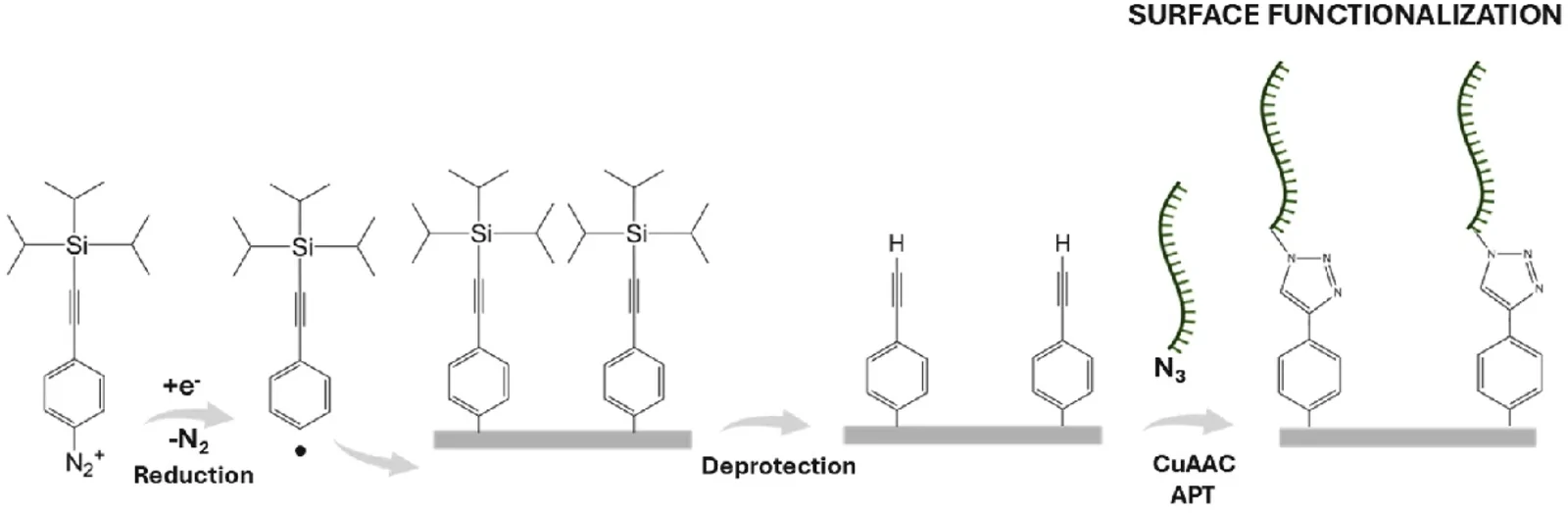
Schematic of the sensor GFET modified through the electroreduction of an ethynylaryl diazonium salt, protected by TIPS, followed by a chemical deprotection process and azide-modified aptamer immobilization *via* copper-catalyzed click chemistry^48^ (adapted/reproduced from ref. 48 with permission from Elsevier (an open access paper used under the terms of the Creative Commons Attribution (CC BY) license), copyright 2026).

The use of 1,2,3-triazole as a linker is driven by its aromatic stability, resistance to metabolic degradation and applicability to any system, and it is not limited to surface linkages.^49^ Qiu *et al.* developed a sensor for OTA based on click chemistry-assisted DNA in a system that involved the formation of a double-stranded DNA (dsDNA) structure through stitching of an azido-OTA aptamer-biotin (DNA1) functionalized magnetic bead (MB) with an alkyne-modified DNA-invertase conjugate (DNA2). However, due to the instability of the dsDNA, it led to partial release of the DNA2 strand in the supernatant during magnetic separation, generating a high background signal. This issue was resolved using a click chemistry reaction between DNA1 and DNA2, resulting in 1,2,3-triazole linkages that stabilized the DNA duplex on the surface of the magnetic beads (MBs), significantly reducing the background signal by nearly eightfold. In the presence of OTA, the aptamer (DNA1) is preferentially bound to OTA, thereby preventing hybridization and resulting in the release of DNA2 strands into the supernatant. The biosensor was applied to feed sample analysis and demonstrated recovery rates ranging from 86% to 103.5%. With a detection limit of 72 pg mL^−1^, this biosensor shows strong potential for point-of-care testing and food safety applications (Fig. 5).^50^

**Fig. 5:**
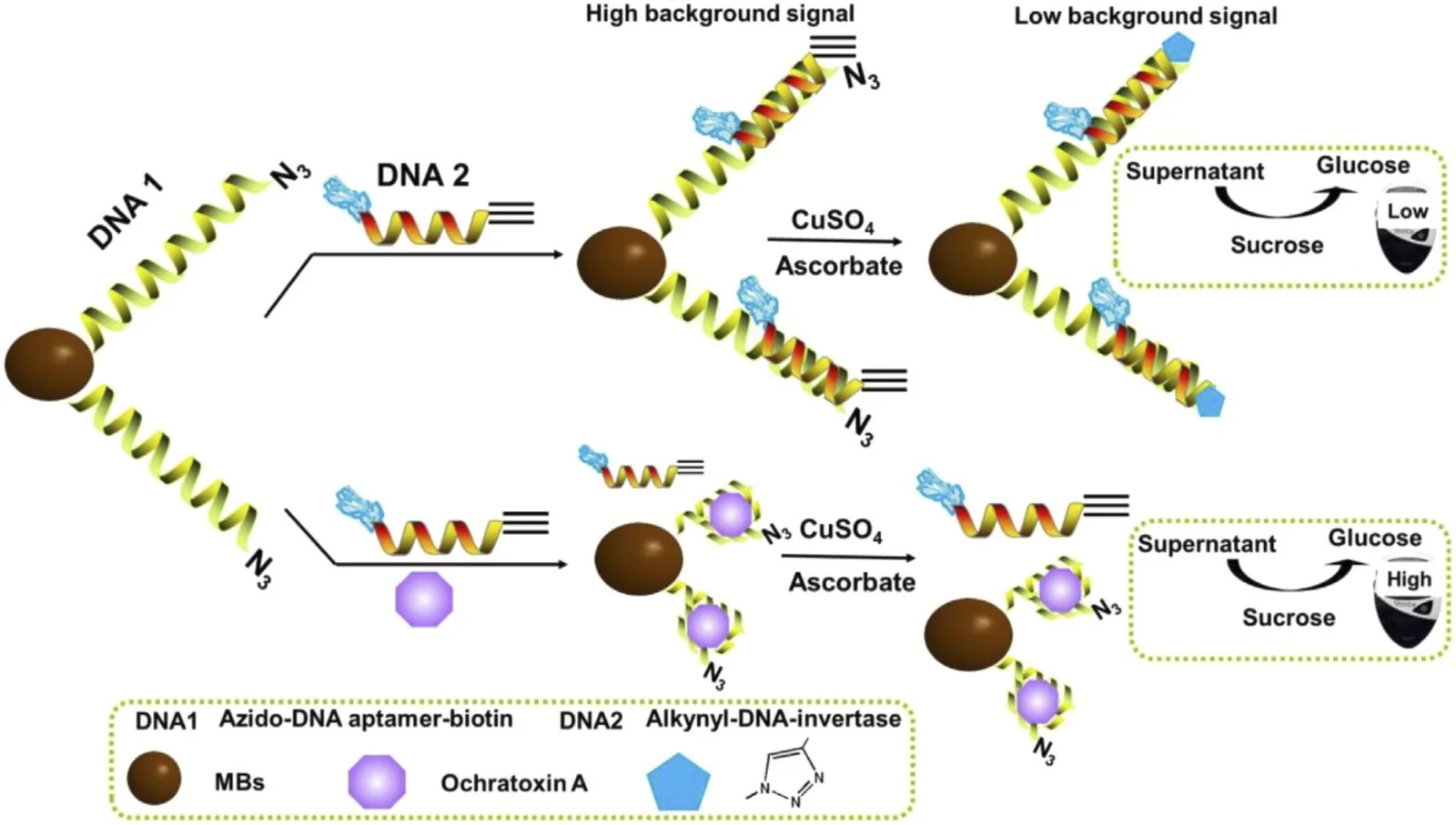
1,2,3-Triazole click ligation with an azido-OTA aptamer-biotin (DNA1) and alkylnyl-DNA-invertase strand (DNA2)^50^ (adapted/reproduced from ref. 50 with permission from Elsevier, copyright 2019).

Cao and co-workers fabricated a biosensor for subpopulations in circulating extracellular vesicles (EVs) analysis in serum samples. The biosensor involved target-functionalized magnetic beads and used anti-CD44 antibody for recognition of CD44-positive EVs. Further, an antibody-aided HRP reaction catalysed the oxidation of tyramine alkyne in the presence of hydrogen peroxide, resulting in the formation of reactive species that covalently linked the alkyne groups to the near CD44-positive EV surface. Subsequently, azide-conjugated electroactive AuNPs reacted through click conjugation with surface-localised alkyne groups to form triazole linkages that amplify the electrochemical signals. Thus, click chemistry facilitates the binding of electroactive AgNP signal probes, establishing CuAAC as a key element in the strategy for signal amplification.^51^

Liu and group developed a biosensor for the identification of 5 mC DNA in serum samples with spiked recovery rates of 95.60% to 103.94%. The biosensor utilized a tetrahedral DNA nanostructure functionalized with a gold-coated anodic aluminium oxide nanochannel. In the presence of the target 5 mC, HpaII failed to cleave the specific target sequence, which resulted in an intact methylated strand to conjugate with the DNA nanostructure, which initiated the HCR to generate long DNA assemblies with many alkyne groups. This was followed by click conjugation between azide-conjugated AuNPs and alkyne moieties of the DNA nanostructure to generate the 1,2,3-triazole functionality for signal probe modification. Unmethylated DNA units in the DNA nanostructure were cleaved by HpaII, which restricted the HCR for alkyne functionalization and eventually failed the click conjugation.^52^

#### Dual-end-modified DNA strands *via* 1,2,3-triazole formation

DNA naturally possesses a free phosphate group at the 5′-carbon and a free hydroxyl group at the 3′-carbon of the sugar–phosphate backbone, which are essential for enzymatic activity and DNA replication.^53^ However, the behaviour and functionality of DNA can be deliberately altered through artificial modifications at one or both of its 5′- and 3′-termini. Such modifications may be chemical, structural, or achieved through conjugation with specific functional molecules to obtain desired properties.^54^ In click chemistry-based approaches, DNA strands are typically modified with a 5′-alkyne group on one strand and a 3′-azide group on the other.^55,56^ These terminally functionalized DNA strands undergo CuAAC to form dual-end-linked double-stranded DNA containing a stable 1,2,3-triazole linkage.^57^ The resulting end-linked dsDNA structures were successfully employed in biosensor engineering and have demonstrated excellent detection performance.

Huang *et al.* fabricated a biosensor that exhibited dual-mode signal fluorescence and colorimetric copper(ii) ion detection in human blood samples. The detection process relies on a synthesised double-stranded DNA probe with azide and alkyne moieties immobilized on the two opposite sites. Upon introduction of copper(ii) ions, the dsDNA probe underwent intramolecular cyclization *via* a click reaction, resulting in the formation of a hairpin structure *via* 1,2,3-triazole linkage. The generated hairpin structure underwent a primer exchange reaction (PER), forming products containing G-triplex sequences. G-triplex was bonded to thioflavin T (ThT) for a fluorescent response, whereas G-triplex bonded to hemin to form a peroxidase-mimicking enzyme to oxidise colourless ABTS to green ABTS^−^ in the presence of H_2_O_2_, for a calorimetric response. The biosensor demonstrated recovery rates of 91.7% to 107.1% as well as an LOD of 6.85 × 10^−2^ and 7.04 × 10^−2^ µM in fluorescence mode and colorimetric mode, respectively. The sensor has great potential for applications in public health, medical research, and environmental monitoring. The 1,2,3-triazole held the DNAzyme and stabilized it in the cleavage process, forming a quadruplex for detection *via* calorimeter signals through oxidation of ABTS.^58^

Zhang and co-authors engineered a fluorescence polarization sensor based on CuAAC and implemented it for the detection of ampicillin. The availability of copper(ii) ions enabled the detection of ampicillin in the absence of an ampicillin-specific aptamer. Further, Apt/P/C-FAM hybrid complexes were formed in the presence of C-FAM (carboxyfluorescein) and exonuclease 1 (EXO-1), which led to the release of fluorophores that were absorbed by 2-D nanosheets of GO (graphene oxide) and acted as fluorescence polarization signal enhancers. However, ampicillin-bound DNA aptamer hindered the click reaction, which represented the first instance of using click chemistry to develop an enhanced fluorescence polarization sensor for antibiotic detection. Click-reaction-modified dsDNA became stable due to 1,2,3-triazole bridging, and this straightforward linking reaction yielded a stronger signal, ensuring the reliability of the analysis. This sensor exhibited a low LOD of 80 pg mL^−1^ and strong analytical performance in milk and river water samples, with recovery rates of 95% and 107%, respectively. These results suggested that the sensor has great promise for applications in environmental monitoring and food safety (Fig. 6).^59^

**Fig. 6:**
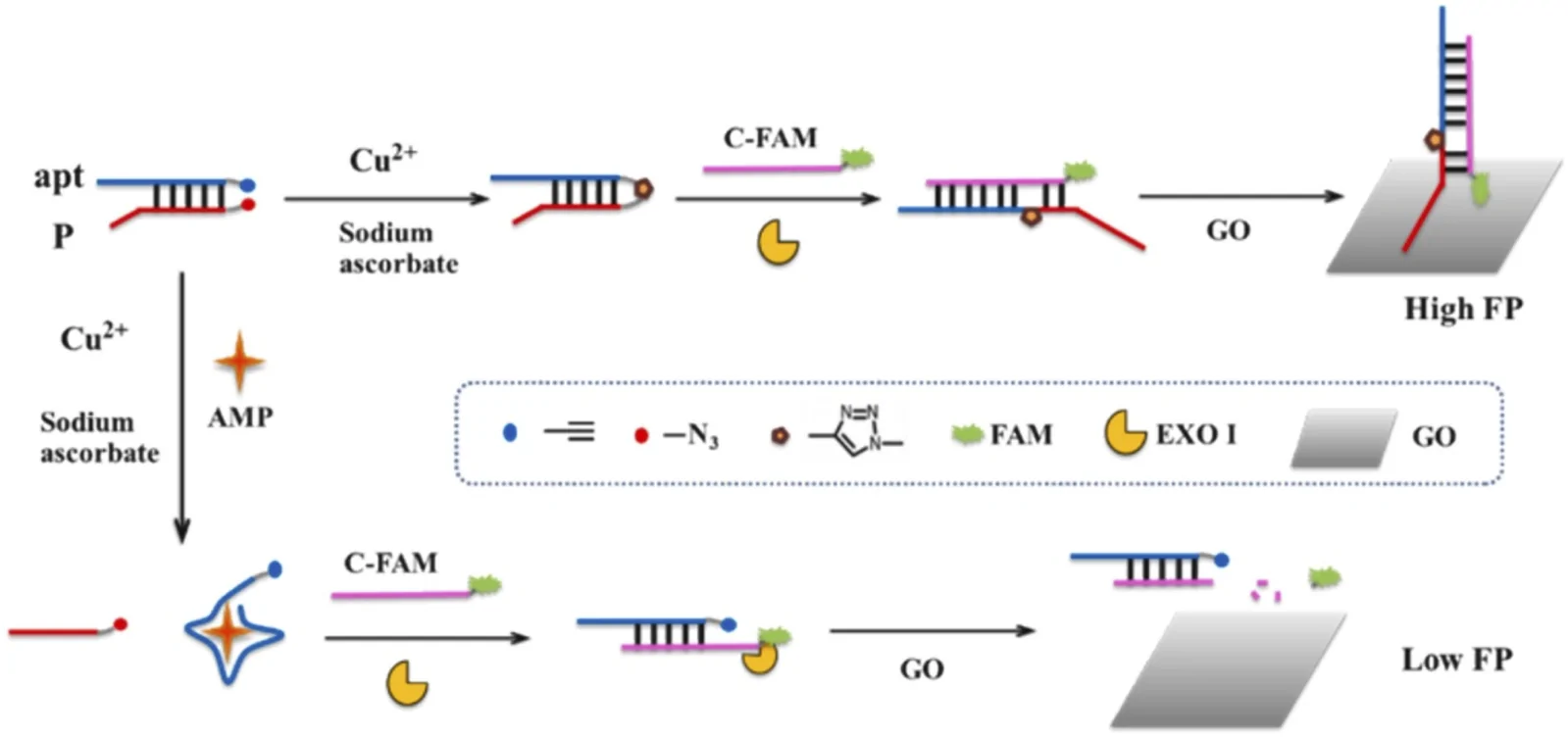
Schematic of the amplified FP sensor for ampicillin using click chemistry and graphene oxide^59^ (adapted/reproduced from ref. 59 with permission from Elsevier, copyright 2024).

Zhang and co-authors designed an electrochemical biosensing technique using a multidriven strand displacement reaction and click chemistry for miRNA-21 detection. The sensor mechanism involves SDR with five DNA strands attached to a glassy carbon electrode (GCE), an azide-immobilized strand M with a toehold, along with a probe attached to electroactive ferrocene with three strands with an alkyne moiety. With the addition of miRNA-21 to the DNA system, released output signals invaded the duplex system *via* the toehold. 1,2,3-Triazole formed by CuAAC functionalized M strand, and alkyne functionalized R strand, and stabilised the invasion associated with the release of ferrocene.^60^

Daxiu Li and co-workers developed a copper(ii) ion detection platform in human blood serum relying on metal ion-dependent DNAzymes. The 1,2,3-triazole formed a complete Mg^2+^-dependent DNAzyme that reconstituted the DNAzyme, ready to bind the substrate hairpin DNA for catalytic cleavage. In the presence of Mg^2+^, the active DNAzyme breaks the substrate hairpin DNA and releases G-rich sequences, which then engage with hemin, resulting in peroxidase-like G-quadruplex/hemin complexes that enhanced the oxidation of ABTS^2−^, producing the green ABTS^−^ radical and generating a visual signal. The cyclic cleavage mechanism enhances the signal, allowing for sensitive and selective colorimetric detection of copper(ii) ions, with a biosensor exhibiting an ROD 5 × 10^−3^ to 0.5 µM and a detection limit of 2 × 10^−3^ µM, demonstrating its potential for real-life samples.^61^

#### Click-assisted polymerization for signal probe modification

Zhu *et al.* synthesised an electrochemical biosensor for exosome detection in human serum samples based on click polymerization. The biosensor mechanism involved an anti-EFGR functionalized gold electrode, which captured the exosomes of lung cancer cell A549. Subsequently, sodium alginate grafted glycidyl propargyl ether linked to exosomes through a PO_4_^3−^–Zr^4+^–COOH coordination bond, and an electroactive polymer P (DES-BFPA) synthesised through click chemistry was immobilised for signal amplification.^62^

Cheng and co-authors worked on an electrochemical biosensor for the detection of human T-lymphotropic virus type II by employing a click polymerisation-based electro-polymer signal probe. The working biosensor was initiated by polymer synthesis *via* click triazole linkage between 1,4-diacetylenebenzene (DEB) and 4,40-diazido-2,20-stilbenedisulfonic acid disodium salt tetrahydrate (DSDA). Subsequently, the gold electrode-functionalized hairpin DNA probe underwent hybridisation with an HTLV-II probe conjugated with an azido moiety. Further, a click reaction occurred between the azide group of the HTLV probe and the alkyne group of the P(DEB-DSDA), which resulted in an electrochemical signal that was monitored *via* electrochemical impedance spectroscopy.^63^

Liu and co-workers constructed a signal-off aptasensor based on click polymerization and complementary DNA for Aflatoxin B_1_ (AFB_1_) detection in two food samples (malt and corn) and six herbal samples, with detection rates ranging from 94.73–101.46%. The analytical approach involved synthesizing the sensor from propargylamine and 1,1′-ferrocenedicarboxylic acid to yield bis-ferrocenoyl propargylamide, which further underwent a click reaction to give 2′-stilbene disulfonic acid disodium salt tetrahydrate (DES), which acts as an active click polymer P(BFPA-DES) linked *via* 1,2,3-triazole. The gold electrode was then modified with the thiolated aptamer and c-DNA bearing an azide group *via* Au–S bond formation. The alkyne moiety of the P(BFPA-DES) signal probe conjugated to the azide-DNA-functionalized electrode through the click reaction. AFB_1_ was introduced to react with the c-DNA and the aptamer. Subsequently, c-DNA was discarded, reducing the current peak. Click polymerization enhanced the signal amplification and raised the ultra-sensitivity of the sensor, with the range of detection varying between 2 fg mL^−1^ and 2 ng mL^−1^ and an LOD of 0.804 fg mL^−1^, proving its suitability for analyzing food and drugs.^64^

Wen *et al.* assembled a fluorescence sensor involving DNA-based click polymerization for IFN-γ detection with a LOD of 1.63 fM in fetal bovine serum. This biosensor technique involved conjugation of Fe_3_O_4_-aptamer 1 to IFN-γ, which was further attached to azide-modified aptamer 2, resulting in a sandwich structure. Subsequently, FAM-DNA 1 (alkynyl and carboxyfluorescein modified) and azide-modified aptamer 2 were conjugated to form dsDNA, with a sandwich structure linked by forming a 1,2,3-triazole ring *via* CuAAC reaction, which resulted in click polymerization. The 1,2,3-triazole linker not only acts as a tool for polymerization but also enhances the fluorescent signal, with higher sensitivity and selectivity, making it optimal for clinical ultrasensitive protein detection, with an ROD of 0.01 pM–10 nM and an LOD of 1.75 fM (Fig. 7).^65^

**Fig. 7:**
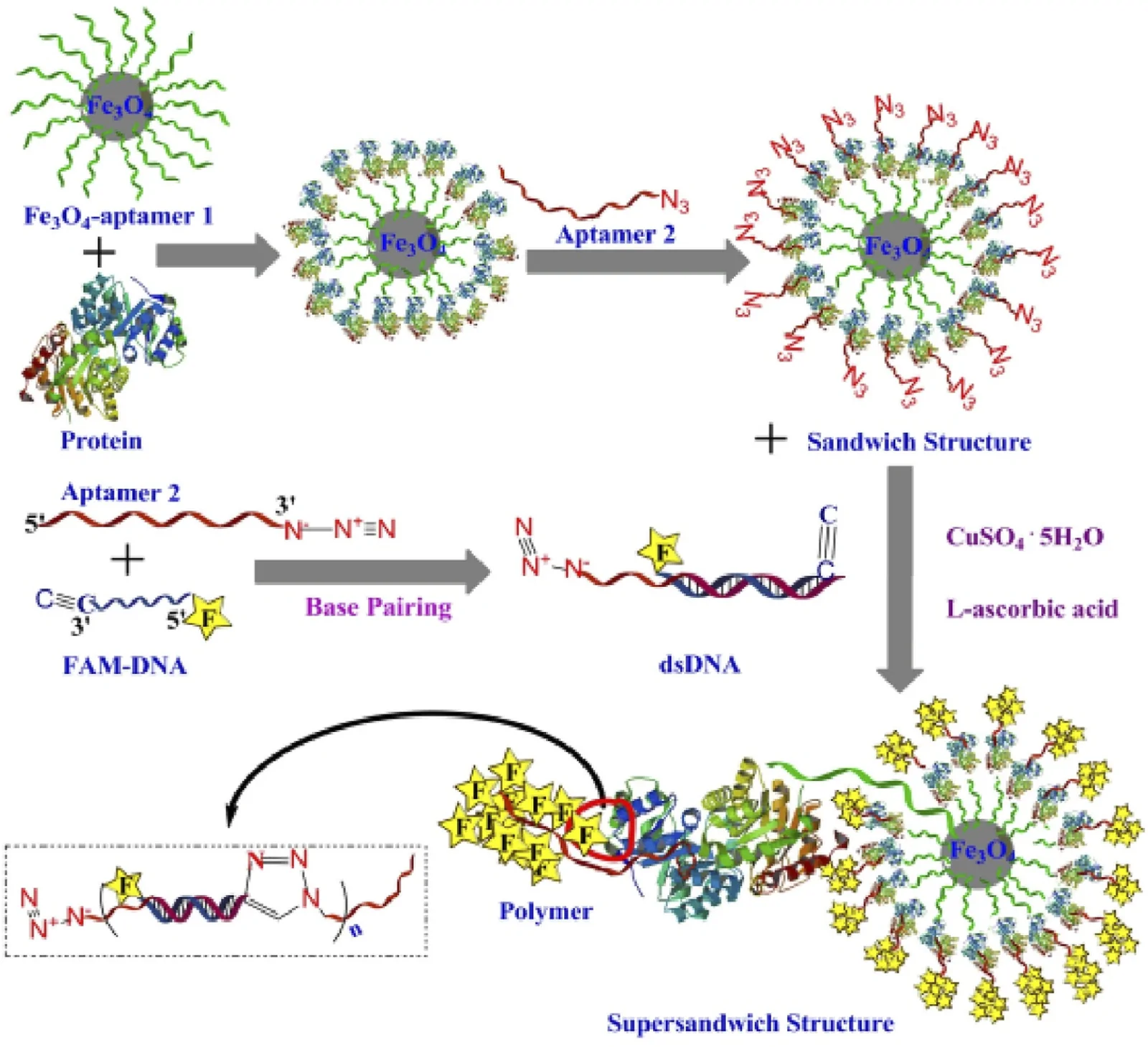
DNA-based click polymerization used to create a super-sandwich fluorescence sensor for detecting IFN-γ with high sensitivity^65^ (adapted/reproduced from ref. 65 with permission from Elsevier, copyright 2022).

Hence, the above-reported studies employed the click reaction as an excellent approach for DNA biosensor mechanisms; despite being used as a linkage tool, it also has the potential to improve the stability and performance of the overall biosensor. The repeated intermolecular coupling between multi-functionalized monomers with end moieties of alkyne/azide results in real step-growth click polymerization with chain growth; however, differentiation of the product can be done using molecular weight analysis such as (Mn), (*M*_w_), and dispersity analysis with gel permeation chromatography (GPC) and size exclusion chromatography (SEC). It is a better approach for modern sciences as it applies not only to surfaces but also to other systems, such as signal and DNA probes.

### Signal transduction strategies

Signal transduction is a major component of biosensors, which converts the target recognition into a measurable signal output. A good signal transduction method affects the overall performance by influencing sensitivity, selectivity, response time, and LOD of the biosensor.^66,67^ 1,2,3-Triazole-based DNA biosensors have efficient DNA probes and site-specific conjugation provided by CuAAC, which enhances the signal transduction of the biosensor. 1,2,3-Triazole provides conjugation for different signalling elements, such as fluorophores, redox-active molecules, luminophores, and nanomaterials.^68–70^ Subsequently, 1,2,3-triazole-assisted conjugation provides molecular interactions for signal transduction to form different signals, such as optical, electrochemical, or spectroscopic outputs.^66,68,71^ Among these, fluorescence, electrochemical, electrochemiluminescence and surface-enhanced Raman scattering strategies are widely used.

#### Fluorescence-based signal transduction

Owing to its rapid response time and high sensitivity, fluorescence is widely used in signal transduction. In fluorescence-based biosensors, target recognition alters the fluorescence intensity, wavelength and energy transfer efficiency.^72,73^ With the aid of 1,2,3-triazole, site-specific attachment of fluorophores and quenchers to biosensors can be achieved without altering the biological activity. Recently, a 1,2,3-triazole-courmarin derivative has been used for fluorescence-signal-based detection of target analytes.^74,75^

Wang and co-workers constructed a dual signalling biosensor based on a CLICK-17 DNAzyme-catalysed click reaction for *V. parahaemolyticus* detection, in line with the approach by Hang Zhou *et al.* The Biosensor involved a gold nanobipyramid, an antimicrobial peptide and ferrocene (Fc)-labelled DNAzyme immobilised on a MXene surface. When the target is present, the antibody, VP, and MAADF created a sandwich complex on the surface of the electrode. The DNAzyme-ferrocene acted as a catalyst for the click reaction between AHC and BOL, generating a coumarin-derived 1,2,3-triazole ring that exhibited fluorescence signals, whereas the dissociated ferrocene provided electrochemical signals. The biosensor exhibited recovery rates of between 94.0% and 106% in shrimp, crab, and fish samples, along with an LOD of 6 CFU mL^−1^ and an ROD of 10–10^8^ CFU mL^−1^.^76,77^ The studies mentioned above emphasize the effectiveness of fluorescent coumarin-based 1,2,3-triazole systems in biosensing applications. Utilizing click chemistry for the creation of signal probes streamlines the design process while improving the biosensor's sensitivity and stability.

The Zhou group engineered a fluorescent biosensing platform for the detection of *Pseudomonas fluorescens* by employing click chemistry for signal amplification and a nanoarchitectonics@Phage-based recognition probe. The biosensor mechanism was initiated through the synthesis of a CCRN@Phage recognition probe with CuSO_4_ and cysteine-RGD, in which EDC and NHS crosslinking was used for functionalisation of the bacteria. Further, in the presence of *P. fluorescens*, concanavalin A-Fe_3_O_4_ NPs and CCRN@Phage formed a sandwich structure and were magnetically isolated to catalyse the CuAAC reaction between AHC azide and BOL alkyne to amplify the fluorescence signal.^77^

A single-molecule biosensor and imaging platform was fabricated for the detection of Hantaan virus by the Xiong team with the implementation of a click-assisted multi-fluorophore nucleic acid probe. Biosensing employed the polymerase chain reaction to synthesise multiple alkynyl-containing DNA with 5′-ethynyl-2′-deoxyuridine-5′-triphosphate, incorporating the 2′-deoxythymidine 5′-triphosphate nucleoside. Subsequently, the CuAAC reaction occurred between the alkyne groups of DNA and the azide-functionalized Cy5 fluorescent signal probe. A reporter unit was synthesised *via* a triazole-conjugated signal probe and a biotinylated capture probe, which was further functionalised onto magnetic beads. In the presence of viral RNA, the binding affinity of the target RNA to the capture probe caused the fluorophore probe to dissociate and amplify the signal. The biosensor reported real-sample detection in HTNV-infected serum, with recovery rates of between 92.91% and 103.66%.^78^

The click reaction, being high yielding and giving a stable product, is often efficient for multiple practical applications, and likewise coumarin derivative's 1,2,3-triazole ring formation exhibits a sharp fluorescent output signal for target detection in biosensor mechanism studies.^79^ Hang *et al.* developed a biosensing technique based on Phage@DNAzyme probe-triggered fluorescent click chemistry for the detection of foodborne pathogens including *E. coli* O157:H7 and other bacteria. Target bacteria were immobilised on a 4-mercaptophenylboronic acid (MPBA)-functionalized gold slide, and further conjugated to Phage@DNAzyme, wherein the catalytic properties of the Phage@DNAzyme triggered the CuAAC between 3-azido-7-hydroxycoumarin (AHC) and 3-butyn-1-ol (BOL), leading to coumarin-1,2,3-triazole formation. This conjugated 1,2,3-triazole with a blue fluorescence signal was observed with a smartphone, proving its application for real samples of pork and milk, with a recovery rate of 91–110% (Fig. 8).^80^

**Fig. 8:**
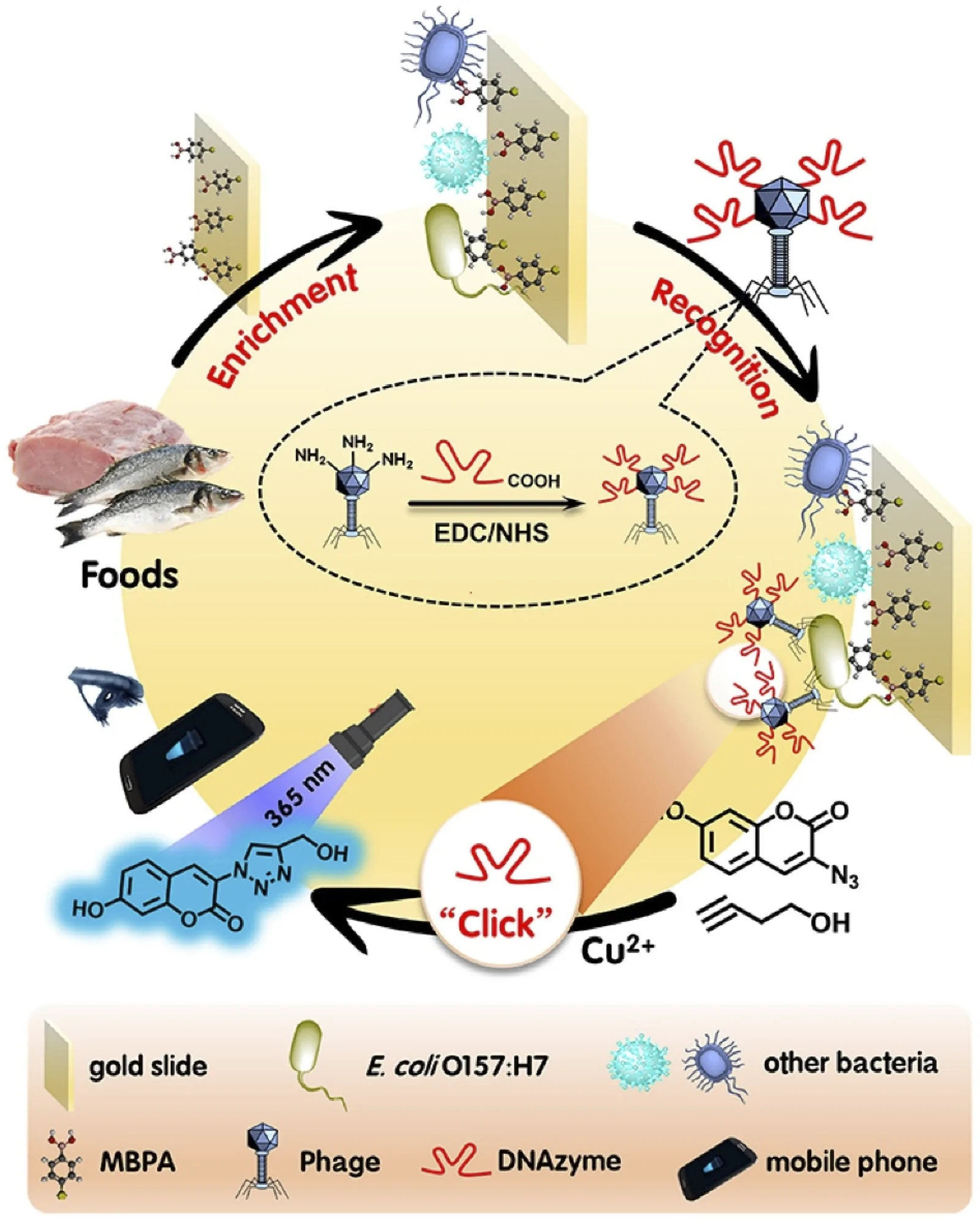
Schematic of the synthesis of a Phage@DNAzyme probe and phage-based bioassay with click chemistry^80^ (adapted/reproduced from ref. 80 with permission from the American Chemical Society, copyright 2023).

#### Electrochemical signal transduction

Electrochemical-based biosensors have powerful sensing potential due to their reduced energy requirement, low cost and high sensitivity. In the electrochemical signal strategy, the target recognition event is converted into measurable electrical signals. 1,2,3-Triazole is used for immobilization of a stable DNA probe or redox-active molecule on an electrode surface. This subsection discusses 1,2,3-triazole-based electrochemical biosensors for different analytes, such as miRNAs and bacteria.

In other studies, Zhou *et al.* constructed an ultrasensitive electrochemical biosensor based on click chemistry-mediated enzyme-assisted miRNA141 detection. The sensor was modulated using an azide-strand and a hairpin-linked alkyne-strand conjugated *via* a 1,2,3-triazole to form a captured DNA (cDNA) probe containing a complete G-quadruplex sequence, resulting in the formation of DNA–RNA hybrid duplexes. The enzyme-assisted target recycling (EATR) reaction was initiated during incubation with hybrid duplexes, and the specific binding of DNA and miRNAs facilitated the release of duplex-specific nuclease (DSN). The DSN demonstrated the capacity to differentiate between well-matched and mismatched duplex DNA, resulting in the cleavage of DNA within the DNA–RNA duplexes, thereby releasing free miRNA to initiate another cycle, yielding exponential signal amplification. Rationally constructed FC60, utilized to change the Au electrodes, therefore enhanced the detection signal. RODs from 1.0 × 10^−7^ µM to 0.1 µM and an LOD of 7.78 × 10^−9^ µM were obtained using the biosensor, which is suitable for early clinical diagnosis, biomedical research, food safety, and environmental monitoring.^81^ The click reaction helped to stabilize the DNA duplex biosensor and provided extra sensitivity; moreover, 1,2,3-triazole bonding ensured reduced background signals and amplified the analytical performance of the biosensor.^81^

Zhou *et al.* worked on an electrochemical biosensor for the detection of *E. coli* in real seawater and milk samples with a LOQ of 1 CFU mL^−1^. The biosensor mechanism involved a signal unit of a black phosphorus nanosheet functionalised with AuNPs and ferrocene-alkynyl groups, which underwent a click reaction in the presence of the target *E. coli*-reduced Cu(i). CuAAC reaction between the azide-Fe_3_O_4_ and the alkyne groups of the signal units resulted in the formation of a complex of Fe_3_O_4_/signal unit to amplify the electrochemical signal.^82^

An electrochemical biosensor was fabricated by the Liu team for CA72-4 detection in whole blood samples. The biosensor implied a SPAAC reaction between an indium tin oxide electrode containing an aptamer/CA72-4 complex functionalized with phenylboronic acid (PBA)-PEG-N_3_ and 4Arm-PEG-dibenzocyclooctyne. Subsequently, 4Arm-PEG-N_3_ was added, which participated in an e-click reaction with ethynyl-ferrocene to introduce the signal probe.^83^

A miRNA-21 biosensing platform was introduced by Yu and co-authors based on visible/near-infrared induced RAFT polymerization. The biosensor mechanism involved indium tin oxide-conjugated azide-containing hairpin DNA, which rearranged its structure when the target miRNA-21 was introduced. Further, a click reaction took place between the heterobifunctional unit with an alkyne moiety and the azido group of the electrode to form a triazole ring. Subsequently, FMMA was conjugated *via N*-succinimidyl 4-cyano-4-(phenylcarbonothioylthio)pentanoate for the reversible addition–fragmentation chain transfer signal amplification technique.^84^

#### Surface-enhanced Raman scattering (SERS) signal transduction

SERS is a surface-sensitive analytical technique that amplifies the weak Raman signals from molecules adsorbed onto the surface of metallic structures. SERS-based biosensors are highly sensitive, rapid, and have multiplex detection potential due to their capability to provide unique vibrational fingerprints for target analytes.^85^ The site-specific nature of 1,2,3-triazole formation results in the conjugation of DNA probes and Raman reporters for substrates. This subtopic mainly covers 1,2,3-triazole-based SERS biosensors.

Zhang and co-authors fabricated a dual-mode biosensor based on surface-enhanced Raman spectroscopy (SERS) and electrochemiluminescence (ECL) for the identification of miRNAs. For miRNA-133a identification in real samples of 1% saliva, 10% urine, 10% cell lysis buffer, and 10% serum samples, the biosensor exhibited a detection range from 1 × 10^**−**10^ µM to 1 × 10^−3^ µM. With detection limits of 3.2 × 10^−11^ µM and 1.7 × 10^−10^ µM in ECL and SERS modes, respectively, the biosensor is suitable for nucleic acid detection in clinical analysis. The biosensing platform functioned using alkynyl-modified C-rich DNA sequences, which were anchored to the AuNP-coated electrode surface. On exposure to the DNA walker, the azide-modified probe and alkynyl-modified C-rich DNA underwent cyclisation. Following the copper(i)-catalysed azide–alkyne cycloaddition, a complete DNA strand linked to silver nanoparticles was generated by 1,2,3-triazole formation, which further coordinated emission of SERS and ECL signal amplification (Fig. 9).^86^

**Fig. 9:**
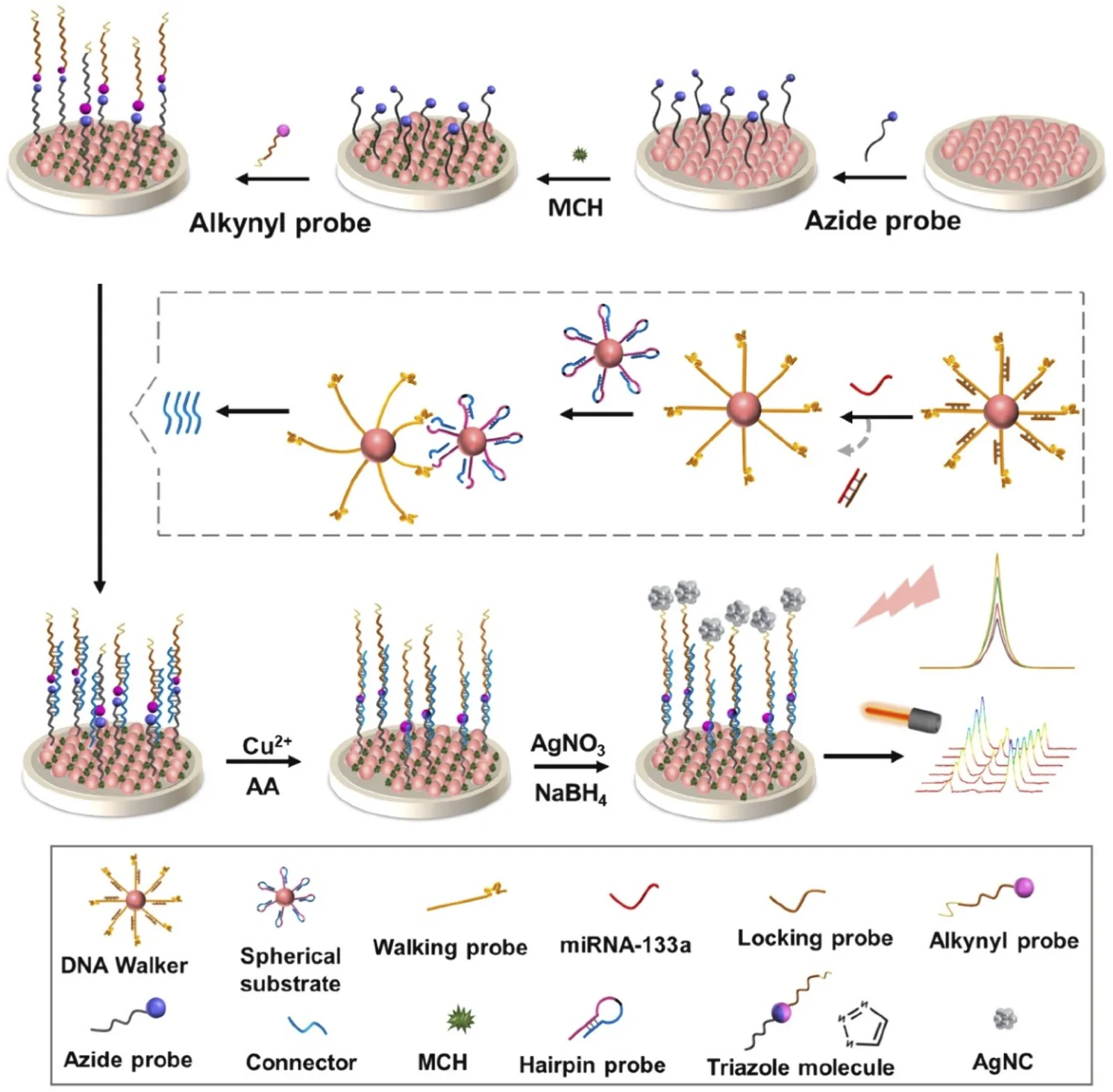
Schematic of the DNA walker and *in situ*-manufactured probe-based SERS-ECL dual-mode biosensor for measuring circulating miRNAs^86^ (adapted/reproduced from ref. 86 with permission from Elsevier, copyright 2024).

Hu and co-workers fabricated an enzyme-free biosensor for surface-enhanced Raman scattering (SERS) based p53 gene detection. This biosensor operates through T-DNA-triggered hybridisation chain reaction (HCR) synthesis of long-strand DNA polymer with Fe_3_O_4_@SiO_2_, p53 gene, and CuNPs, which underwent reduction and acidification. Copper(i) generated during the reaction catalysed the CuAAC between 11-azido-3,6,9-trioxaundecan-1-amine-functionalized magnetic carboxylated magnetic particles and 4-ethynylaniline-functionalized AuNPs. Further, the 1,2,3-triazole-linked chain underwent magnetic separation. Subsequently, in the absence of T-DNA, the click reaction was unable to be triggered, and unreacted alkyne molecules were observed with a Raman signal. The biosensor exhibited an LOD of 1.74 × 10^−8^ µM and a recovery rate ranging from 91.70–112.84% for human serum samples, which validates the biosensor's applicability in clinical applications.^87^

#### Electrochemiluminescence (ECL) signal transduction

In ECL biosensors, the light signal is generated *via* electrochemical reactions, which lowers the interference from background and gives high signal sensitivity.^88^ It combines luminescence change with an electrochemically controlled environment as a response to the target analyte *via* the biorecognition element.^89–91^ This section is focused on ECL biosensors involving 1,2,3-triazole and discusses its widespread applications in biosensing.

Huang and co-authors constructed an ECL biosensor for alkaline phosphate monitoring by employing click chemistry-induced BHCR. The biosensing was initiated when the target ALP hydrolysed the PPi/Cu^2+^ complex formed by a chelating effect and released copper(ii), which underwent reduction with sodium ascorbate and catalysed CuAAC linking of the terminal DNA alkyne and DNA azide to form a longer ssDNA strand *via* 1,2,3-triazole. [Ru(phen)_3_]^2+^ was bound to the dsDNA groove and regained after ultrafiltration, leading to a strong ECL signal. The biosensing method was performed with human serum samples, yielding 95.3–103.6% recovery rates, with an ROD from 0.002 to 50 U L^−1^ and a detection limit of 0.7 mU L^−1^.^92^

Huang *et al.* engineered an ECL biosensor based on click chemistry-triggered hybridization chain reaction for pyrophosphatase monitoring with an 8 mU LOD. The working principle of the biosensor is focused on a pyrophosphate copper(ii) complex, which hydrolysed to provide copper(ii) upon exposure to pyrophosphatase. On addition of sodium ascorbate, copper(i) catalysed the reaction between ssDNA-alkyne and ssDNA-azide to form long T-ssDNA *via* a 1,2,3-triazole linkage. Subsequently, T-ssDNA triggered the HCR to amplify the long l-ssDNA with the aid of HP1 and HP2 probes. The generated probe was immobilized on a gold electrode surface with a molecular beacon [Ru(phen)_3_]^2+^ embedded on ssDNA for strong ECL signals. The biosensor exhibited a recovery rate range from 96% to 105% in serum samples of four clinical arthritic patients. The biosensor provided promising quantitative analysis, which validates its effective usage in treatment and for screening potential PPase inhibitors.^93^

Liu and co-workers developed an ECL biosensor for the detection of miRNA-21 in real human serum with recovery rates varying from 90.0% to 108%. The biosensor involved the CuO-mediated click reaction *via* a catalytic hairpin self-assembly, and was applied in biomedicine and clinical diagnosis with a detection range of 1.0 × 10^−9^ to 1.0 × 10^−3^ µM and a LOD of 2.6 × 10^−10^ µM. The biosensor mechanism involved two hairpin DNAs (HP1 and HP2) labelled with magnetic beads (MBs) and CuO NPs, respectively. Upon the addition of Target miRNA-21, catalytic hairpin self-assembly (CHA) took place with both probes, which resulted in a target recycling cycle and CuO incorporation into the MBs. After magnetic separation, CuO reacted with hydrochloric acid and released copper(ii) ions, which were reduced to copper(i) with ascorbic acid. Copper(i) catalysed the click reaction between the SH-DNA-N_3_^+^ functionalized Au/g-C_3_N_4_-modified electrode and acetylene ferrocene. The 1,2,3-triazole linkage between the g-C_3_N_4_ electrode and ferrocene provided a quenching effect, which was used to quantify the miRNA-21.^94^

### Signal amplification strategies

Signal amplification strategies play a significant role in sensing studies and can improve the sensitivity for detecting the target at trace concentrations.^95^ Different amplification strategies result in different analytical performances by changing the single step of target recognition into multiple events of signal amplification. 1,2,3-Triazole can be combined with different amplification techniques due to its efficient binding potential for DNA probes and other materials.^37,39,96^ In this section, among many amplification strategies, G-quadruplex-based signal amplification, CRISPR-based signal amplification, ATRP-based signal amplification, nanomaterial-assisted signal amplification, and DNA structure-based signal amplification are discussed.

#### G-quadruplex-based signal amplification *via* 1,2,3-triazole

Four DNA strands with guanine-rich (G) bases are used for the fabrication of DNA-quadruplexes, which have been utilized in biosensor mechanisms. Such systems have unique structural features, are highly stable, and enable robust signal amplification, providing versatile sensing tools. G-quadruplexes exhibit structural polymorphism and can adopt different conformations, such as parallel, antiparallel, and hybrid forms, depending on the environmental conditions and sequence conformations.^97,98^ Notably, G-quadruplexes are stable, and hemin (iron protoporphyrin IX) tucks into the G-quadruplex structure, creating a specific binding pocket. This complex acts like horseradish peroxidase, catalysing the oxidation of hydrogen peroxide to produce colorimetric signals with excellent detection as a potential biosensing method.^99,100^

Recently, 1,2,3-triazole-linked DNA strands capable of forming G-quadruplexes gained attention as they bridge the interface between chemical modulation and biological functionality.^101^ 1,2,3-Triazole-assisted G-quadruplex-based biosensors are relatively new in the field of target detection. Wang *et al.* developed an enzyme-free fluorescent DNA detection platform employing two nucleic acid-templated azide–alkyne cycloaddition (AAC) reactions catalysed by a heterogeneous Cu_2_O nano-catalyst. The analytical method involved nucleic acid-templated click ligation linear amplification reaction (NA-CLLAR) and nucleic acid-templated click ligation exponential amplification reaction (NA-CLEAR) methods. The NA-CLLAR method utilises a G-rich azide-modified probe P1_G_ and a G-rich alkyne-modified P2_G_ probe combined with the target. Further, in the presence of the Cu_2_O heterogeneous nano-catalyst, the CuAAC was triggered to form 1,2,3-triazole. Upon P1_G_–P2_G_ product interaction with the fluorescent dye thioflavin T (ThT), G-quadruplex/ThT complexes were formed that exhibited high-intensity fluorescence signals. After denaturation, the target was separated from the probe, thereby making the P1_G_–P2_G_ product reusable in cycle II and cycle III and exhibiting 2*n* exponential amplification during thermal cycling.^102^

Peng and co-authors developed a biosensor for lead(ii) ion monitoring in aquatic products based on click chemistry, with a detection limit of 1.245 × 10^−2^ µM. The biosensor mechanism was initiated through a 3′-azide-modified ssDNA sequence, and a C-terminal-modified alkynyl group triggered the formation of a 1,2,3-triazole, resulting in aptamer-peptide conjugate (APC) formation. In the presence of lead(ii) ions, the aptamer domain of the APC specifically formed a G-quadruplex, whereas photoinduced electron transfer (PET) between the G-quadruplex and lead(ii) ions reduced the fluorescence signal. Swimming crab food samples were analysed, and recovery rates ranging from 81.16% to 106.70% were reported, demonstrating its suitability for food monitoring.^103^

The Ren research group designed a colorimetric immunoassay for the detection of AFB_1_ in food, with a ROD of 100 pg mL^−1^–50 ng mL^−1^ and a limit of detection of 26.23 pg mL^−1^. The sensor employed a signal probe synthesised *via* PDA-modified UiO-66-NH_2_ and covalently bonded to AFB_1_ antigen (AFB_1_-BSA). The target PUA probe was further modified with copper(ii) ions, which were reduced to copper(i), triggering the click reaction between ssDNA-alkyne and azide-modified short DNA to form a G-quadruplex DNAzyme. The sensor was used to analyse peanuts and maize samples, with recovery rates of 81.63–112.21% and 80.93–113.95%, respectively (Fig. 10).^104^ Apart from the calorimetric mode, G-quadruplex structures can exhibit enhanced fluorescence signals in the presence of suitable fluorescent dyes. Interactions between the quadruplex and the dye can increase the structural rigidity and reduce conformational flexibility, which results in an enhanced fluorescence signal.^105^

**Fig. 10:**
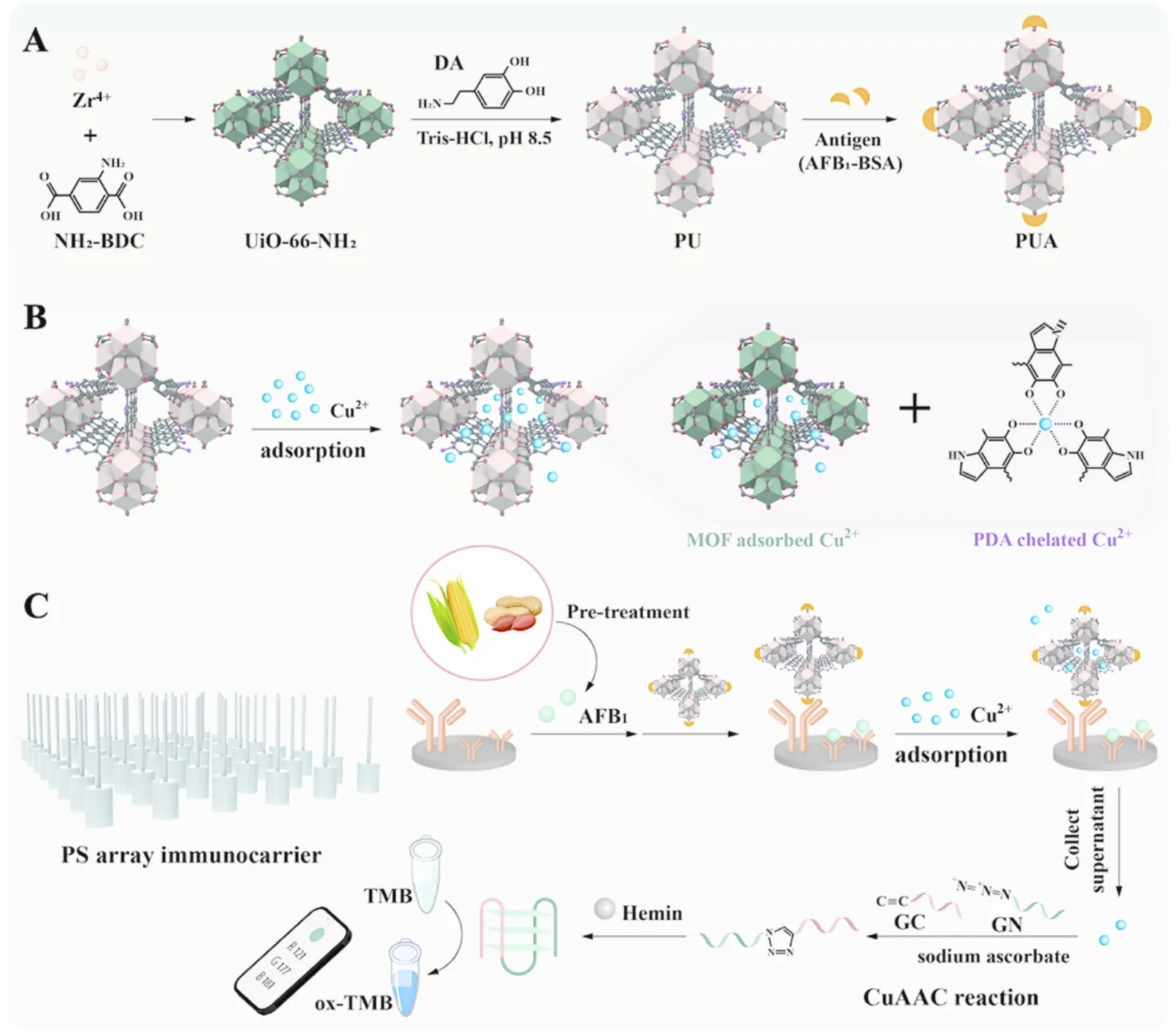
Schematic of the colorimetric immunoassay technique based on PDA-modified UiO-66-NH_2_ and PS array immunocarrier. (A) PUA probe construction procedure. Schematic of (B) UiO-66-NH_2_ and PDA in the PUA probe to synergistically determine copper(ii) ion levels and (C) colorimetric immunoassay method for identifying AFB_1_ in peanut and maize samples^104^ (adapted/reproduced from ref. 104 with permission from Elsevier, copyright 2025).

G-quadruplexes have the potential to enhance electrochemical signals *via* the inherent electroactivity of guanine bases through an oxidation process that changes the structure or form of the G-quadruplex. The compact and solid π-stacked G-quartet arrangement minimizes guanine exposure to the electrode and shifts oxidation to higher potentials, altering the electron-transfer kinetics.^106^ Quadruplex structures act as reactive signal transducers, where the folding or unfolding induced by metal ions, biomolecules, or target binding events results in measurable alterations of the electrochemical signals.^98^ Furthermore, the formation of hemin–G-quadruplex DNAzyme complexes provides catalytic redox functionality, leading to improved electrochemical signals in biosensing applications.^97^

An electrochemical biosensor constructed by Wei *et al.* relied on the dual signal amplification of Cu_3_(PO_4_)_2_-mediated click chemistry and DNAzymes, finding application in the detection of pathogenic bacteria (*S. typhimurium*) with a detection limit of 10 CFU mL^−1^. The biosensor employed aptamer-modified magnetic beads (MBs) and concanavalin A–Cu_3_(PO_4_)_2_ hybrid nanoflowers to capture the target bacteria, thereby releasing copper(ii) ions, which are reduced to copper(i) in the presence of ascorbic acid to catalyse the click reaction. 6-Mercapto-1-hexanol and ssDNA G1-modified alkyne immobilised on a gold electrode underwent a click reaction with azide-functionalized ssDNA G2. Further, through 1,2,3-triazole linkage, the G1 and G2 fragments formed a G-quadruplex and produced electrical signals such that the biosensor yielded 101–107% recovery rates for *S. typhimurium* detection in milk.^107^

Tai Ye *et al.* designed a copper detection platform based on a renewable DNA tetrahedron interface. Detection was facilitated through the synthesis of a tetrahedron DNA nanostructure from initial material as strands TA, TB, TC, and TD. After the nanostructure was immobilized on a gold electrode surface, azide groups were added to further functionalize it. An alkyne-modified split G-quadruplex fragment (G2) and an azide-functionalized tetrahedron DNA nanostructure underwent a CuAAC reaction in the presence of the target copper(ii) ions and sodium ascorbate, building a G-quadruplex–hemin complex stitched together by 1,2,3-triazole linkages. This complex then promoted the oxidation of TMB in the presence of hydrogen peroxide and increased the electrochemical signal. In performance analysis assays, grape juice and milk samples were examined, and the biosensor exhibited recovery rates ranging from 92.96–102.02% and from 103.61–110.75%, respectively.^108^

#### CRISPR-based signal amplification with the aid of click chemistry

Nobel Prize-winning CRISPR technology, together with CRISPR-associated (Cas) proteins, performs cleavage of invading nucleic acids, providing the capability to alter genetic material.^109,110^ It functions through Cas endonucleases, which are directed to complement target sequences by CRISPR-derived RNAs, resulting in precise nucleic acid cleavage.^110,111^ Of the class 2 CRISPR systems, Cas12a (Type V-A) is particularly suitable for biosensing due to the single CRISPR RNA needed and the target-activated *trans*-cleavage activity toward ssDNA. This specific activity utilises CRISPR for biosensing and enables sensitive, selective diagnostic platforms.^111–113^ When click chemistry is integrated with CRISPR systems, it has the potential to result in biosensing applications.

Yu *et al.* developed a biosensing technique based on CRISPR/Cas12a with click immunoassay for *T. spiralis* identification with recovery rates between 92.61–102.28% in pork samples. The biosensor uses a sandwich structure to detect *T. spiralis* in pork samples, with polyclonal antibody-conjugated magnetic beads (MBs@PcAb) and gold nanoparticles functionalized with ssDNA and monoclonal antibodies (AuNP@ssDNA/McAb). Further, ssDNA T20 on AuNPs was cleaved in the CRISPR/Cas12a system. *In situ*, Cu_2_O nanoparticles were synthesised with ssDNA T20 as a template, which served as a clickase to trigger the CuAAC reaction. In the presence of sodium ascorbate and Cu_2_O nanoparticles, azide 1 and alkyne 2 hybridised to form 1,2,3-triazole, which resulted in a fluorescence signal. The biosensor exhibited outstanding quantitative performance with ROD and LOD values ranging between 3.125 and 100 ng mL^−1^–0.35 ng mL^−1^, respectively. This approach utilized 1,2,3-triazole for the signal, making it simple, efficient and sensitive, highlighting its potential for practical applications in food monitoring.^114^

Wei *et al.* engineered a biosensor utilising CRISPR/Cas12a-based magnetic relaxation switching for detection of methicillin-resistant *S. aureus* (MRSA) in food. This biosensor uses streptavidin immobilized on carboxyl modified MNP_180_, which was further attached to a biotin-containing circle padlock probe. Subsequently, DNA-alkaline phosphatase (ALP), DNA-polymerase, and MNP_180_ hybridised to form MNP-poly-ALP *via* on-particle rolling circle amplification (RCA). Subsequently, target recognition of the *mecA* gene from the MRSA genomic DNA, which was extracted from real samples, activated the CRISPR/Cas12a for *trans*-cleavage. Initially formed MNP-poly-ALP was cleaved through the dsDNA part by CRISPR/Cas12a. *Trans*-cleavage activity triggered the release of ALP, which hydrolysed the nonreductive 2-phospho-l-ascorbic acid trisodium salt (AAP) to ascorbic acid (AA). Copper(ii) was reduced *via* AA to catalyse the click reaction. The 1,2,3-triazole linkage formed the MNP_30_–MNP_1000_ complex *via* azido-MNP_30_ and alkyne-MNP_1000_ through CuAAC. Magnetic separation was used to separate M1000, which exhibited high saturation magnetization, whereas M30 was used for T2 signal because of its low saturation magnetization. Eggs, milk, and pork samples were analysed, and the recovery rates were 75–112%, 82–104% and 81–91%, respectively, demonstrating the potential of the biosensor for the detection of foodborne drug-resistant bacteria.^115^

The same research group, in another study, developed a biosensor for methicillin-resistant *S. aureus* (MRSA) *via* enhanced CRISPR/Cas12a fluorimetry and a DNAzyme-embedded framework nucleic acid (FNAzyme) substrate. The biosensor mechanism involves MRSA captured by an aptamer immobilized over magnetic nanoparticles, which led to the release of an ssDNA activator. The ssDNA activates the crRNA-cas12a to trigger the *trans*-cleavage activity. The tetrahedral framework nucleic acid, containing multiple embedded CLICK-17 DNAzymes, accelerated *trans*-cleavage and deactivated all the DNAzymes, which enables the formation of the triazole ring, whereas in the absence of the target, DNAzymes are more available, which facilitates the CuAAC reaction between AHC and BOL to form the fluorescent coumarin 1,2,3-triazole ring derivative and amplifies the fluorescence. This highlights the benefits of the biosensor with respect to target recognition, sensitivity and selectivity. The feasibility of the biosensor was demonstrated in food samples of eggs, milk and pork. The biosensor exhibited low detection limit of 18 CFU mL^−1^, and recovery rates varied from 80% to 101% (Fig. 11).^116^

**Fig. 11:**
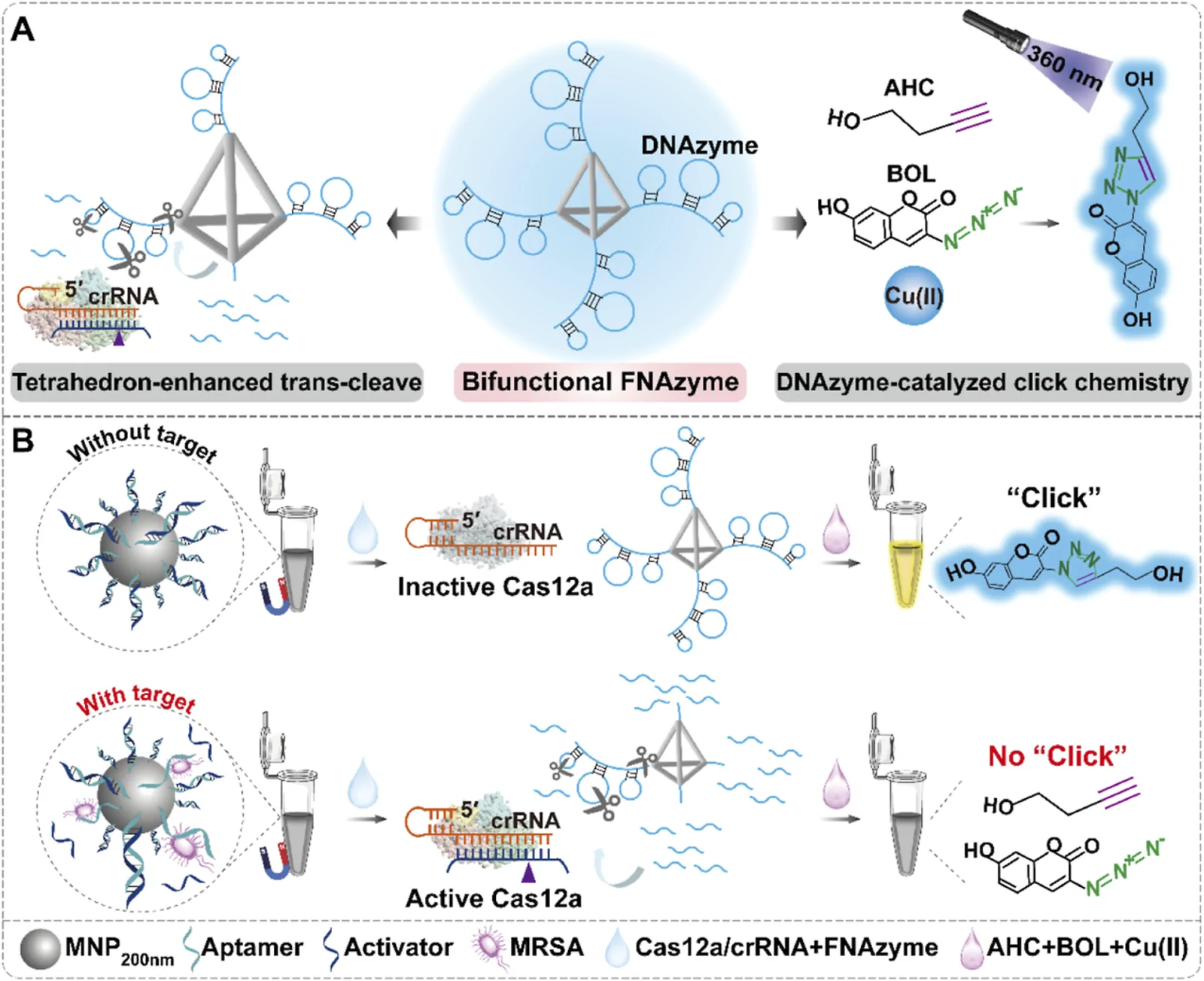
FNAzyme-enhanced CRISPR/Cas12a fluorimetry schematic diagram. (A) FNAzyme's dual functionality schematic. (b) FNAzyme-enhanced CRISPR/Cas12a fluorimetry for MRSA detection^116^ (adapted/reproduced from ref. 116 with permission from the American Chemical Society, copyright 2024).

Qiu and co-authors designed a fluorescence biosensor using CRISPR/Cas12a with the CLICK17-mediated CuAAC for *Salmonella enterica* (*S. enterica*) detection in spiked milk, orange juice, and meat samples and reported a recovery rate of 93–104%. The detection process relied on recombinase polymerase amplification to amplify the target *invA* gene of *Salmonella*. The crRNA sequence bonded to the *Salmonella* target gene, triggering the activation of the Cas12a system. The activated Cas12a system *trans*-cleaved the CLICK-17, which acted as ssDNA. The resulting cleaved CLICK-17 with an incomplete catalytic active structure was unable to catalyse the click reaction, which amplified the weak fluorescence signal. In the absence of the *S. enterica* target, un-cleaved intact CLICK-17 catalysed the click reaction between 3-azido-7-hydroxycoumarin and 3-butyn-1-ol, forming 1,2,3-triazole and leading to a strong fluorescence signal, and the *trans*-cleavage of the CLICK-17 DNAzyme results in sensitive target recognition, making it a perfect combination for biosensing.^117^

#### DNAzyme-mediated 1,2,3-triazole formation for biosensing

In recent years, a 79-nucleotide DNA enzyme, or DNAzyme (deoxyribozyme), named ‘CLICK-17’, was isolated and characterised, which exhibited catalytic activity with high efficiency for CuAAC conjugation at sub-micromolar concentrations of Cu(i) ions, without a reducing agent, in aqueous media. The electrochemical studies for the Cu(ii)/Cu(i) system show that DNAzyme favours Cu(i) over Cu(ii) ions using single-stranded DNA that forms a specialized cavity using its nitrogenous bases and phosphate backbone to encapsulate Cu(ii) ions and reduce them to Cu(i) ions, in aqueous media. The enhanced activity of CLICK-17 arises from the formation of a rigid and well-defined DNA-based ligand environment of the active site, which promotes efficient catalytic cleavage by facilitating the binding and redox stabilisation of catalytically important copper(i) ions.^118,119^ The useful applications of CLICK-17 in catalytic and biosensing platforms have been the subject of numerous studies.

Wang *et al.* worked on an immunosensor based on a DNAzyme-assisted click reaction for *S. aureus* detection for a point-of-care test in foodborne bacteria screening. A 4-mercaptophenylboronic acid (4-PMBA)-functionalized stir bar, integrated with IgY and copper(ii)-labelled polydopamine (PDA) nanoprobes containing an *S. aureus* sample, resulted in a sandwich-structured complex. A click reaction occurred between the alkyne group-labelled DNAzyme (CLICK-C2) and streptavidin–biotin-azido (Str-N_3_) to form 1,2,3-triazole in the presence of copper(ii) ions released at pH 6. The detection occurred through DNAzyme-streptavidin complexes using microfluidic chips that realised ROD values in the range of 10 to 2.5 × 10^4^ CFU mL^−1^ and an LOD of 3 CFU mL^−1^. The detection in real samples (animal origin, milk, pork, and fish) showed recovery rates of 90.0% to 105%, with no interference and high selectivity.^120^

Liu *et al.* engineered an electrochemical biosensor for total copper detection with LODs of 3.5 × 10^−3^ and 8.0 × 10^−4^ µM for copper(i) and copper(ii) ions, respectively. The sensor mechanism employs gold slides with self-assembled monolayers of 11-azido-1-undecanethiol and 1-octanethiol. Furthermore, the azide group underwent a click reaction with alkyne-tagged DNAzyme in the presence of CLICK-17 and copper(ii) or copper(i) to form the 1,2,3-triazole linkage. The negatively charged phosphate backbone of the DNA strands on the electrode surface formed an electrical connection with [Ru(NH_3_)_6_]^3+^, which was quantified by its cyclic voltammetry response. CLICK-17, being a reducing agent, can reduce copper(ii) to catalyse CuAAC (Fig. 12).^121^

**Fig. 12:**
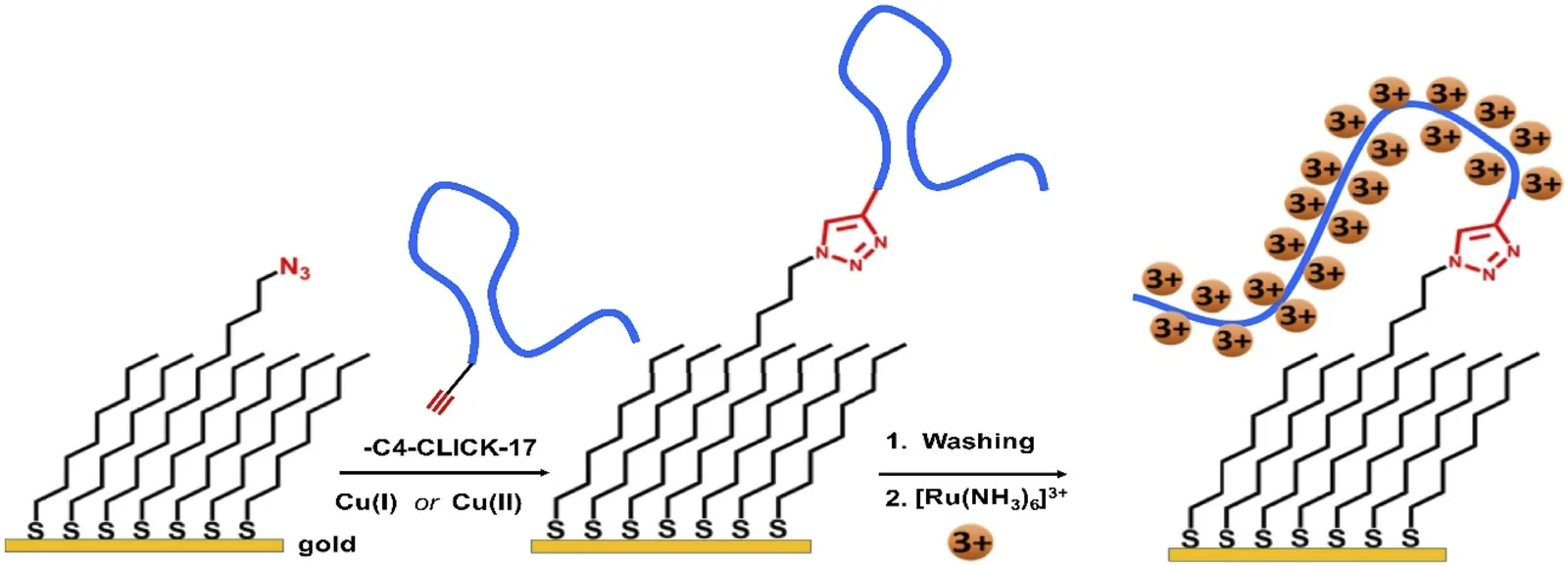
Schematic of the electrochemical sensing of total copper (Cu(i)/Cu(ii)) using the in-*cis* coupling of hexynyl CLICK-17 DNAzyme (≡-C4-CLICK-17) N3C11S-/C8S-Au^121^ (adapted/reproduced from ref. 121 with permission from Elsevier, copyright 2021).

Yan *et al.* developed a colorimetric sensing technique employing DNAzyme CLICK-17 for CuAAC. The sensing mechanism relied on the catalytic potential of CLICK-17 to reduce the target copper(ii) to copper(i) and catalyse the click reaction between azide-modified gold nanoparticles (azide-AuNPs) and alkyne-capped dsDNA (alkyne-linker DNA). By the formation of a 1,2,3-triazole linkage within the azide-AuNPs/alkyne linker-DNA/CLICK-17 probe solution, the colour changed from ruby red to bluish purple, with recovery rates of 90.8% and 99.8% in mineral water.^119^

Wang *et al.* constructed a dual-mode and dual-target biosensor for the detection of pathogenic bacteria in milk, pork, fish, and lettuce samples which involved ‘signal probes’ that were generated through two amino-functionalized Zeolitic Imidazolate Framework-8 conjugated Fc and MB with DNAzyme. Subsequently, *E. coli* and *S. typhimurium* were captured through MPBA-modified electrodes and hybridised to generate signal probes that resulted in electrochemical signals. Separately released DNAzyme catalysed the click reaction between 3-azido-7-hydroxycoumarin (AHC) and 3-butyn-1-ol (BOL), to form a 1,2,3-triazole ring derivative, which amplified the fluorescent signal. The recovery rates in real-life samples were 95.0% to 106% and 90.0–110% for *E. coli* and *S. typhimurium*, respectively, and the biosensor exhibited LODs of 5 CFU mL^−1^ and 8 CFU mL^−1^ within 30 min, respectively, demonstrating its capability in the field of food safety (Fig. 13).^122^

**Fig. 13:**
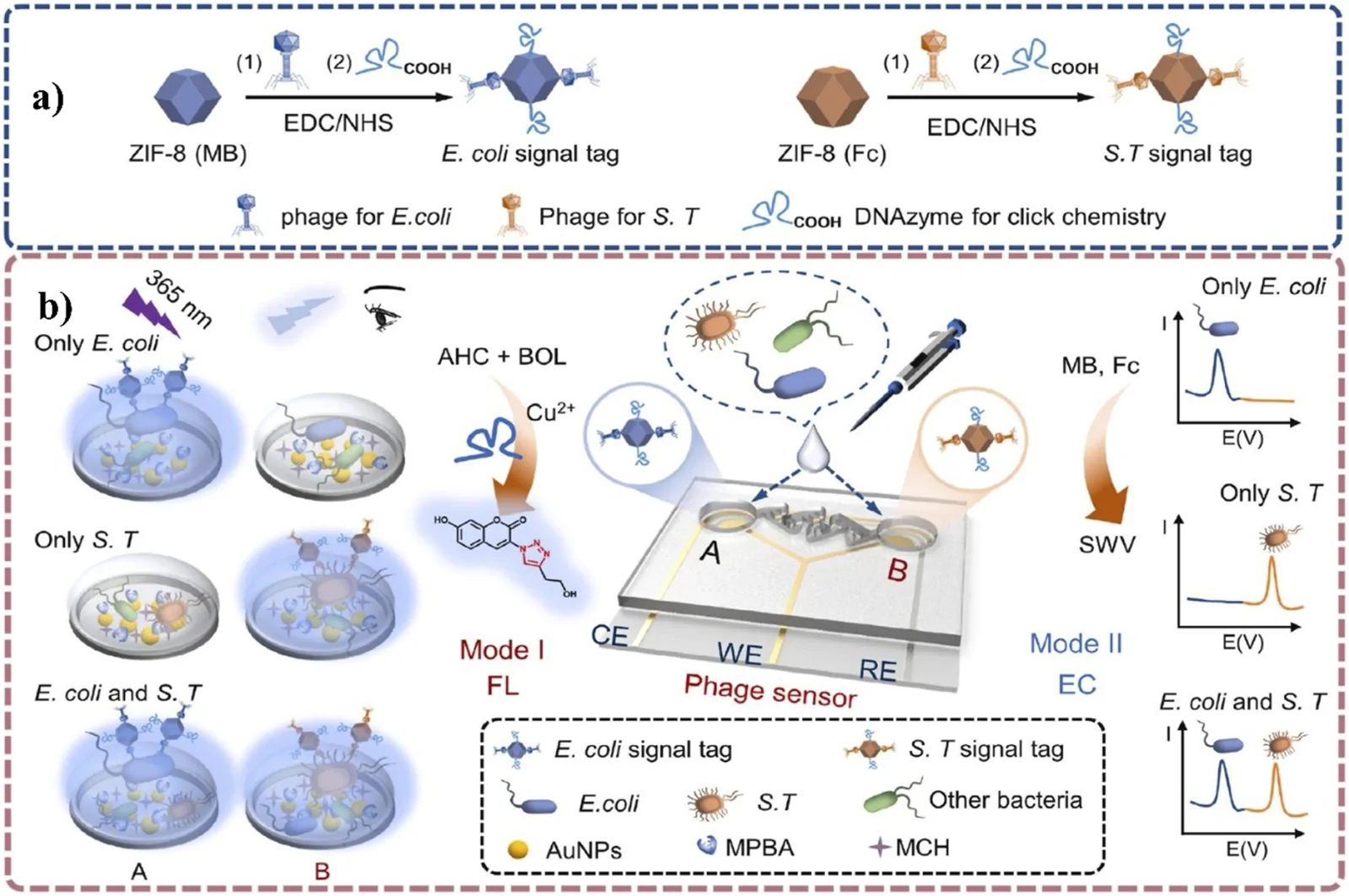
(a) Process used for generating tag-encoded signals and (b) suggested dual-mode and dual-target biosensor for the simultaneous detection of *E. coli* and *S. typhimurium* with the help of CLICK-17 (ref. 122) (adapted/reproduced from ref. 122 with permission from Elsevier, copyright 2023).

Li *et al.* engineered a dual-mode detection technique for glyphosate in tap water and soybean samples, wherein the biosensor was based on the formation of a coordination complex between glyphosate and copper(ii) ions, which failed to facilitate the click reaction between AHC and BOL due to the low concentration of copper(ii) ions. In the absence of glyphosate, the click reaction takes place with the help of DNAzyme and sodium ascorbate (SA) to yield a fluorescent coumarin 1,2,3-triazole derivative ring. In another mode, glyphosate inhibits the enzyme-like catalytic sites on CeO_2_, and unreacted DNAzyme is absorbed on the CeO_2_. The inhibition of the enzyme activity reduces the oxidation of 3,3′,5,5′-tetramethylbenzidine (TMB), which results in a colorimetric change, with a recovery range of 90.89–105.06% that is comparable to HPLC recovery rates, demonstrating the utilisation of the biosensor in pesticide residue detection.^123^

The competitive evaluation of CLICK-17 and CLICK-T by Wu *et al.* provides a better approach for using DNAzyme catalysis for the detection of copper(ii) ions in natural water. The detection platform is initiated with a CLICK-T functionalized hydrophilic paper-based analytical device (PAD) to work as a detection surface (Fig. 14A). When the target and AHC and BOL containing solution was introduced, the PADs produced fluorescent signals (Fig. 14B). The sensing method exhibited a limit of detection of 0.1 µM and recovery rates of 89–102% in real water samples, and the catalytic performance of CLICK-T was assessed in relation to CLICK-17 (Fig. 14C). Its shorter sequence length likely allowed CLICK-T to facilitate CuAAC more rapidly and effectively than CLICK-17, primarily because it provided better access for analytes to the active site. This enhanced catalytic effectiveness allowed CLICK-T to improve PDA-based detection methods by increasing the reaction efficiency.^124^

**Fig. 14:**
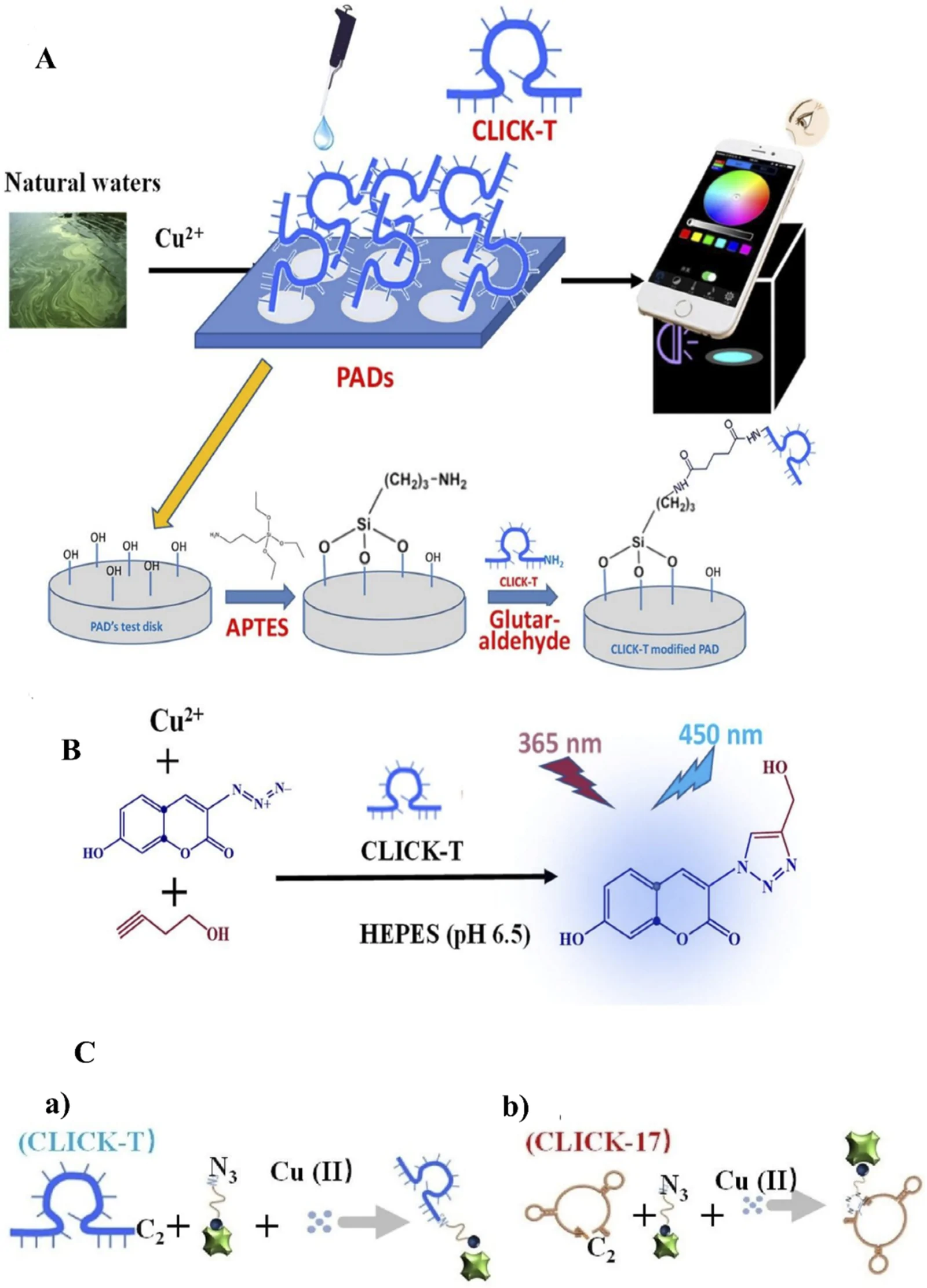
(A) 3-Azide-7-hydroxycoumarin interacting with 3-butyn-1-ol (BOL) in the presence of copper(ii) on CLICK-T-modified PADs, resulting in the emission of fluorescence visible to the naked eye and measurable with a smartphone. (B) Mechanism behind the development of fluorescence. (C) CuAAC reaction facilitated by (a) CLICK-T and (b) CLICK-17 initiated by copper(ii), along with the alkyne-modified CLICK-17 and CLICK-T and streptavidin, carrying an azide group^124^ (adapted/reproduced from ref. 124 (open access paper used under the terms of the Creative Commons Attribution (CC BY) license) with permission from Elsevier, copyright 2022).

This approach enhances CuAAC-assisted biorecognition applications by boosting biosensor sensitivity and stability without additional reductants. Additionally, CLICK-17 and CLICK-T DNAzymes serve as more environmentally friendly options compared to traditional chemical catalysts, suggesting that they could be safer alternatives for CuAAC-based biosensing systems.

#### Click chemistry-aided atom transfer radical polymerisation (ATRP) based signal amplification

For the production of polymers with predictable chain length, distinct functionalities, specific compositions, and narrow molecular weight distributions, the ATRP technique is widely used. ATRP is a controlled radical polymerization (CRP) technique that is initiated by the reaction of an alkyl halide initiator with a low-oxidation-state transition-metal complex, typically a copper(i) complex. This reaction generates a propagating radical and a higher-oxidation-state metal complex that acts as a deactivator. The rapid and reversible activation–deactivation equilibrium ensures controlled chain growth of polymers.^125,126^

1,2,3-Triazole conjugation is used to introduce functional groups and molecular building blocks in ATRP by terminally functionalizing halide chains with azide or alkyne fragments. The use of the high-yielding and modular CuAAC reaction with ATRP-derived polymerisation results in the effective creation of bioconjugates, surface-grafted polymers, and block and graft copolymers. In some studies, ATRP and CuAAC take place simultaneously in a single reaction system, using the same copper catalyst for both click coupling and controlled radical polymerization. This 1,2,3-triazole-assisted ATRP is used in biosensing for signal amplification and holds high potential for utilization in scientific fields.^127–129^

Zhao and team used a similar ATRP for RNA detection, but vitamin B_12_ Co(i) was used as a catalyst for ATRP. The biosensor was highly selective, with appropriate behaviour for the detection of RNA in complex biological materials (Fig. 15).^130^ Li *et al.* used the ATRP approach for the detection of *E. coli*, which was driven by the reduction of copper(ii) to copper(i) to catalyse the CuAAC and initiate the ATRP mechanism to activate the polymer chain for signal amplification.^131^

**Fig. 15:**
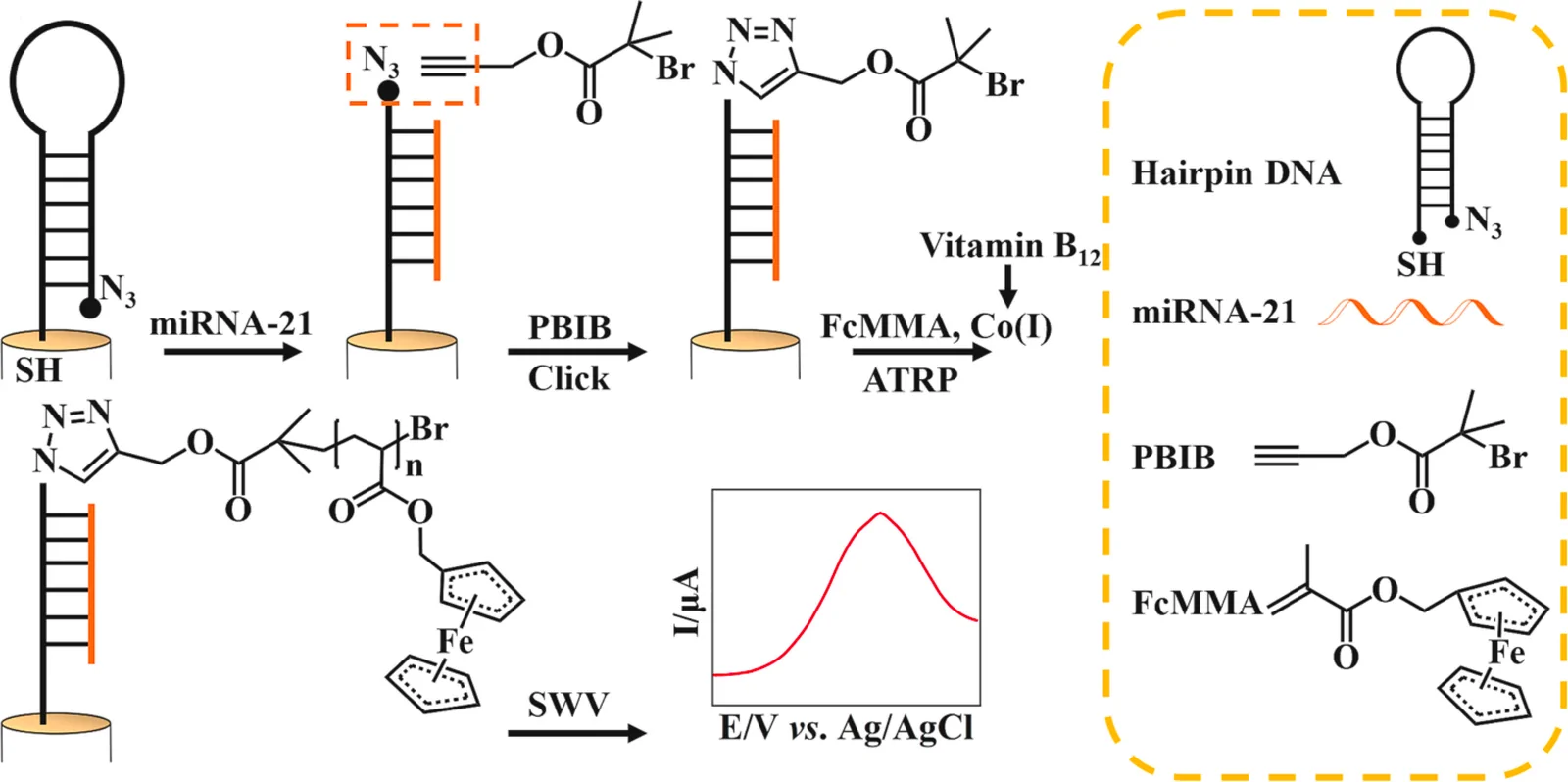
Schematic of an electrochemical RNA biosensor using the click reaction and ATRP idea^130^ (adapted/reproduced from ref. 130 with permission from Elsevier, copyright 2023).

Sun and co-workers designed an electrochemical biosensor for nucleic acid detection based on ATRP with a detection limit of 1.954 × 10^−12^ µM and a range of detection of between 1 × 10^−11^ and 1 × 10^−5^ µM. The analytical features of the biosensors involved Hairpin DNA (HP-DNA) functionalized over the surface of a gold electrode, with the assistant DNA (a-DNA) attached to the gold electrode surface forming a triple helix structure. As the target DNA afforded assistant DNA (a-DNA), it initiated the Exo III-mediated target cycle, which led to the destruction of the triple helix structure, exposing the azide to the AuE surface. Propargyl-2-bromoisobutyrate (PBIB) was linked to HP-DNA containing azide through 1,2,3-triazole *via* click chemistry. Polymerization occurred for signal amplification with FMMA *via* electrochemical ATRP and was quantified using square-wave voltammetry (SWV). The biosensor exhibits remarkable interference resistance when analysing DNA in serum samples, along with ultra-sensitivity, high precision, and strong selectivity.^132,133^

Sun and co-workers used ATRP for the detection of methamphetamine in a very unique manner. The methamphetamine-binding aptamer (A-DNA) and its complementary strand (S-DNA) were first hybridized, with S-DNA fixed over the electrode surface. Methamphetamine caused A-DNA to preferentially bind to the target molecule and dissociate from S-DNA, exposing the electrode surface. Upon the addition of methamphetamine, A-DNA bound to the methamphetamine and was released. Subsequently, azide DNA was conjugated over the electrode surface to form 1,2,3-triazole with PBIB, and ATRP took place.^134^

Zhang *et al.* used a click-assisted ATRP platform for digoxin therapeutic monitoring in clinical samples. These biosensors outperformed ELISA, FPIA and LC-MS/MS, with an LOD of 0.59 pM, demonstrating high sensitivity and low cost.^135^ Wang *et al.* synthesised a fluorescence biosensor *via* ATRP for tobacco mosaic virus RNA in real samples.^136^

#### Nanomaterial-assisted signal amplification

The chemical properties of nanomaterials, such as large surface area, high conductivity and catalytic activity, make them useful for many research areas.^137,138^ Thus, many nanomaterials have tendency to interact with DNA probes^139^ and when stitched with 1,2,3-triazole, these nanomaterials acts as excellent biosensors.

Huo *et al.* synthesised an electrochemical biosensor based on ATRP with a limit of detection of 6.0 × 10^−11^ µM to detect the *mecA* gene in milk and water, with recovery rates of between 87.82–118.78%. The biosensor mechanism was initiated through HCR in the presence of *mecA* gene functionalized magnetic beads (MBs), H1 and H2 probes and streptavidin–copper hybrid nanoflowers (SA@Cu HNFs). SA@Cu HNFs released copper(i) through reduction and initiated a click reaction between the azide-functionalized screen-printed electrode and propargyl 2-bromoisobutyrate (PBIB) to form 1,2,3-triazole. Further, a long polymer chain of (FMMA) was formed *via* ATRP as a signal probe.^140^

Pan *et al.* fabricated an ECL biosensor based on a DNA walker-based target amplification strategy for ALP detection. The biosensing approach involved copper nanoclusters confined in the poly-l-Cys film, which served as an ECL luminophore. Upon introduction of ALP, sodium l-ascorbyl-2-phosphate was converted into ascorbic acid (AA), which catalysed the CuAAC reaction. The click reaction between alkynyl-DNA, P1 and a single-chain containing azido-DNA, P2, formed a longer single chain, W1, *via* 1,2,3-triazole. The DNA strand initiated the opening of the hairpin H1 on the surface of the electrode, exposing the single-strand fragment to conjugate with the quenching probe. A DNA strand labelled Fc was released under the influence of nicking endonuclease, with recovery rates of 95.06–106.2% in human serum. This biosensor exhibited the potential for real-life analysis and detection of non-nucleic acid targets and the diagnosis of diseases.^141^

Shangguan *et al.* designed an aptamer-fluorescent silica nanoparticle label for the identification of *S. aureus* in spiked water samples. The working principle of the biosensor is centred on a CuAAC reaction between azide-conjugated fluorescent silica nanoparticles and an alkyne-functionalized *S. aureus* aptamer, leading to 1,2,3-triazole linkage. Further, *S. aureus* aptamer-nanoparticle bioconjugates (Apt *S. aureus*/FNPs) bound to the target *S. aureus*. With the aid of a positive dielectrophoresis-driven online enrichment and fluorescence microscopy system, the fluorescence signal was amplified. The biosensor exhibited LODs of 93 and 270 CFU mL^−1^ for *S. aureus* in deionised water and spiked water samples, respectively, and a recovery rate of between 69.4% and 97.1%. Quantitative characterization of the biosensor demonstrated great promise for pathogenic bacteria detection in biomedical and biotechnological areas.^142^

Sarıoğulları and co-authors designed an electrochemical sensor based on single-walled carbon nanotubes (SWCNTs) modified with three different BODIPY (boron dipyrromethene) units through 1,2,3-triazole. The azide-containing SWCNTs underwent click reactions with different alkyne terminals, yielding products capable of detecting guanine and adenine in calf thymus DNA with detection limits of 1.07 µM and 2.91 µM, respectively. Guanine and adenine were electrochemically oxidized on the SWCNT-BODIPY hybrid-modified glassy carbon electrodes, which allowed the sensing to take place.^143^

Tian and co-workers worked on a detection method for copper(ii)-reducing bacteria in the range of 10^1^ to 10^7^ cells, and a detection limit of 10^1^ cells based on click chemistry. The detection process is based on a sterile and closed microreactor incorporated in a coverslip functionalized with the azide moiety. Certain bacteria can convert copper(ii) to copper(i) by bonding alkyne-functionalized gold nanorods to the sensing interface *via* a CuAAC reaction. Both Gram-negative *E. coli* (including KPC-2-expressing antibiotic-resistant strains) and Gram-positive *S. aureus* were used to test the biosensor. The 1,2,3-triazole ring system demonstrated several benefits, including high efficiency and outstanding biocompatibility, clear imaging of nonspecific scattering signals, ease and portability in both detection and preparation, as well as rapid (within 3 hours) and sensitive quantification of bacteria (Fig. 16).^144^

**Fig. 16:**
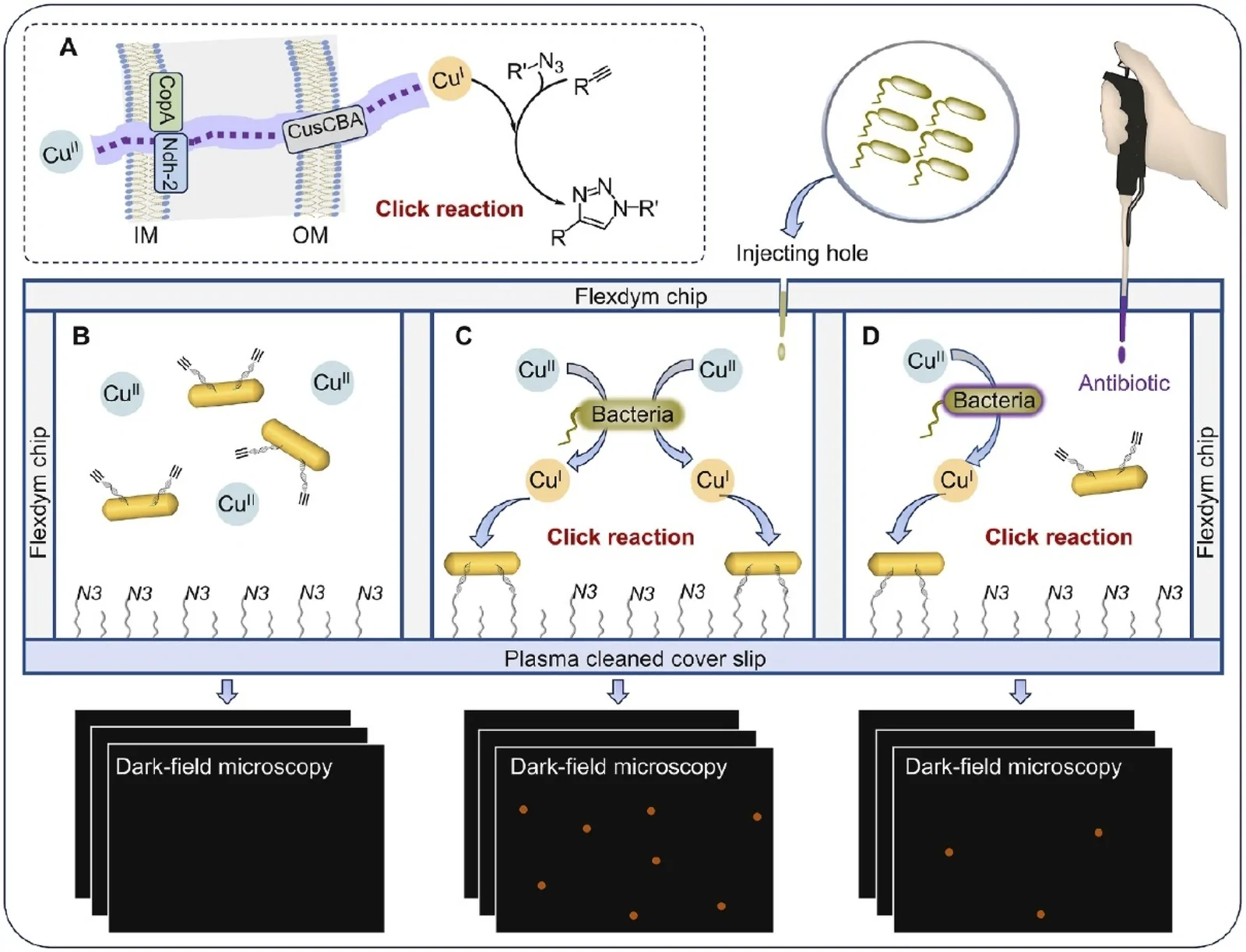
Pictorial illustration of the bacteria-initiated click chemistry showing (A) CuAAC reaction triggered by bacteria for POC (point-of-care) microbiological identification; (B)–(D) representations and DFM images within the microreactor for three scenarios: (B) absence of bacteria, (C) presence of bacteria, and (D) presence of both bacteria and antibiotics^144^ (adapted/reproduced from ref. 144 with permission from Elsevier, copyright 2025).

#### DNA structure-based signal amplification

DNA structure-based techniques create amplified signals by taking advantage of ‘NA's programmable self-assembly and molecular recognition potential.^145^ A single target-recognition event can be converted into several signal-generating steps by a variety of DNA architectures, such as DNA walkers, DNA tetrahedra, and DNA nanomachines.^145,146^ Click chemistry's integration of 1,2,3-triazole linkages makes it easier to synthesise and functionalize these DNA nanostructures into sensing interfaces, enabling them to be used in sensitive biosensing platforms for a variety of analytical applications.^146,147^

Zhu and co-workers designed a nucleic acid identification method with a limit of detection of 8.1 × 10^−11^ µM using sensing technology. The researchers used an o-(propargyl)-*n*-(triethoxysilylpropyl) functionalized glass slide hybridised with cDNA 1 and cDNA 2 linked by the 1,2,3-triazole moiety. Subsequently, hairpin DNA duplex and swing-strand DNA were conjugated onto the glass slide DNA, which was further exposed to the target DNA, and the DNA walker was activated. The activated DNA walker cleaved the hDNA in the presence of Mn^2+^ cofactors, resulting in the release of the fluorophore, which amplified the signal and appeared as spots under a confocal microscope that were further digitized into pixels.^148^

Wang and co-authors fabricated a copper detection technique based on a copper(i)-catalysed click reaction-triggered 3D DNA walker. The sensing mechanism initiated with the click reaction of the azido group of AuNPs with an alkyne-modified H_2_C_2_-swing arm in the presence of target copper(ii) ions. Under N.BstNBI (nicking endonuclease *B. stearothermophilus* NBI) reaction, the 1,2,3-triazole-linked H_2_C_2_-swing arm cleaved the Cy3-labeled DNA fragments and freed the Cy3 fluorophore, and the fluorescence signal was amplified (Fig. 17).^149^

**Fig. 17:**
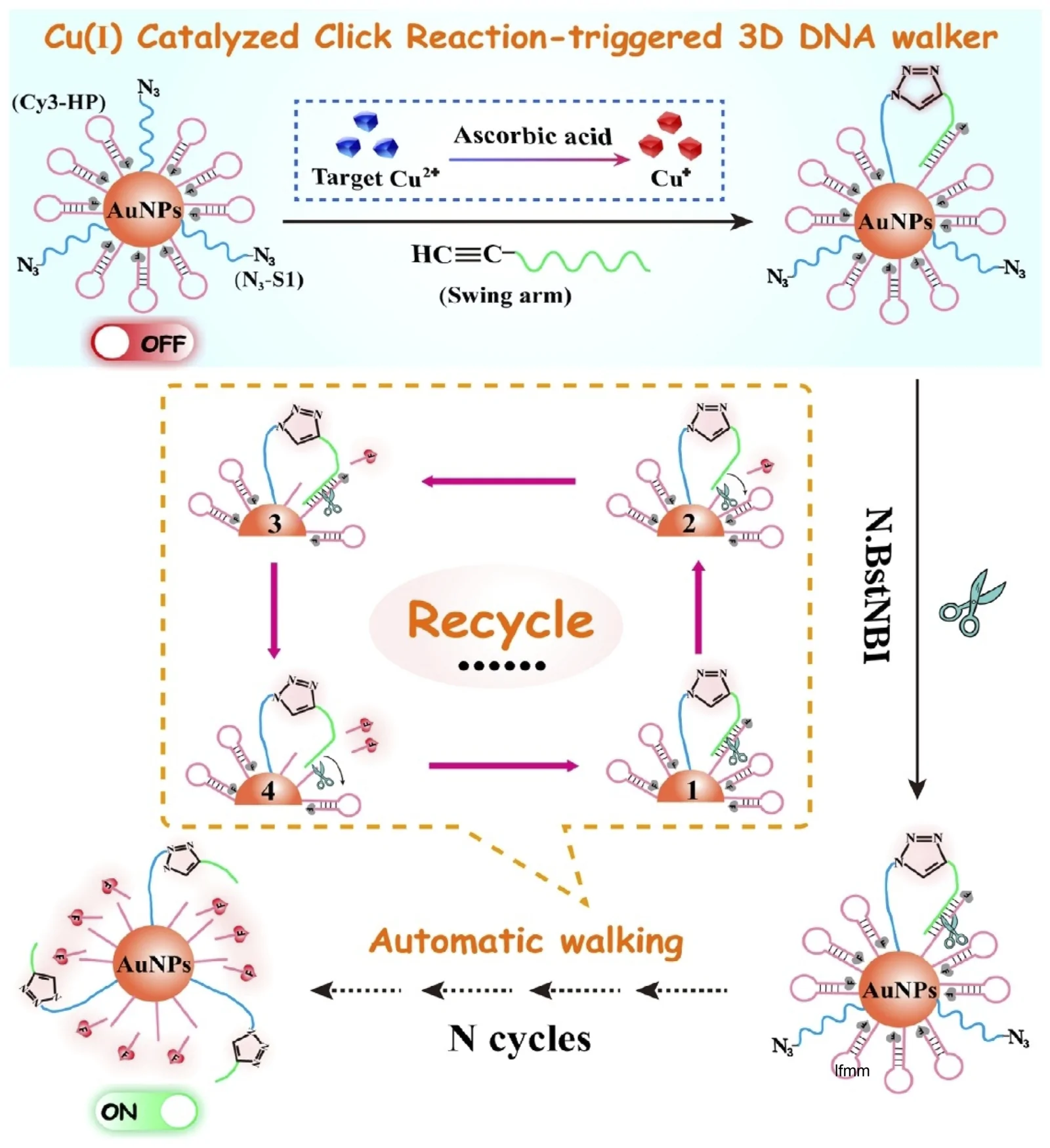
Diagrammatic representation of the Cu(i)-catalyzed to build the “OFF−ON” fluorescent biosensor for intracellular copper(ii) ions detection, click reaction-triggered 3D DNA walker^149^ (adapted/reproduced from ref. 149 with permission from the American Chemical Society, copyright 2021).

Qing *et al.* developed a 3D DNA walking machine based on click chemistry for copper(ii) ion detection. The working principle of the biosensor is centered on polystyrene microsphere@gold nanoparticle (PSC@Au) functionalized alkynyl-labeled S1 (alkynyl-S1), and hairpin-locked DNAzyme. The presence of copper(ii) ions triggered the click reaction between alkynyl-S1 and azido-DNA to form 1,2,3-triazole and generate the walker probe on PSC@Au. Further, the walker probe caused an unfolding of the hairpin-structured DNAzyme and, in the presence of magnesium ions (Mg^2+^), the DNAzyme cleaved the self-strand at the ribonucleotide site and released DNA S3. With the aid of the 3D DNA walking machine strategy, many S3 fragments are generated and initiate CHA recycling, which further amplified the biosensor signal. Recovery rates ranging from 98.8% to 105% and a low LOD of 3.3 × 10^−7^ µM validate the usage of the biosensor for clinical diagnosis and in environmental areas.^150^

Fang Yang *et al.* engineered an ECL biosensor for glutathione (GSH) detection with the aid of GSH-induced click chemistry and self-assembled tetrahedral DNA blocks. The detection technique was facilitated by the synthesis of self-assembled tetrahedral DNA blocks TDB_1_ and TDB_2_ by utilization of L_1_, M, S and L_2_, M, S ssDNA sequences, respectively; however, M and S sequences were alkyne-modified. A AgNP- and c-DNA-immobilized glassy carbon electrode was conjugated with 3D matrix TDB_1_ and TDB_2_. Subsequently, copper(ii) reacted with target GSH and formed copper(i)-GSH, which triggered the CuAAC. Therefore, the azide-functionalized AuAG NCs linked to a 3D matrix through 1,2,3-triazole linkage *via* click reaction, which amplified the signal. The biosensor performance was analysed in human serum samples and yielded recoveries ranging from 98.5–101.9%.^151^

### Applications

The studies and mechanisms discussed earlier, including signal probe polymerization, G-quadruplex formation, signal probe modification, DNAzyme-catalysed processes, DNA linkage, and integration with CRISPR/Cas systems, *etc.*, improved DNA biosensing in many ways, such as sensitivity, selectivity, stability, and signal amplification. Through the efficient use of CuAAC, biosensors have progressed from laboratory experiments to displaying encouraging effectiveness in analyses conducted in real-world settings.

As a result, click chemistry-based biosensors have been successfully utilized for the detection of a wide variety of analytes. The adaptability of the 1,2,3-triazole linkage allows for the construction of very sensitive and selective sensing platforms that can operate in complex matrices. Numerous documented biosensors have shown outstanding analytical performance in actual samples, such as biological fluids, food items, and environmental samples, underscoring their usefulness. The main applications of CuAAC-assisted DNA biosensors are outlined in the following sections, which also show how click chemistry plays various roles in practical target detection in biomedical, diagnostic, food and environmental monitoring.

Click chemistry-based biosensing platforms and portable analytical equipment have proven effective for detection analysis due to their better performance. This section explores the applications of click-assisted DNA biosensors in biomedical, diagnostic, food and environmental monitoring.

#### Genetic biomarkers

In recent biomedical research, genetic biomarkers, such as specific DNA sequences, miRNAs, and single nucleotide polymorphisms, have evolved to detect many possible diseases or used to identify biological conditions. Thus, the use of DNA and miRNA as biomarkers to monitor normal biological processes, disease-associated changes, or responses to therapy is promising.^152–154^ Early diagnosis and tailored medication depend on the sensitive and specific identification of genetic biomarkers. DNA biosensors have become effective analytical tools for the identification of genetic biomarkers because of their innate ability to recognize nucleic acids. This subtopic of the review explores the use of 1,2,3-triazole-assisted biosensors for sensitive and selective detection of DNA and miRNA biomarkers. Representative click-chemistry-based DNA biosensors and their quantitative analytical performance for biomarkers are compared in Table 1.

**Table 1:** Comparison of the representative quantitative analytical performances of click-chemistry-based DNA biosensors for biomarker detection

Target	Detection method	ROD (µM)	LOD (µM)	Real-life samples	Recovery rate	Ref.
**Detection of DNA biomarkers**						
p53	Electrochemical	1 × 10^−3^ to 128 × 10^−3^	0.35 × 10^−3^	Fetal bovine serum	91.0% to 118.0%	157
DNA	Electrochemical	1 × 10^−11^ to 1 × 10^−5^	1.954 × 10^−12^	NHS		132
DNA	Electrochemical	1 × 10^−13^ to 1 × 10^−8^	0.2 × 10^−12^	Spiked human serum	86.4% to 97.5%	133
DNA	Electrochemical		1.3 × 10^−4^			159
DNA/RNA	Electrochemical	1 × 10^−5^ to 3 × 10^−3^	2 × 10^−4^	Blood serum		161
DNA	Fluorescence	5 × 10^−12^ up to 1 × 10^−15^	2.8 × 10^−12^	20% fetal bovine serum and 5% human HepG2 cell lysate samples	94.8% to 98.7% and 85.1% to 91.6%, respectively	102
p53 gene	SERS	1 × 10^−8^ to 1 × 10^−5^	1.74 × 10^−8^	Human serum sample	91.70% to 112.84%	87
Single-nucleotide polymorphisms	Calorimetric	1.0 × 10^−4^ to 0.01				158
DNA	Fluorescence	1 × 10^−10^ to 1	4.3 × 10^−9^	Fetal bovine serum	97.6% to 93.2%	188
5 mC DNA	Electrochemical	10^−6^ to 10^−3^	1.2 × 10^−10^	Serum samples	95.60% to 103.94%	52

**Detection of miRNA**						
miRNA	Electrochemiluminescence	1 × 10^−8^ to 0.01	1.0 × 10^−8^	A549 cells		164
miRNA-21	Electrochemiluminescence	1.0 × 10^−9^ to 1.0 × 10^−3^	2.6 × 10^−10^	Real serum sample	90% to 108%	94
miRNA	Electrochemical and SERS	1 × 10^−10^ to 1 × 10^−3^	1.7 × 10^−10^ in SERS mode, 3.2 × 10^−11^ in ECL mode	1% saliva, 10% urine, 10% cell lysis buffer, and 10% serum samples		86
miRNA-141	Electrochemical	1.0 × 10^−7^ to 0.1	7.78 × 10^−9^			81
miRNA-18a detection	Electrochemical	1.0 × 10^−9^ to 5 × 10^−5^	2.5 × 10^−12^	10% normal human serum	92.5% to 94.5%	130
MicroRNAs	Electrochemical	1.0 × 10^−13^ to 5 × 10^−5^	2.5 × 10^−12^	Human serum samples		189
microRNA-21	Electrochemical	1.0 × 10^−11^ to 1.0 × 10^−5^	2.81 × 10^−12^	Human serum sample	92.2% to 96.8%	190
mRNA	Fluorescence		1.7 × 10^−6^	MCF-7 cells		166
Nucleic acids	Electrochemical	1.0 × 10^−15^ to 1.0 × 10^−8^	4 × 10^−13^	Fetal bovine serum (20%) and human HepG2 cell lysate (5%) samples	90.2% to 96.6% and 91.7% to 100.4%	165
*mecA*	Electrochemical	1.0 × 10^−10^ to 1.0 × 10^−8^	6.0 × 10^−11^	Lake water and milk	87.82% to 118.78%	140
miRNA-21	Electrochemical	5 × 10^−10^ to 0.001	1.068 × 10^−13^	Real human breast cancer cells		60
miRNA-21	Electrochemical	1 × 10^−7^ to 1 × 10^−1^	7.2 × 10^−11^	Human serum sample	90% to 103%	84

**Detection of enzyme**						
Thrombin	Electrochemical	0.8 nM to 40 nM	0.22 nM	Fetal bovine serum	91.0% to 118.0%	157
Thrombin	Fluorescence	50–1000 ng mL^−1^	28.46 ng mL^−1^	Human serum	93.53–104%	172
Pyrophosphatase activity	Fluorescence	0.05 to 25 mU	0.02 mU			169
Alkaline phosphatase	ECL	0.002 to 50 U L^−1^	0.7 mU L^−1^	Human serum samples	95.3% to 103.6%	92
Alkaline phosphatase	Electrochemical	10^−8^ to 10^−2^ U L^−1^	9.5 × 10^−7^ U L^−1^	Human serum samples	95.06% to 106.2%	141
Alkaline phosphatase	Fluorescence	0.1 to 40 U mL^−1^	0.05 U mL^−1^	Serum samples		170
Pyrophosphatase	Electrochemiluminescence	0.025–50 mU	8 mU	Serum from four clinical arthritic patients	96% to 105%	93
DNA methyltransferase	Electrochemiluminescence	0.1 to 20 U mL^−1^	0.03 U mL^−1^	Diluted human serum	94.6% to 106.3%	173
Alkaline phosphatase	Fluorescence	0.1–5 U L^−1^	0.079 U L^−1^	5–70% diluted human serum	43.9% to 95.5%	171
DNA methyltransferase activity	Fluorescence	0.0025 to 10 U mL^−1^	0.001 U mL^−1^	Human serum samples	96.9% to 103.9%	174

**Detection of proteins**						
VEGF_165_	Electrochemical	0.1 nM to 5.0 nM	0.014 nM	Fetal bovine serum	91.0% to 118.0%	157
*Trichinella spiralis* crude protein	Fluorescence	3.125 to 100 ng mL^−1^	0.35 ng mL^−1^	Pork samples	92.61% to 102.28%	114
Interferon gamma (IFN-γ)	Fluorescence	0.01 pM to 10 nM	1.75 fM (1.63 fM in serum)	Fetal bovine serum		65
VEG_165_	Electrochemical	0 to 10 µM	6.2 nM			179
DR1	Electrochemical	5 × 10^−4^ ng mL^−1^–5 × 10^2^ ng mL^−1^	0.159 pg mL^−1^	Clinical sample		191
Antibody detection	Electrochemical	5 pM to 200 nM	1.5 pM	Human serum samples	93.6% to 107.3%	180

**Detection of tumors**						
Tumor	Electrochemical	1.12 × 10^2^ to 1.12 × 10^8^ particles per µL	96 particles per µL	Human serum samples from cancer patient		183
Cancer biomarkers	Electrochemical	0 to 1.0 nm	1 fm	Human blood	100%	184
Carbohydrate antigen 24-2	Electrochemical	0.0001 to 100 U mL^−1^	20.74 µUmL^−1^	Five human serum samples		185
Circulating tumor cells	LF-NMR	1.0 to 1.0 × 10^6^ cells per mL	5 cells per mL	Human whole blood samples	97.7% to 104.6%	187
Breast cancer-derived exosomes	Fluorescence	6.50 × 10^7^ to 1.30 × 10^9^ particles per mL	6.09 × 10^7^ particles per mL	Human serum samples		186
CA72-4	Electrochemical	10 to 1000 mL^−1^	7.11 mL^−1^	Clinical blood samples		83
Exosomes	Electrochemical	5 × 10^3^ to 5 × 10^9^ particles per mL	1.49 × 10^2^ particles per mL	Human serum samples		62
Extracellular vesicles	Electrochemical	10^3^ to 10^9^ particles per mL	625 particles per mL	Human serum samples	98.2% to 109.1%	51

#### DNA detection

DNA detection is used in medicine for disease diagnosis, gene expression profiling, and precision medicine. Probe and target hybridization has been the traditional method for DNA detection, but this has drawbacks such as low sensitivity and poor probe attachment. Modern scientists are developing methods to address these drawbacks, such as enhanced signal amplification, probe immobilization, and real-time analysis; these have facilitated the development of robust sensing platforms with excellent sensitivity, selectivity, stability, and user-friendly operation for precise biomarker identification.^155,156^ Click chemistry is an efficient and stable approach for the detection of genetic biomarkers, with 1,2,3-triazole linkage offering a stable linkage for probe attachments.

Fan *et al.* developed a disposable multiplexed electrochemical sensor employing alkyne–azide cycloaddition for the simultaneous detection of DNA, enzymes and proteins. In the biosensor, Au-SPCE-N_3_ (azide-modified gold-plated screen-printed carbon electrode) underwent click reactions with an alkyne-functionalized oligonucleotide protein 53 probe, thrombin aptamer, and Vascular Endothelial Growth Factor (VEGF) aptamer to individually form 1,2,3-triazole linkages. The DNA probes were immobilised on screen-printed carbon electrodes for the simultaneous detection of p53 DNA, thrombin, and VEGF_165_. The sensor exhibited detection limits of 0.35 × 10^−3^ µM for the p53 DNA target, 1.4 × 10^−4^ µM for VEGF_165_ and 2.2 × 10^−4^ µM for thrombin protein. Three samples were evaluated in 50% fetal bovine serum, yielding recovery rates ranging from 91.0% to 118.0%, demonstrating its potential for clinical and medical diagnostics.^157^

Zhang and co-workers fabricated a biosensor that depended on cation-exchange of CuS nanoparticles and click chemistry for the detection of single-nucleotide polymorphisms and DNA methyltransferase (MTase). The mechanism of the biosensor involved streptavidin-modified magnetic beads (MB) functionalized with biotin-modified capture DNA, which underwent conjugation with target DNA and reporter DNA-1 modified with CuS nanoparticles to form a sandwich structure. When nuclease S1 was exposed, intact wild-type DNA was preserved while the mismatch site of the mutant DNA was subjected to digestion, which led to release of CuS nanoparticles. AgNO_3_ was used to free copper(ii) *via* a cation-exchange reaction and, in the presence of sodium ascorbate and TBTA, the click reaction functionalized the AuNPs with the 1,2,3-triazole linkage. The change in the solution colour from red to blue was analysed *via* UV/vis spectroscopy. For DNA methyltransferase (MTase) detection, a hairpin probe modified with 5′-G-A-T-C-3′ was methylated in the presence of Dam MTase. Subsequently, the methylated probe was cleaved with the aid of the endonuclease Dpn I and further immobilized on MB. The CuS NP functionalized reporter DNA 2 was conjugated to cleave the DNA duplex.^158^

Galán *et al.* synthesised an electrochemical DNA sensor with a limit of detection of 1.2 × 10^−4^ µM based on a poly(3,4-ethylenedioxythiophene) (PEDOT) electrode. The biosensor was utilised to identify specific DNA sequences and was capable of diagnostic uses in the detection of genetic abnormalities and illnesses such as hepatitis C. In the presence of copper(i), azido-functionalized poly(3,4-ethylenedioxythiophene) (PEDOT) electrodes conjugated to an acetylene-terminated oligonucleotide probe through 1,2,3-triazole *via* the click reaction, which resulted in DNA-functionalised PEDOT. Combined DNA was recorded using differential pulse voltammetry (DPV).^159^

Wiarachai *et al.* designed a biosensing technique that employed a clickable and antifouling polymer coating on a gold surface. The analytical method involved two click reactions in two different targets: DNA and streptavidin. The coated gold electrode underwent click reactions with azide-containing biotin and peptide nucleic acid in the presence of copper(i), leading to a CuAAC reaction that resulted in 1,2,3-triazole-linked ring derivatives containing biotin and peptide nucleic acid, which subsequently conjugated to target streptavidin and DNA, respectively.^160^

Komkova *et al.* constructed an electrochemical DNA/RNA sensor employing catalytically produced Prussian blue nanoparticles functionalized with azidomethyl-substituted poly(3,4-ethylenedioxythiophene) (azidomethyl-PEDOT). The detection process involves azide-functionalized Prussian blue nanozymes combined with an alkyne-modified DNA probe *via* 1,2,3-triazole, forming azidomethyl-PEDOT films. Another click conjugation occurred between an azide-functionalized graphite carbon electrode and an alkyne-capture DNA probe *via* 1,2,3-triazole. The azidomethyl-PEDOT films were hybridised onto the graphite carbon electrode. Both 1,2,3-triazole linkages resulted in a sandwich-type sensor. Upon introduction of H_2_O_2_, PB nanozymes catalysed the reduction of H_2_O_2_, and the electrocatalytic current of H_2_O_2_ reduction was measured, which indicated both high selectivity and sensitivity. The observed range of detection was 0.01 nM–10 nM, and the limit of detection was 2 × 10^−4^ µM in human blood serum samples, demonstrating the compatibility of the biosensor for DNA/RNA detection and point-of-care sensing.^161^

#### MicroRNA detection

Many human diseases, including metabolic disorders, neurological diseases, cardiovascular diseases and cancers, have been directly correlated with miRNA expression, especially miRNA-21, which acts as a key biomarker for cancer diagnosis. Slight changes in their concentration can demonstrate the onset of a disease; however, facile detection is quite difficult due to their micro size, similar sequences and low concentrations in biological samples. The conventional methods are limited due to a lack of sensitivity and limited capacity to discriminate between miRNA sequences.^162,163^ Modern scientists prefer to use signal amplification and probe engineering techniques for better results. Thus, click chemistry provides strong and specific probe linkage and signal amplification through the 1,2,3-triazole group, facilitating accurate, ultrasensitive, and real-life detection.

Lu and co-authors developed an ECL biosensor for the detection of miRNA let-7d using a [Ru(bpy)_3_]^2+^ labelled DNA probe, with a range of detection of 1 × 10^−8^ to 0.01 µM. In this sensor, a GCE was modified with graphene oxide and coated with a mixture of 1-pyrenebutyric acid *N*-hydroxysuccinimide (PASE) and methylene blue (MB). 3-Azido-1-propylamine (3-azido-1-PrA) was covalently linked to the 1-pyrenebutyric acid *N*-hydroxysuccinimide and underwent a click reaction with [Ru(bpy)_3_]^2+^ labelled alkynyl DNA hairpin probe(alkynyl-DNA-Ru) to form a 1,2,3-triazole linkage. In the presence of the anodic ECL co-reactant BDEA, the modified electrode exhibited very weak ECL emission. However, upon hybridization of the hairpin probe with the target miRNA, a significant increase in ECL was observed. This biosensor was successfully applied for the detection and quantification of miRNA let-7d in A549 cells, highlighting its potential for sensitive miRNA analysis in biological samples.^164^

Wang and colleagues designed an ultrasensitive detection method for biosensing nucleic acids with a mechanism involving azide-functionalized P1 and alkyne-functionalized P2 DNA probes. In the presence of the heterogeneous Cu_2_O nanocatalyst, CuAAC click ligation occurred, which initiated the first round of hnCu_2_O-DT-CLCR and formed the ligated P1–P2 strand as a P1–P2/TD duplex. The target DNA took part in additional cycles when the duplex dissociated after thermal denaturation, releasing free TD and the ligated P1–P2 product. The azide-functionalized H1 and alkyne-functionalized H2 subsequently hybridized onto the newly created P1–P2 strand, which served as a secondary template. The P1–P2/H1–H2 duplex is created when a second click ligation process is catalyzed by hnCu_2_O, which served as a template for further P1 and P2 ligation following denaturation. The target DNA signal is amplified exponentially without the need for enzymes as a result of the reciprocal cross-templating between P1–P2 and H1–H2 over multiple heat cycles. The recovery rates in diluted fetal bovine serum (20%) and human HepG2 cell lysate (5%) samples for both signal methods were 90.2–96.6% and 91.7–100.4% in the E-DNA sensor and the colourimetric DNA sensor, respectively (Fig. 18).^165,166^ Fan Xiao *et al.* developed a biosensing technique for miRNA detection with a LOD of 1.7 × 10^−6^ µM, based on a click reaction for DNA conjugation on different sites of PFBT (polyfluorene-*alt*-benzothiadiazole). The product spontaneously self-assembled into spherical nucleic acid structures, which were further used for *in situ* detection of miRNA and imaging at the single-cell level.^167^

**Fig. 18:**
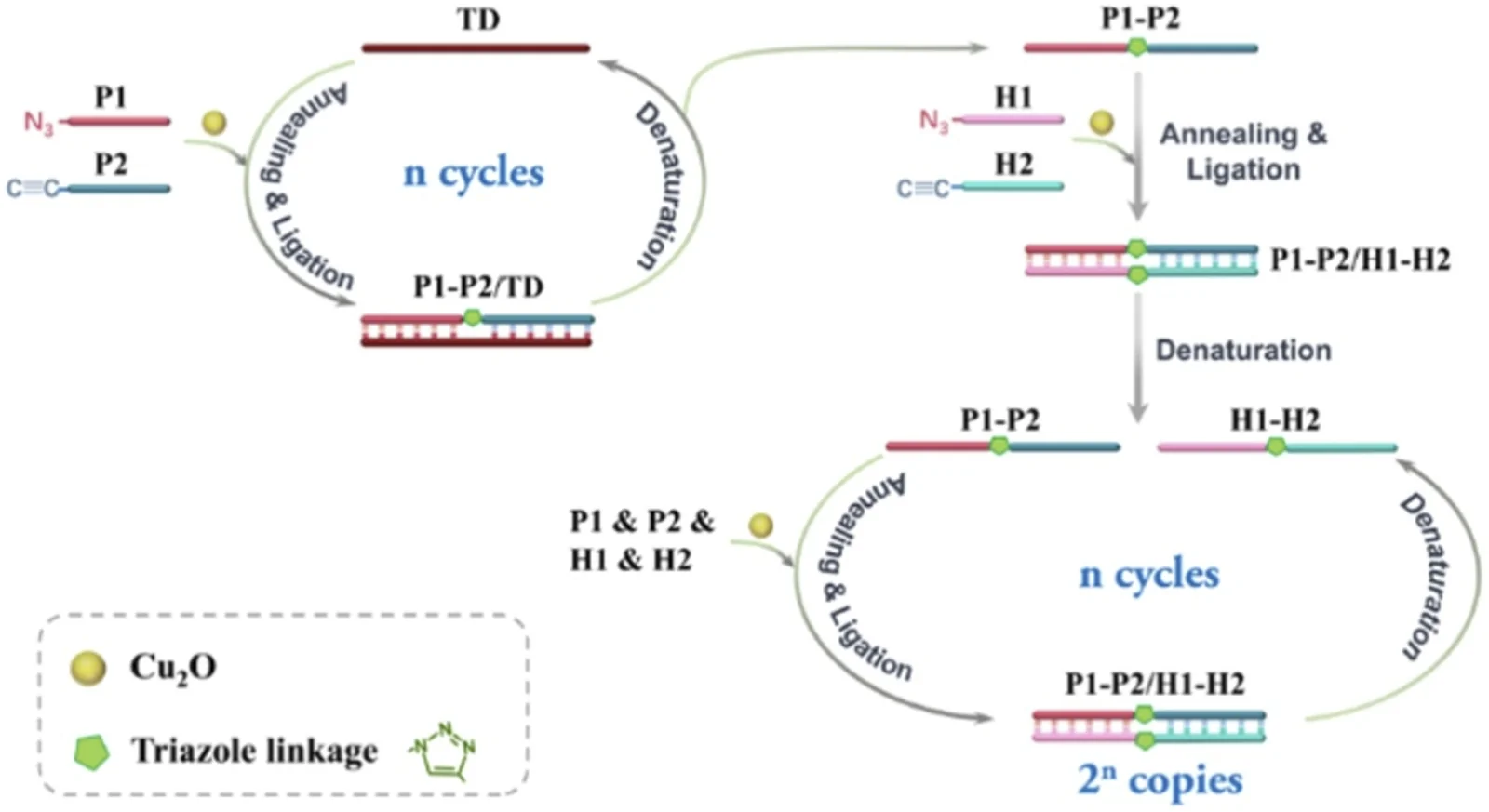
Schematic of the DNA-templated click ligation chain reaction catalyzed by hnCu_2_O and exponential DNA amplification by hnCu_2_O-DT-CLCR^165^ (adapted/reproduced from ref. 165 with permission from the American Chemical Society, copyright 2024).

#### Enzyme biomarkers

Enzymes play a crucial role in nearly all biological systems due to their exceptional substrate selectivity, rapid reaction rates, and efficient catalytic activity. Many enzymes are particularly important in biomedical and clinical diagnostic research. Their clinical relevance stems from their use as enzymatic biomarkers and indicators of pathological states. For example, ALP is associated with various diseases, such as Alzheimer's disease and cirrhosis.^167,168^ Click reaction-assisted enzyme detection provides high sensitivity, stable probes, and excellent performance in real samples.

Zhang and co-authors developed a fluorescence biosensor with an ROD ranging from 0.05 to 25 mU and a detection limit of 0.02 mU for pyrophosphatase ion (PPi) activity detection. The mechanism underlying this biosensor entails a PPi solution that forms a stable PPi/Cu^2+^ complex [Cu(P_2_O_7_)_2_^6−^], which does not favour the redox conversion of copper(ii) ions. Upon introduction of PPi, hydrolysis of pyrophosphatase released Cu(ii) ions and reduced them to Cu(i) with sodium ascorbate, which catalyses the click reaction of a 5′-azide-tagged padlock probe and 3′-alkyne, forming 1,2,3-triazole. The reaction further underwent a hyperbranched rolling circle amplification reaction to generate chain extensions and strand displacements with DNA polymerase, primer 1, primer 2, and dNTPs (deoxynucleotides), thus generating a fluorescence signal upon the addition of SYBR Green I to the system.^169^

Yang and co-authors designed a biosensing platform for alkaline phosphatase quantification in serum samples, an analytical method that utilizes hydrolysis of ascorbic acid-phosphate to active ascorbic acid, which reduced copper(ii) to copper(i) to catalyse CuAAC. The 1,2,3-triazole linkage formed *via* click reaction between alkyne-poly(thymine) segments and azide-poly(thymine) segments generated a longer poly(thymine) sequence. The reduced copper(i) was utilized to synthesise CuNPs in the presence of ascorbic acid and a DNA template (longer poly (thymine)). CuNPs formation was analysed, and the fluorescence peak was used to quantify the ALP. The biosensor enabled real-time analysis of ALP in human serum, which validates its potential use in complex biological matrices.^170^

Yu *et al.* synthesised a fluorescence platform for alkaline phosphatase by employing reversible addition fragmentation chain transfer (RAFT) polymerization. The sensing was initiated through a click reaction catalysed after hydrolysis of the complex of [Cu(P_2_O_7_)_2_]^6−^*via* ALP. The azide, monoethanolamine-functionalized magnetic beads and propargyl-PEG1-acid formed 1,2,3-triazole, which led to a further chain transfer agent integrated with Zr(iv) *via* the carboxylic group of monoethanolamine. RAFT polymerization was achieved using 2,2′-azobis(2-methylpropionitrile) (AIBN) as the initiator and 1-(4-vinylphenyl)-1,2,2-triphenylethylene as the polymerization monomer in an organic solvent for a better fluorescence signal. Different human serum concentrate samples were analysed, and recovery rates were obtained that ranged from 43.9–95.5%, with an LOD of 0.079 U L^−1^ (Fig. 19).^171^

**Fig. 19:**
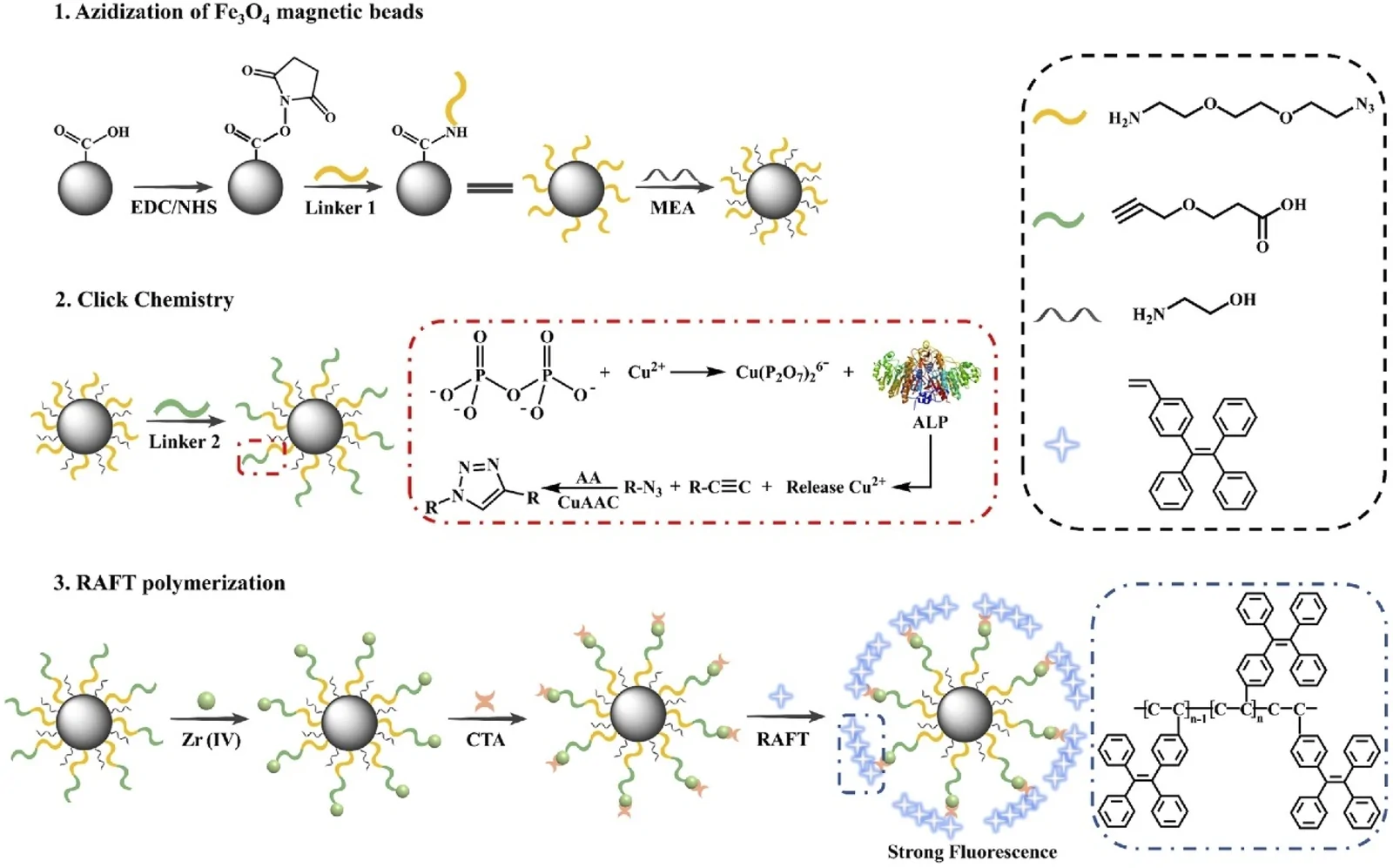
Mechanism of controlled chain growth luminescence biosensor based on RAFT polymerization demonstrating (1) azidization of Fe_3_O_4_ magnetic beads (2) click reaction using copper (3) RAFT polymerization^171^ (adapted/reproduced from ref. 171 with permission from Springer, copyright 2024).

Chen *et al.* designed a fluorescence biosensor based on click chemistry for thrombin detection, with a detection range of 50–1000 ng mL^−1^ and a detection limit of 28.46 ng mL^−1^. It utilises mesoporous silica nanoparticles (MSN) functionalized with –N_3_, which undergo a click reaction to give 1,2,3-triazole with double-stranded DNA containing a thrombin aptamer that caps the pores of MSN. Thrombin binds strongly to the aptamer, causing the aptamer to detach from MSN. The single-strand DNA (ssDNA) was unable to fully block the MSN pore, and the fluorescein was released into the solution through the MSN pores. A healthy human serum sample was analysed, and the biosensor showed a good recovery rate and high selectivity.^172^

Zhao *et al.* constructed an electrochemiluminescence biosensor for DNA methyltransferase activity. The analytical sensing technology involved an electrode surface functionalized with target DNA, which was subsequently modified with azide-functionalized DNA and underwent CuAAC conjugation with alkynyl-functionalized GO-AgNPs-luminol composites to form the 1,2,3-triazole linkage. On exposure, the DNA adenine methylation (Dam) methyltransferase (MTase) signal was reduced, enabling the qualification of the target. The biosensor exhibited recovery rates for the activity of Dam MTase of 94.6–106.3%.^173^

Cao *et al.* developed a fluorescence detection platform for methyltransferase activity in human serum samples with an LOD of 0.001 U mL^−1^ and recovery rates of 96.9–103.9%. The biosensor mechanism involved DNA adenine methyltransferase (Dam MTase), which methylated a specific adenine in the DNA-substrate-functionalized CuONPs. The methylated DNA is cleaved by Dpnl, thereby releasing CuO nanoparticles. These nanoparticles lead to a CuAAC reaction between the alkyne-functionalized DNA anchored on magnetic beads and DNA-azide, forming an intact DNA primer through a 1,2,3-triazole linkage. This framework enables the extensive production of gold nanoparticle nanozymes that replicate the function of glucose oxidase. These nanozymes aid in the oxidation of glucose, producing hydrogen peroxide. NH_2_-MIL-101 metal–organic framework nanozymes subsequently utilize this hydrogen peroxide to convert *o*-phenylenediamine into a fluorescent compound.^174^

#### Protein biomarkers

Proteins are a major component of living cells and exhibit numerous cellular activities; they can also provide information about diseases, due to the presence of biomarkers consisting of metabolic, structural, and regulatory functions in response to various diseases.^175^ In other words, disease progression can be regulated through the interactive functions of proteins. 1,2,3-Triazole-assisted biosensors detect protein molecules for clinical diagnosis and treatment.^176–178^

Feng and co-authors engineered an aptamer-based biosensor with a detection limit of 6.2 nM for VEGF_165_ detection. The sensor mechanism employs UDT-N_3_/UDT (undecanethiol and azido-1-undecanethiol) self-assembled monolayer-modified AuE hybridised with an aptamer terminal ferrocene alkyne moiety *via* 1,2,3-triazole. On addition of VEGF_165_ protein, its binding causes a conformational change in the aptamer, altering the terminal such that the ferrocene electron transport modified on the gold electrode produces measurable redox signals. After a 6 M urea solution wash, the sensor can be reused, yielding an 84% response rate, supporting its implementation in clinical diagnosis and treatment.^179^

Dou and co-authors engineered a detection platform for antibody detection at the picomolar level based on programmable DNA Nanomachine-eATRP. The biosensor used two recognition DNA probes (RP1 and RP2), each labelled with digoxigenin (Dig), bound to an anti-Dig antibody, bringing RP1 and RP2 into close proximity. In the presence of a substrate probe (SP) immobilised on magnetic beads, the generated antibody–DNA complex started a strand displacement reaction that released a blocking strand and revealed a new toehold region. The exposed toehold was then bound by a DNA probe modified with CuO nanoparticles (DP–CuO), creating a SP/DP–CuO combination and releasing the antibody–DNA complex for recycling. The amount of CuO nanoparticle-tagged DNA products on the magnetic beads is further increased by an auxiliary DNA strand (AMP) that imitated the antibody-induced proximity effect and initiated a second strand displacement cycle. Following magnetic separation, the CuO nanoparticles dissolved in an acidic solution to produce copper(ii) ions. These ions are then reduced to copper(i) and utilised to catalyze azide–alkyne click chemistry and form the 1,2,3-triazole ring, which attaches ATRP initiators to an azide-functionalized gold electrode. ATRP technology takes place as discussed before. The biosensor yielded recovery rates from 93.6% to 107.3% in human serum samples. The electrochemical biosensor offers a convenient, sensitive, and cost-effective tool with a low LOD of 1.5 pM (Fig. 20).^180^

**Fig. 20:**
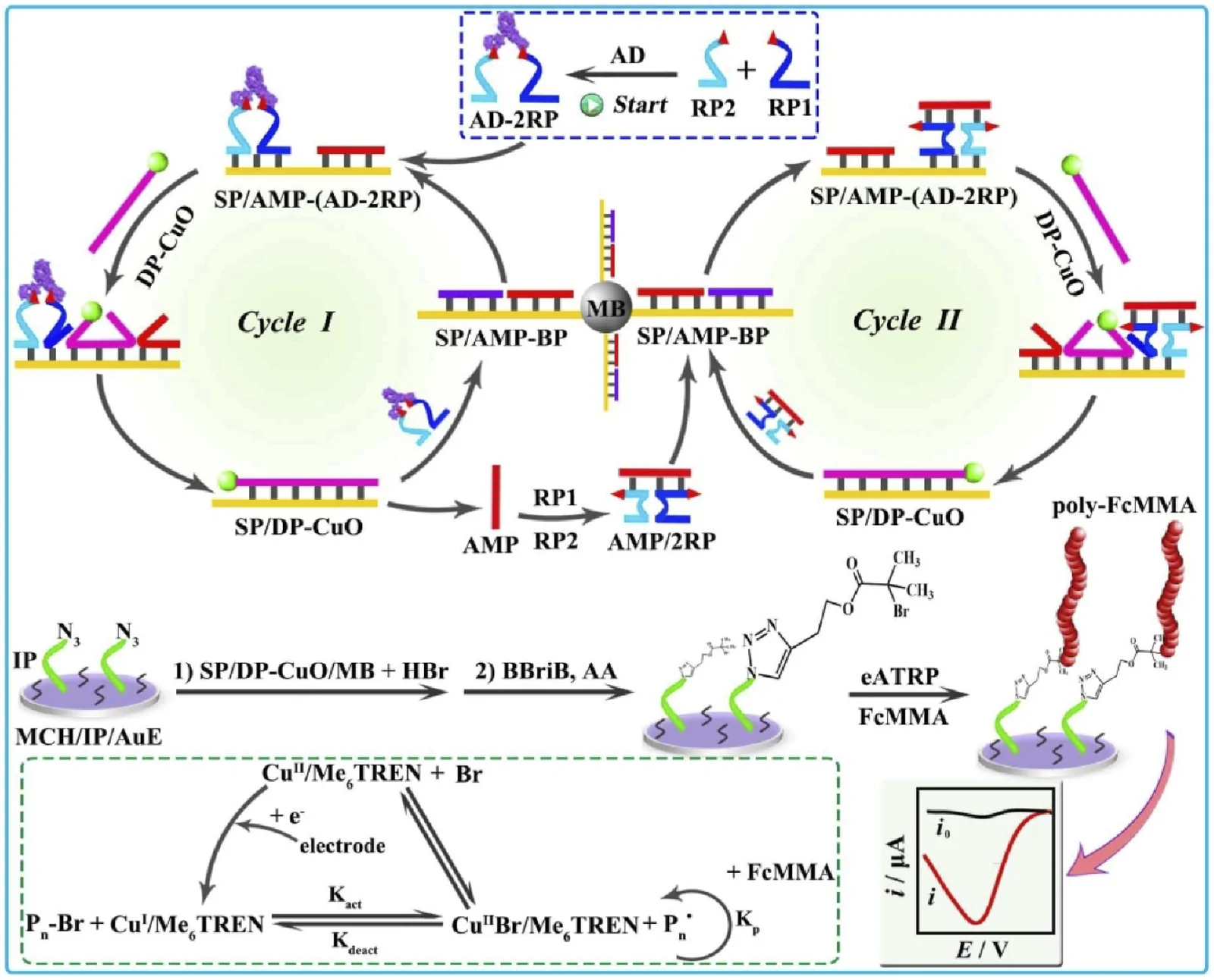
Programmable DNA nanomachine combining antibody-responsive eATRP amplification with cascading techniques^180^ (adapted/reproduced from ref. 180 with permission from the American Chemical Society, copyright 2024).

#### Tumor biomarkers

Malignant tumours continue to be one of the main illnesses that threaten lives and health and are an important factor in mortality. Early detection of tumors can enhance the rate of survival of patients. Traditional imaging can fail to detect tumors in the early stage,^181,182^ but CuAAC-based DNA biosensors have shown success in the detection of tumors.

An *et al.* engineered an aptasensor for the identification of tumor exosomes using copper(i)-catalysed click chemistry. The biosensor employed a glassy carbon electrode modified with a CD63 aptamer to capture exosomes. A 4-oxo-2-nonenal alkyne conjugate was attached to the exosome surface, which subsequently underwent a click reaction with an azide-labeled DNA probe, leading to the formation of a 1,2,3-triazole ring. A DNA hybridization chain reaction (HCR) involving biotin-H1, biotin-H2 and streptavidin-HRP led to the formation of long DNA concatamers. This HCR, in combination with the click reaction, played a crucial role in exosome linkage and signal amplification. The biosensor demonstrated a detection range of 1.12 × 10^2^ to 1.12 × 10^8^ particles per µL, with a limit of detection (LOD) of 96 particles per µL, indicating high sensitivity. It was found to be suitable for exosome detection in human serum and shows strong potential for applications in early cancer diagnosis, monitoring disease progression, and clinical sample analysis.^183^

A nanopore sensing technique relying on click chemistry was developed by Zhang and co-authors for the quantification of cancer biomarkers in human blood serum. The system was based on antibody-modified magnetic beads and CuO nanoparticles, which formed a sandwich complex structure with the target biomarker. Subsequently, in a hydrochloric acid environment, copper(ii) was released and reduced to copper(i) *via* ascorbate, followed by magnetic separation to isolate the released Cu(i). This catalytic copper(i) then facilitated a click reaction between alkyne-modified single-stranded DNA and azide-containing ferrocene–CB7 inclusion complex, resulting in the formation of a 1,2,3-triazole linkage. The hybridised DNA products were subsequently analysed through a single-channel using a nanopore sensor. This highly sensitive platform demonstrated a sub-femtomolar LOD, making it promising for point-of-care diagnostics and clinical identification of cancer biomarkers.^184^

Zheng *et al.* fabricated an electrochemical detection technique for carbohydrate antigen 24-2 monitoring in human serum. The sensing method involved PEI (polyethyleneimine) coated graphene oxide conjugated with the Ab_1_ capture antibody, which was further modified with propiolic acid to bind the extra binding sites on the electrode. The generated antibodies were conjugated to CuPDA (copper(ii) polydopamine) particles, which captured the CA 242. The CuPDA freed the copper(ii) in the acidic solution and reduced it to copper(i) to catalyse the CuAAC click reaction and subsequently introduce azido-modified dsDNA and propiolic acid along with 1,2,3-triazole. The electrostatic repulsion between the phosphate group of dsDNA and [Fe(CN)_6_]^3−/4−^ reduced the current signal, with satisfactory recovery rates.^185^

A fluorescence biosensor for breast cancer with an LOD of 6.09 × 10^7^ particles per mL of derived exosomes was developed by Ma *et al.* based on click chemistry. Click chemistry was initiated to accomplish exosome attachment through specific interactions between the lipid bilayer of exosomes containing phosphate groups and Fe_3_O_4_@TiO_2_ particles. Subsequently, alkyne-functionalized polymer dots and CD63-FAM-N_3_ were immobilized on the surface of the exosome, forming Fe_3_–O_4_@TiO_2_/exosome/CD63-N_3_*via* 1,2,3-triazole. 10% NH_3_·H_2_O was used to release the exosomes, which amplified the fluorescence signal due to the signal probe. The biosensor exhibited high sensitivity towards serum samples of both healthy individuals and breast cancer patients.^186^

Yang *et al.* designed a detection method based on target-triggered click chemistry for circulating tumor cells in human whole blood cells. The biosensor mechanism involved Fe_3_O_4_@Apt conjugation with MCF-7 and erythrocytes and leukocytes. Through magnetic separation, followed by hydrolysis, MCF-7 cells were released. Cystine-GO@CuO nanoprobes catalyzed thiazolidine bioorthogonal chemistry over the cell surface between the cysteine of the nanoprobe and aldehyde, and was then oxidised *via* NaIO_4_. On acid treatment, copper(ii) was released from the CuO and reduced to copper(i) *via* AA. A click reaction was facilitated between azide-PEG-azide and alkyne-PEG-alkyne. 1,2,3-Triazole formation in the sol–gel phase resulted in restricted motion of water molecules, which generated NMR signals. Accelerating transverse relaxation of water molecules was measured by LF-NMR. The biosensor exhibited recovery rates of 97.7–103.6%, along with a detection limit of 5 cells per mL (Fig. 21).^187^

**Fig. 21:**
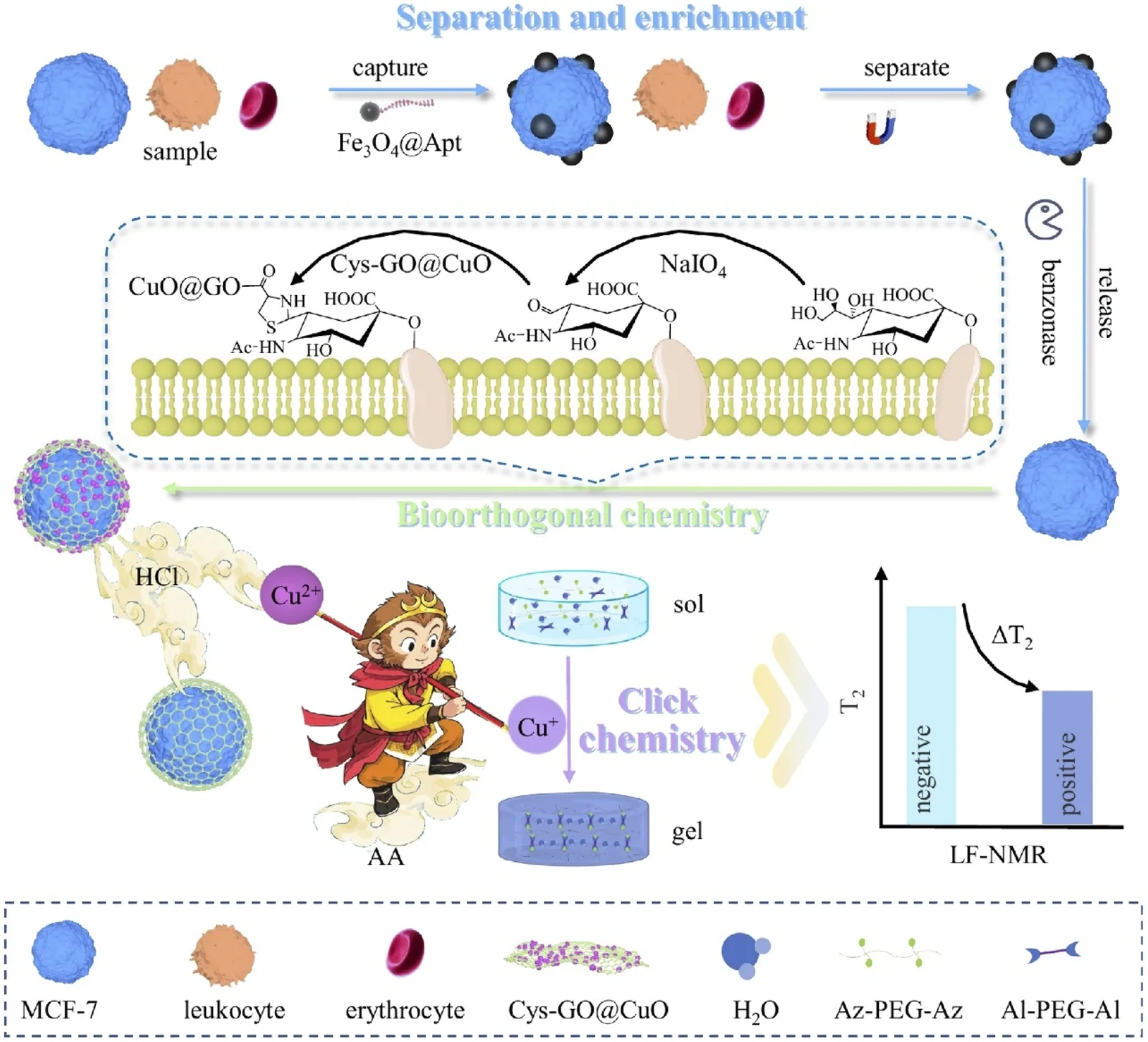
Detection principle for the proposed click chemistry-based LF-NMR biosensor^187^ (adapted/reproduced from ref. 187 with permission from the American Chemical Society, copyright 2025).

### Detection of pathogenic microorganisms

The majority of bacteria are harmless and exhibit a number of functions beneficial to humans and animals and the environment; however, it is estimated that less than 1% of bacteria cause diseases, and these have a large impact on human health. According to statistics, the number of deaths caused by immune deficiency diseases is less than the death toll caused by *S. aureus* infections. Therefore, bacterial detection and monitoring are the key factors for the diagnosis and treatment of bacterial infectious diseases.^192,193^ This section of the review explores the rapid and accurate detection of bacteria in real samples by 1,2,3-triazole-aided DNA biosensors. Representative click chemistry-based DNA biosensors and their quantitative analytical performance for the detection of pathogenic microorganisms are compared in Table 2.

**Table 2:** Representative quantitative analytical performance of click-chemistry-based DNA biosensors for microbial detection

Target	Detection method	ROD (CFU mL^−1^)	Detection limit (CFU mL^−1^)	Real-life samples	Recovery rate	Ref.
**Detection of bacteria**						
Copper(ii)-reducing bacteria	DFM	10^1^ to 10^7^ cells	10^1^ cells	Human plasma		144
*E. coli*	Fluorescence	10^−2^ to 10	0.003	Urine and blood samples		203
*S. aureus* and *E. coli*	Electrochemical	10^2^ to 10^7^	4 and 6			131
Urinary pathogens	Colorimetric	Not reported	119.1	Urine samples		204
*S. aureus*	Microfluidic chips	10 to 2.5 × 10^4^	3	Milk, pork, and fish samples	90.0% to 105%	120
*V. parahaemolyticus*	Low-field nuclear magnetic resonance	10–1.0 × 10^8^	5	Seawater samples and oyster samples	96.6% to 106.4% and 97.6% to 105.3%	195
Live *S. typhimurium*	Bioluminescence	20 to 10^5^	8	Many aquatic samples	90.9% to 113%	197
*S. aureus*	Fluorescence		93 and 270	Deionised water and spiked water samples	69.4% to 97.1%	142
*Salmonella*	Fluorescence	6 × 10^1^ to 6 × 10^7^	1	Milk, infant formula, orange juice, and meat samples	93% to 104%	117
*E. coli* O157:H7	Fluorescence	10^2^ to 10^8^	50	Pork and milk	91% to 110%	80
*S. aureus*	Transverse relaxation time	10^2^ to 10^6^	16	Eggs, milk and pork	75% to 112%	115
VP, ST, and SA	Fluorescence	1 × 10^2^ to 1 × 10^8^, 1 × 10^1^ to 1 × 10^8^, and 1 × 10^1^ to 10^8^	28, 10, and 9	Actual samples, different water samples and mixed spiked samples	Above 90%	200
*S. typhimurium*	Electrochemical	10^1^ to 10^7^	10	Spiked milk samples	101% to 107%	107
*V. parahaemolyticus*	Electrochemical and fluorescent	10 to 10^8^	6	Shrimp, crab, and fish samples	94.0% to 106%	76
*V. parahaemolyticus*	Electrochemical	10 to 10^7^	4	Shrimp aquaculture water	97.0% to 106%	196
*S. aureus*	Colorimetric	10 to 10^6^	2.4	Pork and water samples	91.15% to 106.36%	199
*E. coli* and *H. paralvei*	Bioluminescence assay and microfluidic chip assay	10 to 2.0 × 10^7^ (*E. coli*) and 10 to 1.3 × 10^7^ (*H. paralvei*)	30 and 3	Fish samples	93.7% to 109% and 90.0% to 106%	198
*E. coli* and *S.T.*	Electrochemical and fluorescent	10 to 10^7^	5 and 8	Milk, pork, fish, and lettuce samples	95.0% to 106% and 90% to 110%	122
Methicillin-resistant *S. aureus*	Fluorescence	50 to 10^6^	18	Milk, pork, and egg samples	80% to 101%	116
*P. fluorescens*	Fluorescence	10^2^ to 10^7^	1	Milk samples	97.8% to 102.9%	77
*E. coli*	Electrochemical	1 to 10^8^		Real sea water and milk samples	94% to 110%	82

**Detection of virus**						
SARS-CoV-2	Fluorescence	2 × 10^−4^ to 0.02	5.0 × 10^−4^			207
SARS-CoV-2	Electrochemical	1 × 10^−4^ to 1	3.383 × 10^−5^	Real saliva samples	96.47% to 101.04%	208
Tobacco mosaic virus RNA	Fluorescence	1.0 × 10^−7^ to 0.01	1.14 × 10^−9^	Healthy *Rehmanniae radix* leaves	92.85% to 101.83%	136
Tobacco mosaic virus RNA	Electrochemical	1.0 × 10^−7^ to 0.01	3.5 × 10^−9^	Healthy *Rehmanniae* leaves	98.95% to 108.7%	209
Hantaan virus	Fluorescence		5.754 × 10^−17^	HTNV-infected serum	92.21% to 103.66%	78
HTLV-II	Electrochemical	10^−6^ to 10^−3^	1.71 × 10^−7^	Fetal bovine serum		63

#### Monitoring bacteria in food and the environment

The fact that the majority of bacteria are harmless and perform numerous functions for humans, animals, and the environment, but an estimation that less than 1% of bacteria cause diseases, has a significant impact on human health. According to statistics, the number of deaths caused by immune deficiency diseases is less than the death toll from *S. aureus* infections. Therefore, bacterial detection and monitoring are key factors for food safety.^192–194^

DNA and click-modified biosensors have been employed for the detection of pathogenic bacteria such as *E. coli*, *S. typhimurium*, and *S. aureus* with high sensitivity and reproducibility.

Chen *et al.* designed a magnetic relaxation switching biosensor based on a click chemistry-mediated sol–gel system for the detection of pathogenic bacteria *V. parahaemolyticus* (VP) by using a low-field nuclear magnetic resonance (LF NMR) detection approach. The biosensor involves hollow mesoporous silica microspheres (HMSMs) packed with saturated sodium ascorbate (SAA) and fully coated with an aptamer to resist SSA leakage, which may cause reduction of copper(ii) ions. In the presence of VP, the aptamer started binding with it, resulting in leakage of SSA. Copper(i)-catalysed 1,2,3-triazole formation between PEG-azide and PAA-alkyne developed a three-dimensional network structure. Several “free” waters turned into “bound” waters, which were characterised by a long transverse relaxation time (T2). Free water exhibits a long T2 characteristic, while bound water displays a high T2 value, as recorded through NMR response. Seawater samples and oyster samples were tested for VP detection with 96.6–106.4% and 97.6–105.3% recovery, respectively. The detection duration was 15 min, with an LOD of 5 CFU mL^−1^ as well as a detection range of 10–1.0 × 10^8^ CFU mL^−1^.^195^

Hu *et al.* designed a ratiometric electrochemical aptasensor for point-of-care testing of VP in shrimp aquaculture water. The sensing platform employed immobilisation of c-DNA-MB and an aptamer on an Au electrode (Au–S bonding). In the presence of VP, c-DNA was released from the electrode surface due to the stronger binding force of VP and the aptamer. Released c-DNA-MB resulted in a decrease in the signal. Subsequently, a nano-metal–organic framework@AMP-Fc signal probe was synthesised through a CuAAC reaction between ferrocene alkynyl and the azido-aptamer. The 1,2,3-triazole-assisted signal probe formed a sandwich complex with captured VP, and ferrocene signalling was obtained by the Au electrode. Shrimp aquaculture water samples were analysed, and good recoveries of 97.0% and 106% were obtained, with an LOD of 4 CFU mL^−1^ within 30 minutes.^196^

Wang and co-authors fabricated a portable bioluminescence aptasensor for the identification of live *S. typhimurium* within 20 min, with an LOD of 8 CFU mL^−1^. The biosensor principle combined a carboxyl-modified primer chain, a circular template and alkynyl-modified dUTP (5-ethynyl-dUTP), which underwent rolling circle amplification (RCA) and was conjugated to an aptamer through a 1,2,3-triazole linkage *via* CuAAC to achieve the desired hyperbranched aptamer probes (HAPs). The HAPs were further immobilized onto a Framework-8-NH_2_-coated glass sheet to make a stir bar. In the same way, several stir bars were synthesised, and an array of stir bars was synthesised to capture the bacteria. Captured bacteria were hydrolysed with lysozyme, and ATP was released from the live bacteria and detected with a bioluminescence sensor in 15 s. Many aquatic samples were analysed with recovery rates ranging between 90.9–113%, and the biosensor was also used to distinguish between live and dead bacteria. The approach holds promise for quality control and live food-borne pathogen detection in complex water samples.^197^

Wang *et al.* designed a dual-mode bioluminescence assay and microfluidic chip assay as a biosensor for the detection of pathogenic bacteria (*E. coli* and *H. paralvei*) in fish samples. The sensing process initiated from a reaction between alkyne-functionalized RCA chain-bonded MBs and an azide-modified phage (phage-E or phage-H). The generated 1,2,3-triazole linkage resulted in MPEP (MPEP-E and MPEP-H), which conjugated to different dsDNA signal tags to form a sandwich structure. Further, the sandwich structure was lysed by a lysosome, which released ATP that was quantified with an ATP bioluminescence meter. Under analysis, recovery rates were 93.7–109% and 90.0–106% for *E. coli* and *H. paralvei*, respectively.^198^

Liu and team designed a colourimeter sensor for detection of *S. aureus* in lake water and pork samples with an LOD of 2.4 CFU mL^−1^. The analytical method of biosensing involved an *S. aureus*-bonded aptamer of Fe_3_O_4_@ALP nanoparticles and magnetic separation of the aptamer–bacterium complex. Further ALP of the NPs hydrolyses the l-ascorbate-2-phosphate (AP) to form ascorbic acid, which reduced copper(ii) to copper(i) and catalysed the click reaction between N_3_-PEG-AuNPs and CH-PEG AuNPs. 1,2,3-Triazole aggregated the click AuNPs and amplified the colour signal from red to blue. Whereas iron NPs, which specifically bind to *S. aureus*, block the binding site for ALP. Reduced ALP exposure results in unreacted azide–alkyne AuNPs and no colour change. Recovery rates of the biosensors in pork and lake water samples ranged from 91.12% to 106.36%.^199^

Xu and co-authors fabricated a biosensor based on multivalent DNA walker amplification for the detection of food-borne pathogens (*V. parahaemolyticus*, *S. typhimurium,* and *S. aureus*) with LODs of 28, 10, and 9 CFU mL^−1^, respectively. The analytical method employed multivalent DNA walker amplification with a 4-mercaptobenzeneboronic acid-immobilized gold bar to conjugate with bacterial pathogens and a signal probe (modified with phenylboronic acid). Upon exposure to Zn^2+^ ions, capture probes cleaved the RNA site of the signal probe, leading to the formation of many DNA fragments. Therefore, DNA fragments were separated and detected by Microfluidic Chip (MC) technology. The signal probe was generated through a click reaction between PBA (alkynyl-modified) and the E17 (azido-modified) DNA strand to form the 1,2,3-triazole, whereas the capture probes were generated through a SPAAC reaction between DBCO-NHS (containing both alkynyl and ester groups) and the azido-modified DNAzyme strand and antibodies containing amino groups. The biosensor showed satisfactory detection results in different water samples and mixed spiked samples.^200^

#### Bacterial detection in clinical samples

The rapid and accurate bacterial detection in clinical samples for identifying infectious diseases is essential. Delayed diagnosis can cause extreme health burdens such as neurological diseases, skin and wound infections and urinary and reproductive tract infections, *etc.*^201,202^ To implement specific therapeutic measures and to improve patient recovery and reduce clinical complexity, rapid and accurate detection of pathogens in clinical samples is essential. This section of the review explores the rapid and accurate detection of bacteria in clinical samples by 1,2,3-triazole-aided DNA biosensors.

Xiong and co-authors designed a biosensing technique for the identification of *E. coli* in infected bloodstreams. The biosensing mechanism is based on the hydrolysis of 4-aminophenyl β-d-galactopyranoside to *para*-aminophenol with the aid of β-Gal, an enzyme collected from lysed *E. coli*. Further, *para*-aminophenol reduced copper(ii) to copper(i), which catalysed the click reaction between alkyl DNA and azide DNA and formed P1-DNA *via* a 1,2,3-triazole linkage. Subsequently, P1-DNA facilitated the hybridisation of H1 and H2 probes to generate azide-DNA-alkynyl-DNA-(H1–H2)_*n*_. The generated azide-DNA-alkynyl-DNA-(H1–H2)_*n*_ clustered the copper nanoparticles when ascorbic acid and copper(ii) ions were added, which resulted in a fluorescence signal. The biosensor exhibited 100% specificity and 83.3% sensitivity in urine and blood samples with an LOD of 0.003 CFU mL^−1^. Therefore, the biosensor has excellent potential for point-of-care testing within 3.5 hours.^203^

Li and co-authors synthesised a biosensor-based ATRP method, as discussed earlier. The biosensor was successful in bacterial detection, and drug resistance was analyzed by monitoring antibiotic metabolism, which helped to control the emergence of multidrug-resistant bacteria. As already discussed, in the ATRP technique, the mechanism of signal amplification was through polymer growth. The biosensor with the reducing property of *S. aureus* and *E. coli* converted copper(ii) to copper(i), which catalysed the radical production that enabled rapid drug analysis. This was confirmed by inhibiting the bacterial metabolism with antibiotics, which resulted in lower concentrations of copper(i) ions and reduced signal amplification. This biosensor was used for analysis of *S. aureus* and *E. coli* with LODs of 4 and 6 CFU mL^−1^, respectively.^131^

Chen and co-workers developed a biosensing approach for rapid and visual detection of urinary pathogens in real urine samples. The biosensor mechanism was based on magnetic Au@Fe_3_O_4_ particles functionalized with bifunctional DNA of bacteria or 18S ribosomal ribonucleic acid of fungi secreted from pathogens in urine samples. Exonuclease III (Exo-III) was triggered to cleave bfDNA while maintaining the target RNA when a blunt-ended DNA structure was created. CuO nanoparticles were assembled through DNA hybridization and liberated by the cleavage process. The click reaction was facilitated by the released CuO concatemers under acidic conditions after magnetic separation, which released copper(ii) ions and reduced them to copper(i). Gold nanoparticles were functionalized with azide- and alkyne-modified DNA and created a 1,2,3-triazole ring. The gold nanoparticle solution undergoes a noticeable colour shift from red to purple as a result of this aggregation, which may be measured *via* absorbance spectroscopy or seen with the unaided eye. The biosensor was utilised for UTI detection and bacterial strain detection, and antimicrobial susceptibility testing was achieved within 55, 55, and 145 min for 96 samples. The sensitivity and specificity of the biosensor were 99% and 100% for Gram-negative bacteria (G−) infection, 100% and 100% for Gram-positive bacteria (G+) infection, and 98% and 100% for AST, respectively (Fig. 22).^204^

**Fig. 22:**
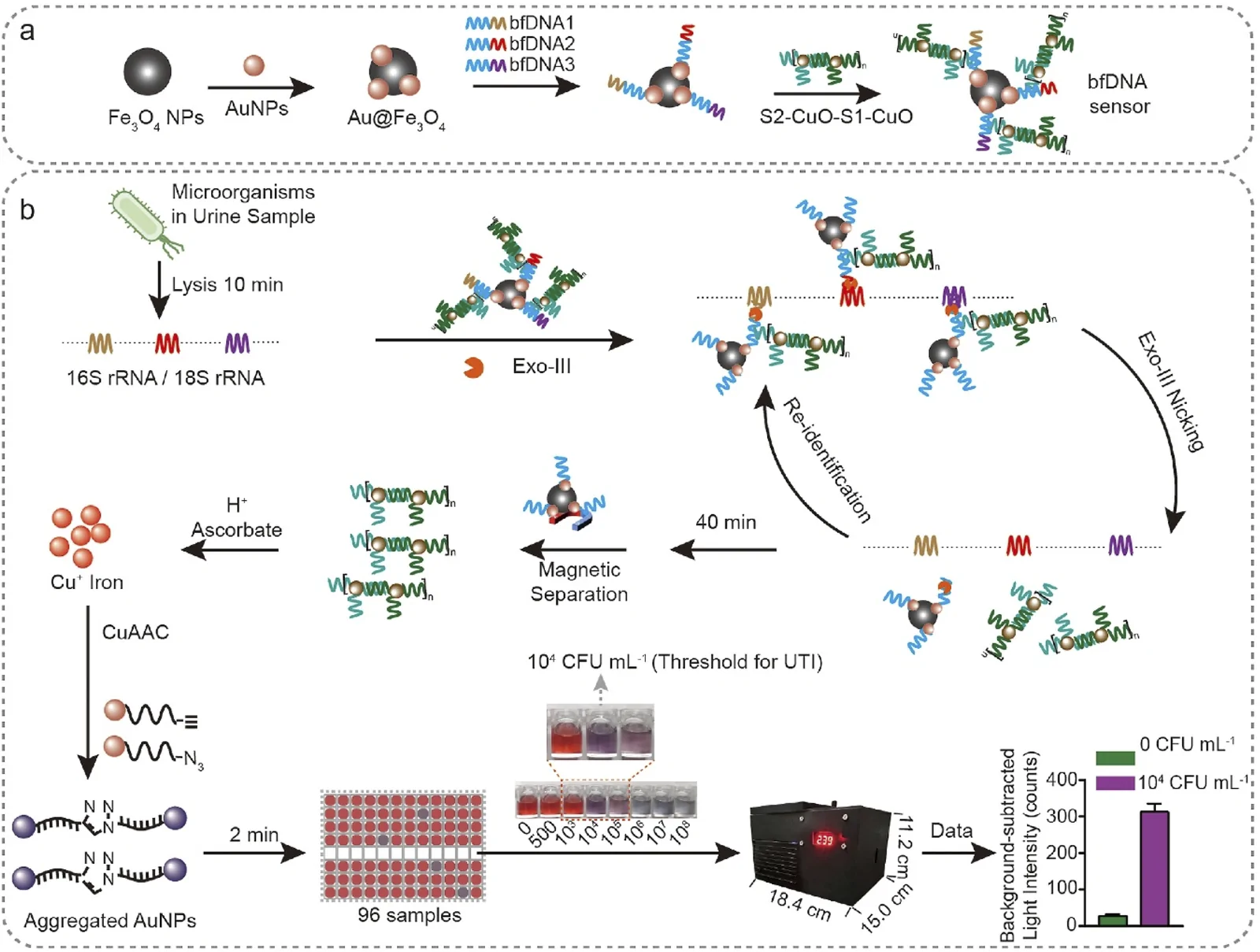
Principle of the bfDEC-AA method for detection of urinary pathogens^204^ (adapted/reproduced from ref. 204 with permission from the American Chemical Society, copyright 2024). (a) Preparation of the smart bfDNA sensor. (b) Principle of the bfDEC-AA method.

#### Virus detection

Viruses are small, non-cellular pathogens that pose serious threats to human health and impose a significant economic burden worldwide. Notable recent examples include severe acute respiratory syndrome coronavirus 2 (SARS-CoV-2) and Ebola virus. Similarly, plant viruses are highly detrimental to agriculture and cause billions of dollars in global economic losses each year.^205^ To control and manage the transmission of viral diseases and to save millions of dollars in healthcare and agricultural costs, it is essential to identify causative viruses rapidly, sensitively, and accurately.^206^ Herein, the review presents CuAAC-based biosensors with sensitive and real-time detection capabilities for viruses such as SARS-CoV-2 and tobacco mosaic virus.

#### SARS-CoV-2 detection

Zhang *et al.* fabricated a biosensor relying on nanocomposites with aggregation-induced emission luminogen-labeled DNA probes on graphene oxide nanosheets for severe acute respiratory syndrome coronavirus 2 (SARS-CoV-2) detection. The detection technique was facilitated by a CuAAC click reaction between azide-functionalized tetraphenylethene (TPE) and an alkyne-modified single-strand DNA primer (alkyne-ssDNA), which formed TPE-DNA (aggregation-induced emission (AIE) fluorogen (AIEgen)) *via* a 1,2,3-triazole linkage. TPE-DNA immobilized on a GO nanocomposite (AIEgen@GO) hybridized to form DNA/RNA duplex-TPE molecules on exposure to the target SARS-CoV-2 viral sequence. Subsequently, the formed TPE molecule detached from the GO, triggering the first stage of fluorescence recovery from “OFF” to “WEAK”. Further mass changes from ssDNA to dsDNA led to an enhanced fluorescence signal from “WEAK” to “STRONG”. The authors tested the biosensor feasibility on synthetic SARS-CoV-2 only; no real-life samples were analysed for detection.^207^

Ji Lu *et al.* engineered an electrochemical biosensor for SARS-CoV-2 detection *via* a 1,2,3-triazole-based ATRP technique. The biosensor employed Cp1-Cu_3_(PO_4_)_2_HNFs nanoflowers linked to streptavidin-modified MBs conjugated to biotin-modified Cp2. This linkage captures the target and serves as a bridge to complete the linkage. In the presence of AA, copper(ii) was reduced to copper(i) to facilitate the click reaction between the gold electrode-modified azide and sulfhydryl to form a 1,2,3-triazole linkage, which was further used in the ATRP platform, as already discussed. The biosensor performance was analysed with saliva samples and showed recovery rates of 96.47–101.04%.^208^

#### Small molecules detection in clinical samples

Many small molecules, including toxins, pharmaceuticals, illicit drugs, and endogenous compounds, are harmful to living organisms.^210^ Small molecules are generally detected by HPLC, capillary electrophoresis (CE), and enzyme-linked immunosorbent assay (ELISA) due to their small size. However, these excellent methods require high-cost instrumentation and are time-consuming.^211^ 1,2,3-Triazole-assisted DNA biosensors can be used to detect small molecules with high efficacy and at a lower cost. The quantitative analytical performance of representative click-chemistry-based DNA biosensors for small-molecule detection is compared in Table 3.

**Table 3:** Analytical performance of click-chemistry-based DNA biosensors for the detection of smaller molecules

Target	Detection method	ROD (µM)	Detection limit (µM)	Real-life samples	Recovery rate	Ref.
Guanine and adenine	Electrochemical	4 to 20	1.07 and 2.91	Calf thymus		143
Ascorbic acid	Electrochemical	0.1 to 1000	4.5 × 10^−2^	Human serum samples	96.48% to 104.69%	212
Quinine	Electrochemical	0.05 to 10	25 × 10^−2^	Tonic water	95.7% to 103.74%	213
Glutathione	ECL	5 to 200	0.90	Human serum samples	98.5–101.9%	151
Methamphetamine	Electrochemical	0.1 to 1 × 10^−6^	17 × 10^−9^	Human serum and urine	93.3% to 98.22%	134
Digoxin therapeutic	Electrochemical	1.0 × 10^−6^ to 4 × 10^−5^	5.9 × 10^−7^	Real blood samples		135
Bisphenol A	Electrochemical	1 × 10^−8^ to 0.1	5.9 × 10^−11^	Pure water samples	95.23% to 98.40%	215
Biomolecules	Electrochemical	1.0 × 10^−17^ to 1.0 × 10^−5^	1.4 × 10^−12^	Human blood serum		214

Chen *et al.* constructed an electrochemiluminescence biosensor for ascorbic acid detection, with an ROD ranging between 0.1 and 1000 µM. In this biosensing approach, the key process involves ruthenium(ii) chloride hexahydrate-doped SiO_2_ nanoparticles (Ru@SiO_2_ NPs) immobilized over an electrode surface with the aid of chitosan. Upon addition of the target AA, copper(ii) is reduced to copper(i), which catalyses the CuAAC reaction between ssDNA modified with alkynyl and azido groups. The 1,2,3-triazole linkage activated the gene circuit, which facilitated the formation of an RNA output strand in the presence of T7 RNA polymerase. Subsequently, the RNA output strand conjugates with ferrocene-DNA to form a DNA–RNA duplex. DSN cleaved the DNA portion of the duplex, leading to the detachment of Fc-DNA from Ru@SiO_2_ NPs and re-establishing an ECL signal. The biosensor yielded a recovery rate of 96.48% to 104.69% in serum samples, demonstrating its potential for clinical application. This approach broadens the range of biosensing applications to a cell-free RNA transcription system.^212^

Luo and co-workers developed an electrochemical detection approach for quinine, based on a small-molecule-templated split aptamer click ligation reaction (SMT-SpA-CLR) in tonic water. The detection process relies on QN1 modified with an azide and a thiol group, whereas QN2 consists of an alkyne moiety and methylene blue. In the presence of the small molecule quinine, QN1 and QN2 formed a weakly bound ternary complex. Subsequently, QN1 and QN2 triggered a click reaction in the presence of copper(i) to give the 1,2,3-triazole linkage. Further ligation over gold electrode associated with electron transfer facilitated the detection of methylene blue between gold electrodes, in the form of a signal with recovery rate of 95.7% to 103.74% and LOD of 25 × 10^−2^ µM (Fig. 23).^213^

**Fig. 23:**
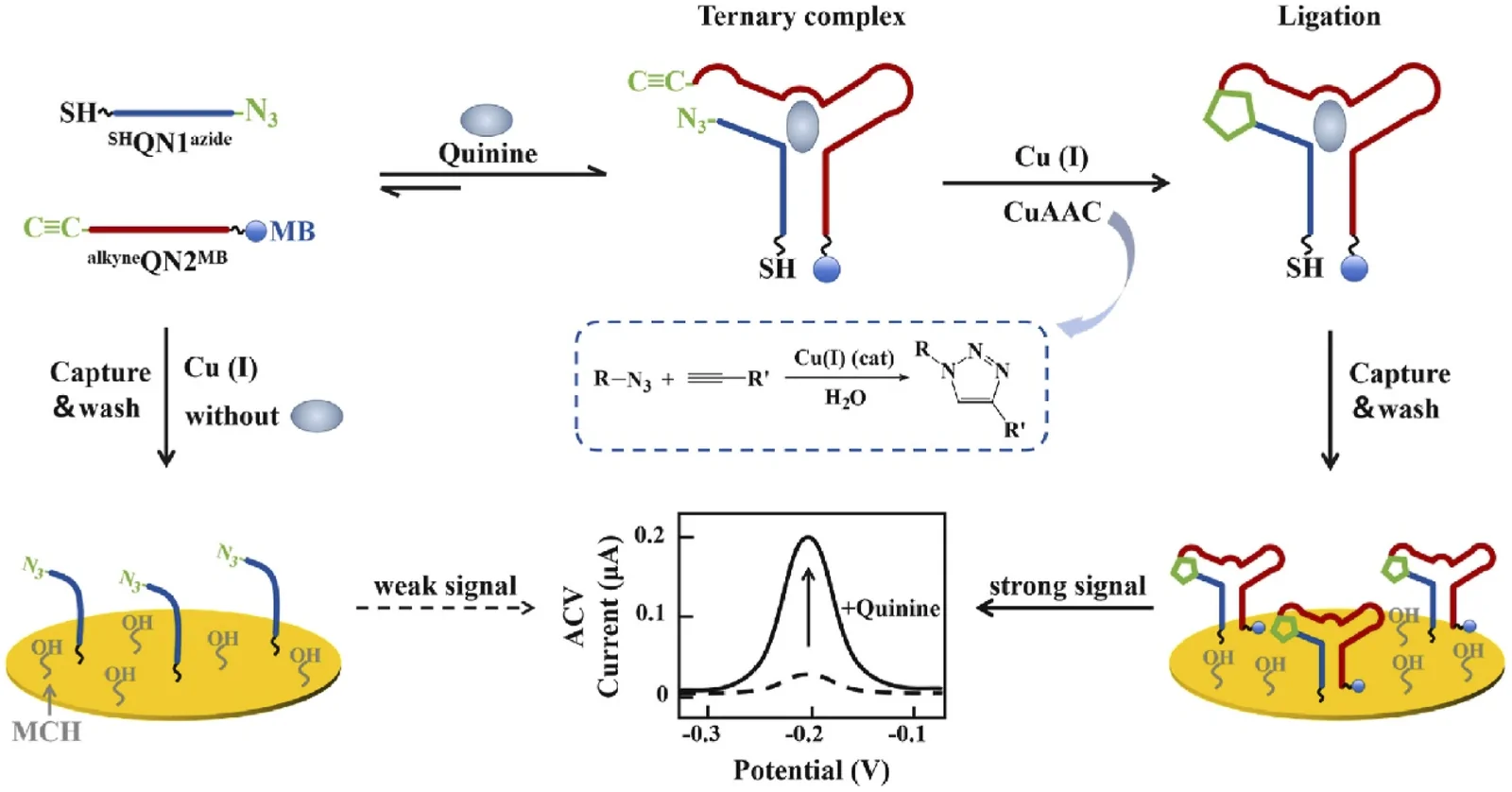
Schematic of the electrochemical sensing systems using small-molecule-templated split aptamer click ligation (SMT-SpA-CLR)^213^ (adapted/reproduced from ref. 213 with permission from Elsevier, copyright 2025).

Zhang *et al.* used a click-assisted ATRP platform for digoxin therapeutic monitoring in clinical samples. The biosensor successfully detected digoxin in real blood samples of healthy individuals and of patients receiving digoxin therapy. The biosensor exhibited high sensitivity and low cost, but the response time was long (4–5 h) and only a single analyte could be processed at a time. However, these biosensors outperformed ELISA, FPIA and LC-MS/MS, exhibiting high sensitivity and low cost with an LOD of 5.9 × 10^−7^ µM.^135^

Radfar and co-authors worked on an ATRP-based biosensing platform for biomolecules that involved a DNA walker. The sensing was initiated through DNA walker dsDNA and H1 probe immobilized on the surface of an Fe_3_O_4_ NP-functionalized GCE. After the addition of prostate-specific antigen (PSA), DW was released, and the catalytic assembly between H1 and H2 was triggered. The azide moiety of the H1 probe underwent a click reaction with PBIB and formed 1,2,3-triazole, which was subsequently linked to the signal probe.^214^

#### Food and environmental identification

Global population growth and technological advancements have led to a significant increase in environmental degradation over time and have placed more strain on the world's food system to meet the rising demand for wholesome, safe products. Food contamination is a serious issue that can affect human health and requires close monitoring of heavy metal ions, pesticide residues, foodborne pathogens, mycotoxins, and medicines, all of which seriously threaten human health through contaminated food and environmental resources, resulting in enormous financial losses.^22,24,216^ This section of the review explores the use of click-assisted DNA biosensors for food and environmental safety.

#### Heavy metal ions detection

Heavy metal contamination is harmful to both the environment and human health and is a major global concern. Heavy metals ingested in excess of permitted levels can present major health hazards. Heavy metals can pose serious health risks when consumed in excess of regulatory limits. It is crucial to develop effective and affordable techniques for identifying these heavy metals.^217–219^ Click-assisted biosensors provide detection in real-life samples with high sensitivity and selectivity. Table 4 compares the performance of representative click-chemistry-based DNA biosensors for the quantitative analysis of heavy metals.

**Table 4:** Quantitative analytical performance of click-chemistry-based DNA biosensors for the detection of heavy metals

Target	Detection method	ROD (µM)	LOD (µM)	Real-life samples	Recovery rate	Ref.
Lead(ii) ions	Fluorescence	5 × 10^−2^ to 3.5 × 10^−2^	1.245 × 10^−2^	Swimming crab	81.16% to 106.7%	103
Copper(ii) ions	Electrochemical	1.0 × 10^−6^ to 5 × 10^5^	3.3 × 10^−7^	Tap water	98.8% to 105%	150
Total copper	Electrochemical	0.1 × 10^−4^ to 1	3.5 × 10^−3^ and 8.0 × 10^−4^	Mining industry sample		121
Lead(ii) ions and magnesium(ii) ions	Wavelength-resolved technology	1 × 10^−3^ to 1.5 × 10^−4^	3.3 × 10^−4^			221
Copper(ii) ions	Calorimetric	50 × 10^−2^ to 0.5	2.68 × 10^−4^	Mineral water	90.8% and 99.8%	119
Copper(ii) ions	Electrochemical	1.0 × 10^−12^ to 10	3.8 × 10^−13^	Tap water samples		222
Copper(ii) ions	Electrochemical	1 × 10^−4^ to 1	2.81 × 10^−5^	Grape juice and milk samples	92.96% to 102.02% and 103.61% to 110.75%	107
Copper(ii) ions	Fluorescence	0.3 to 150	0.1	Natural water	89–102%	124
Cadmium(ii) ions	Fluorescence	1 × 10^−5^ to 3 × 10^−3^	3.6 × 10^−6^	Swimming crabs	85.99% to 92.27%	220
Copper(ii) ions	Electrochemiluminescence	1 × 10^−7^ to 0.1	3.3 × 10^−8^	Human hair solutions		46
Copper(ii) ions	Calorimetric	5 × 10^−3^ to 0.5	2 × 10^−3^	Human serum samples		61
Copper(ii) ions	Current signal	1.0 × 10^−4^ to 5 µM	6.7 × 10^−5^	Human serum and tap water samples	89.5% to 105.3%	223
Copper(ii) ions	Fluorescence and calorimetric	0.2 to 4.0 and 0.02 to 1.6	6.85 × 10^−2^ and 7.04 × 10^−2^	Human blood samples	91.7% to 107.1%	58
Copper(ii) ions	Electronic balance	2.0 × 10^6^ to 2 × 10^8^	8.3 × 10^−2^	10 Chinese herbal medicine		224
Copper(ii) ions	Fluorescence	0.01 to 0.1	0.01	Diluted human urine samples	73.8% to 86.23%	225
Copper(ii) ions	Fluorescence	0.1 to 1.0 × 10^−7^	2.6 × 10^−11^	Copper(ii) ion imaging		149

Peng and team synthesised a fluorescent probe-based biosensor for the identification of cadmium(ii) in aquatic products with a LOD of 3.6 × 10^−6^ µM. The recovery was 85.99% to 92.27% in swimming crab samples. The working mechanism of the biosensor was initiated through aptamer-peptide conjugation *via* a click reaction between the alkyne-modified peptide and azide-modified dsDNA sequence. The generated APC hybridised with the primer, whereas Cd(ii) ions bonded to APC, triggering the release of the primer. Subsequently, the released primer conjugated to the DNA template and formed a loop with T4 DNA ligase, which resulted in rolling-circle amplification (RCA). The RCA led to a long single-stranded DNA sequence, exhibiting many repeated G-quadruplex sequences, and, in the presence of hemin, activated the G-quadruplex to form a DNAase complex that performed oxidative addition to *o*-phenylenediamine (OPD) to form 2,3-diaminophenazine (DAP) in the presence of hydrogen peroxide. A PET (photoinduced electron transfer) occurred between DAP and graphene quantum dots that led to an increase in DAP fluorescence intensity and a decrease in GQD's fluorescence intensity (Fig. 24).^220^

**Fig. 24:**
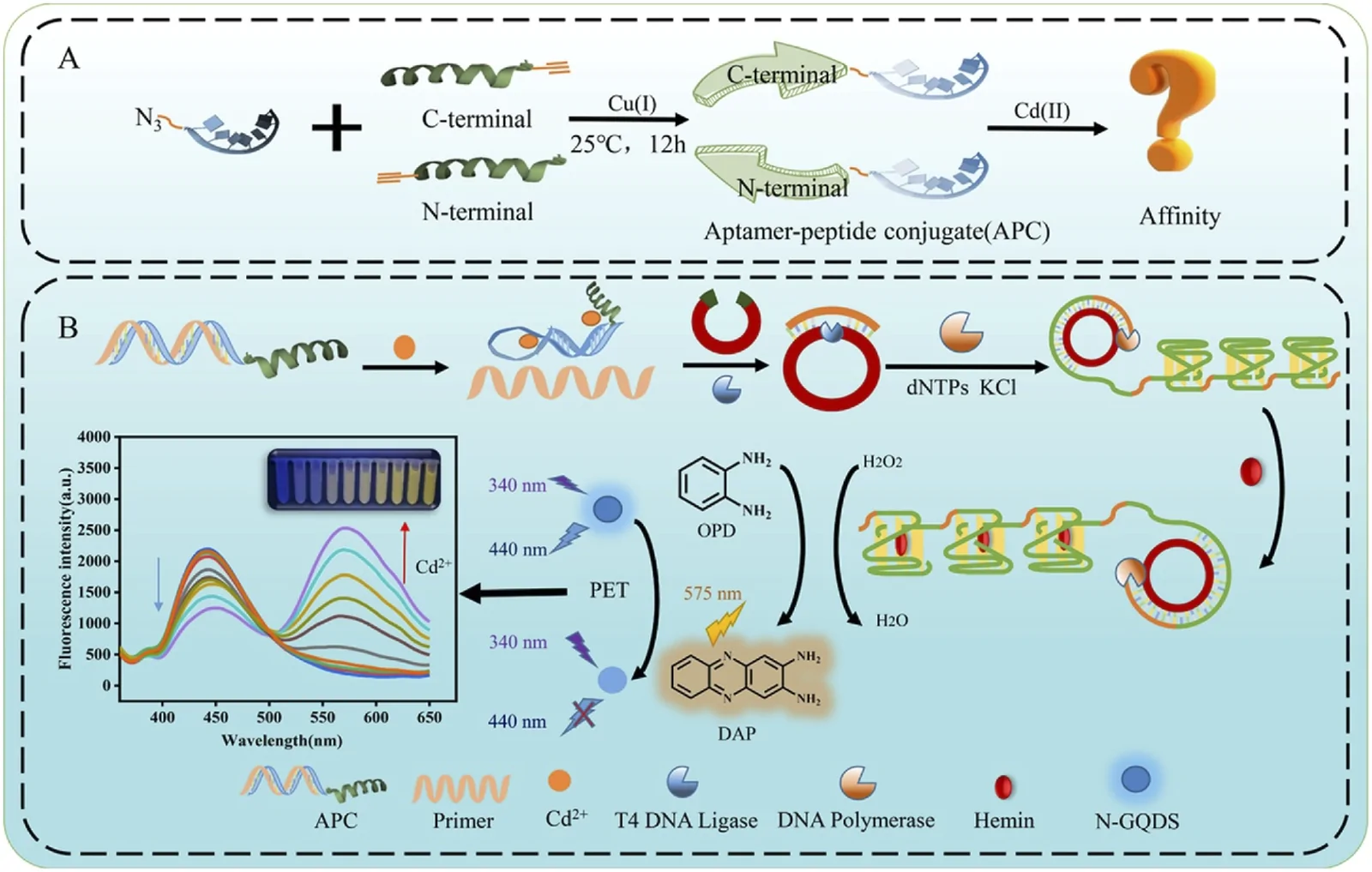
(A) Schematic of the APC design with distinct peptide orientations (N-terminal and C-terminal) produced using the click chemical approach and (B) an APC-based biosensor detecting Cd^2+^*via* RCA-coupled ratio fluorescence^220^ (adapted/reproduced from ref. 220 with permission from the American Chemical Society, copyright 2024).

Deng *et al.* engineered a photoelectrochemical (PEC) biosensor with DNAzyme-assisted cleavage and recycling amplification for metal ion detection. The sensor technique involves azido-S1 and azido-S2 cleaved by lead(ii) ions (target 1) and magnesium(ii) ions (target 2) in the ion-dependent DNAzymes immobilized on Au@Fe_3_O_4_. The electrode-functionalized DNA modified with alkyne and hexanethiol underwent CuAAC with azido-S1 and azido-S2, and the 1,2,3-triazole formed captures Pb(ii) ions and Mg(ii) ions, with signal outputs recorded at 365 and 623 nm. The biosensor demonstrated potential for use in environmental monitoring, food safety, and disease diagnosis, with a limit of detection of 3.3 × 10^−4^ µM and a detection range of 1 × 10^−3^ to 1.5 × 10^−4^ µM.^221^

Lv *et al.* designed a sensing technology for copper(ii) detection based on horseradish peroxidase-functionalized gold nanoparticles. The sensing technology involved a HRP-AuNP DNA signal probe synthesised through horseradish peroxidase and a thiolated alkynyl-DNA linker and Au-NP. Further, copper(ii) was reduced to copper(i) *via* sodium ascorbate to catalyse the CuAAC. A click reaction between the thiolated azido-DNA functionalized electrode and the HRP-AuNP alkynyl DNA signal probe resulted in 1,2,3-triazole. However, HRP catalysed the degradation of hydrogen peroxide to amplify the signal. The feasibility of the biosensor was demonstrated in tap water samples, with an LOD of 3.8 × 10^−13^ µM.^222^

#### Copper ion detection in clinical samples

The excessive direct or indirect use and consumption of heavy metals pose significant health hazards. Abnormal levels of copper(ii) ions are linked to conditions such as Wilson's disease and Menkes syndrome, and they can also affect diseases such as Alzheimer's and Parkinson's disease. The accurate detection of copper metal in clinical samples, especially body fluids including serum, urine, saliva, sweat, and tears, is important for diagnosis.^28^ Herein, the detection of copper(ii) ions through 1,2,3-triazole-aided DNA biosensors in clinical samples is discussed, demonstrating the potential of sensors in the medical health sector.

Liu *et al.* designed a biosensing strategy based on a nanopore sensing technique for copper(ii) ion detection, involving 1,2,3-triazole formation between alkyne-ssDNA and azide-ssDNA in the presence of the target copper(ii) and sodium ascorbate. A forked DNA probe was formed, which conjugated with α-hemolysin, leading to current signals. The feasibility of the biosensor was assessed in human serum and tap water samples, yielding recovery rates of 89.5–105.3% and 90.2–103.8%, respectively.^223^

Wu *et al.* designed a technique for copper(ii) ion identification in herbal medicine through click chemistry. The analytical method utilizes azide-DNA functionalised magnetic beads of streptavidin (MB-DNA) and alkynyl–DNA-modified streptavidin (Pt NPs-DNA) coated platinum nanoparticles, which underwent CuAAC through copper(ii) reduced *via* sodium ascorbate to give 1,2,3-triazole linkage formation leading to MB-Pt NPs. Subsequently, magnetic separation was used to separate the MB-Pt NPs, and the biosensor potential for real-life analysis was quantified in 10 Chinese herbal medicines. The results were satisfactory, exhibiting a detection limit of 8.3 × 10^−2^ µM under 30 min of collection time.^224^

Ge and co-authors fabricated a sensing platform by using graphdiyne nanosheets for copper(ii) detection. The sensing method involved click reaction *via* butadiyne groups of GDY NSs and N_3_ groups of N_3_-dsDNA-6-carboxyfluorescein (N_3_-dsDNA-FAM) upon exposure to the target copper(ii) and ascorbic acid. The generated 1,2,3-triazole linkage enabled FRET from FAM to GDY NSs due to the nearby positioning of FAM and GDY NSs, which led to the fluorescence quenching of FAM.^225^

#### Detection of toxins

In daily life, individuals are unintentionally exposed to hazardous toxins, either anthropogenic or natural.^226^ These mycotoxins cause harm to living organisms; for example, ochratoxin A is immunotoxic to various animals and causes kidney failure and liver problems.^45^ The presence of different poisons in food and water is a serious concern that requires accurate food and environmental monitoring. For the purpose of analyzing and quantifying hazardous substances, biosensors must be developed quickly. Innovative DNA-based sensors can improve sensitivity while saving money and time.^24^ In recent discoveries, 1,2,3-triazole-click-assisted DNA biosensors have shown impressive and excellent results in real-time detection. Representative quantitative analytical performance of click-chemistry-based DNA biosensors for toxin detection is compared in Table 5.

**Table 5:** Quantitative analytical performance of click-chemistry-based DNA biosensors for the detection of toxins

Target	Detection method	ROD	LOD	Real-life samples	Recovery rate	Ref.
Ampicillin	Fluorescence	10^−7^ to 10^−2^ mg mL^−1^	80 pg mL^−1^	Milk and river water samples	95% to 107%	59
Ampicillin	Electrochemical	1 nM to 1 mM	1.36 nM	Tap water, milk, and saliva	104%, 88.2%, 90.4%	231
AFB_1_ detection	Electrochemical	2 fg mL^−1^ to 2 ng mL^−1^	0.804 fg mL^−1^	Malt and corn	94.73% to 101.46%	64
Ochratoxin A	Personal glucose meter	0.2 to 5.0 ng mL^−1^	72 pg mL^−1^	Feed samples	86% to 103.5%	50
Food allergenic protein	Fluorescence	4 × 10^−8^ to 1 × 10^−6^ g mL^−1^	1.5 × 10^−8^ g mL^−1^	Different milk samples		227
Aflatoxin B_1_	Calorimetric	100 pg mL^−1^ to 50 ng mL^−1^	26.23 pg mL^−1^	Peanut and maize samples	81.63% to 112.21% for peanut samples and 80.93% to 113.95% for maize samples	104
Okadaic acid	SERS	1.0 to 300.0 nmol L^−1^	0.2 nmol L^−1^	Mussel, oyster, scallop and mussel meat samples	93.5% to 106%	229
Organophosphorus pesticides	Fluorescence	2 to 60 ng mL^−1^	1.4 ng mL^−1^	River water samples	99.73% to 102.36%	230
Aflatoxin B_1_	Fluorescence	1 to 10^5^ pg mL^−1^	0.48 pg mL^−1^	Corn and peanut	85.90% to 110.63% and 82.47% to 113.70%	228
Glyphosate	Calorimetric and fluorescence	0.5 to 15 µg mL^−1^ and 0.5 to 7 µg mL^−1^	0.19 µg mL^−1^ and 0.15 µg mL^−1^	Tap water and soyabean	90.89% to 105.06%	123
Diclofenac	Electrochemical	17.95				47
Diclofenac	Electrochemical	5 × 10^−3^ to 0.1	3.1 × 10^−3^			48

Zhang *et al.* fabricated a sensitive fluorescent clickase-linked immunosorbent assay (FCLISA) platform for food allergenic protein identification in raw cow milk, ultra-heat-treated (UHT) cow milk, partially hydrolyzed infant formula, and extensively hydrolysed formula. The biosensor strategy involves a hairpin DNA functionalized with a quencher and a fluorophore, which underwent a click reaction with Oligo-A and Oligo-B labelled with 5′-alkyne and 3′-azide groups. Cu_2_O nanocubes trigger a CuAAC reaction, leading to the formation of an Oligo-MB hairpin structure *via* 1,2,3-triazole linkage, which acted as a fluorescence switch. The fluorescent clickase-linked immunosorbent assay (FCLISA) then takes place, linking the clickase to the detection antibody (Ab_2_) of the allergenic protein casein. Protein casein influenced the Oligo-MB hairpin structure, and the fluorescence signal was amplified and observed with a fluorescence spectrometer. The biosensing platform exhibited a low LOD of 1.5 × 10^8^ g mL^−1^ and a ROD of 1 × 10^8^ to 1 × 10^6^ g mL^−1^, validating its suitability for use in food and environmental monitoring.^227^

Hong and co-authors developed an immunosensor for Aflatoxin B_1_ detection with an LOD of 0.48 pg mL^−1^. The biosensor was based on the immobilization of antigen (BSA-AFB_1_) over Cu-MOF, which acted as a reservoir of copper(ii) ions. In the presence of SA, a click reaction was facilitated *via* reduced copper(ii) to form a 1,2,3-triazole ring between the receptor-modified complementary DNA hexyne and fluorescent donor-modified DNA azide. The click reaction led to fluorescence signals and confirmed the presence of Aflatoxin B_1_ in real corn and peanut samples with recovery rates of 85.90% to 110.63% and 82.47% to 113.70%, respectively.^228^

Wang and co-workers designed a DNA hydrogel sensor based on the RCA and click conjugation reaction with target-responsive DNAzyme signal amplification. The sensor exhibited a low LOD of 0.2 nmol L^−1^ and a detection time of 30 min, with a ROD of 1.0–300.0 nmol L^−1^ for monitoring okadaic acid *via* SERS (surface-enhanced Raman scattering) detection method. In this biosensing approach, the key process involves a 5-azido-PEG4-dCTP-functionalized RCA strand synthesised by a rolling circle amplification approach with a circular template strand and an azide group. The generated azido-RCA product underwent click conjugation with ethynyl group-modified cross-linked DNA strands *via* 1,2,3-triazole to form DNA hydrogels. In the presence of okadaic acid, the DNAzyme was freed from the DNA hydrogel and cut the specific sequence in the substrate DNA strand consisting of ribonucleotide adenosine. As a result of cleave of ribonucleotide adenosine, the DNA hydrogels lysed and the Raman signal was amplified. Detection recovery rates varied between 93.5–106% in mussel, oyster, scallop and mussel meat samples. The above characteristics exhibited by the biosensor validate the use of the biosensing platform for food and environmental monitoring.^229^

A sensitive fluorescence assay was developed by Huang and co-workers for organophosphorus pesticides in river water samples. The biosensing involved acetylcholinesterase (AChE) facilitated hydrolysis of acetylthiocholine (ATCh), forming thiocholine (TCh) in the absence of omethoate. In the presence of omethoate, ATCh was unable to bind to Cu(ii), allowing sodium ascorbate to reduce it to Cu(i). The resulting Cu(i) facilitated the click reaction of DNA probes between P1 and P2, modified with an alkyne group and an azide group and FAM on their ends. Subsequently, the BHQ1-labelled probe P3 hybridized with P1 and displaced P2. As P1 and P3 linked, P2 is drawn closer to P3 and hybridized with it to form a P1P2P3 complex. The quenching group (BHQ1) of P3 was in proximity to the fluorescent group (FAM) of P2, leading to a decrease in fluorescence signal due to fluorescence resonance energy transfer. The analytical performance of the biosensor was analysed using river water samples and gave recoveries of 102.3–99.7% with a limit of detection of 1.4 ng mL^−1^.^230^

Jose M. R. Flauzino and co-workers fabricated an aptasensor based on CuAAC for the detection of ampicillin in real samples, exhibiting a limit of detection of 1.36 nM and a shelf life of 4 weeks. In this biosensor, the carboxylic acid groups of graphene acid reacted with the primary amine groups of propargylamine to generate an alkyne-terminated graphene derivative. This functionalized surface then participated in a click reaction with an azide-modified DNA aptamer, leading to the formation of a five-membered 1,2,3-triazole ring. Ampicillin was successfully detected in PBS (phosphate-buffered saline) and tap water, with the latter showing a recovery of 104%. In contrast, saliva and milk yielded recovery rates of 90% and 88%, respectively. These results highlighted the potential of this biosensor for environmental monitoring, food safety analysis, and medical diagnostics. However, its shelf life of 4 weeks may be insufficient for long-term or large-scale applications.^231^

#### Impact of CuAAC on DNA-based biosensor performance

CuAAC chemistry contributes to DNA-biosensing through various mechanisms as discussed in this review. The formation of stable and bioorthogonal 1,2,3-triazole provides site-specific functionalisation of DNA probes, signal probes, nanomaterials and electrode surfaces without any interference with biological activity. Due to the reliable, strong and simple attachment of two moieties, CuAAC results in enhanced stability of the sensor, stronger probe linkage and better reproducibility compared to other immobilization techniques. Moreover, using these linkage strategies, the click reaction has been utilized with other techniques, such as ATRP, dual-end DNA-ligation, DNA-quadruplex, DNAzyme-catalysed sensing, and CRISPR/Cas12a methods. With CuAAC, these techniques emerged to facilitate effective target-triggered amplification, signal transduction and electron-transfer processes.

CuAAC aids in efficient signal transduction and the formation of advanced DNA structures (DNA walkers, sandwich structures, G-quadruplex, *etc.*) to intervene efficiently even in complex sample matrices. Numerous click-assisted biosensors have successfully achieved a limit of detection ranging from micromolar to femtomolar, while performing excellently in complex matrices, such as food, water, blood, and human serum samples. Subsequently, click-assisted signal probe polymerization, DNAzyme-catalysed, and G-quadruplex-based biosensors resulted in improved signal amplification and creation, which enhances analytical responses.

Similarly, another advantage of 1,2,3-triazole in biosensing is related to the diverse signal amplification platforms that are CRISPR-based, fluorescent, colorimetric, magnetic, electrochemical, and electrochemiluminescent. This versatile characteristic of CuAAC allows scientists to construct high-performing biosensing systems for many target analytes, such as nucleic acids, pathogens, toxins, metal ions and proteins. Together, these characteristics position CuAAC as an effective molecular instrument for enhancing the sensitivity, selectivity, stability, reproducibility, and practical use of next-generation DNA biosensors.

## Conclusion and future prospects

To summarise, CuAAC-assisted 1,2,3-triazole is an excellent tool for the fabrication of DNA-based biosensors by providing a selective and effective target for a wide range of analytes, including heavy metal ions, toxins, miRNAs, DNAs, pathogens, and biomolecules. There are numerous techniques, such as signal amplification strategies (DNAzyme CLICK-17/CLICK-T, dual-strand end linking, G-quadruplex formation, signal probe attachment, CRISPR, ATRP, DNA walker, nanomaterials), that significantly enhance the sensitivity of biosensors, enabling real-sample analysis with ultra-low detection limits. The major strengths of biosensors stem from multiple detection methods, such as fluorescence, ECL, electrochemical, and colourimetric, as well as high specificity and versatility. Their cost-effectiveness and modifiability are driving their use as replacements for conventional methods.

Despite these strengths, challenges remain unaddressed. Copper(i) catalyst involvement in the CuAAC reaction causes concerns regarding cytotoxicity and limits the applicability of the biosensor for *in vivo* samples. Although this has been addressed to some extent by the use of SPAAC techniques, its wide application is still awaited. Additionally, long response times and issues related to stability and reproducibility affect the widespread use of CuAAC-based DNA biosensors for practical implementation. Future research should focus on copper-free click chemistry methods, such as strain-promoted azide–alkyne cycloaddition, thiol–ene and IEDDA, Staudinger ligation and photo-click reactions, to improve biocompatibility. The need to merge these biosensor technologies into point-of-care devices is being aided by advanced nucleic acid engineering techniques (*e.g.*, CRISPR) and advanced materials, such as graphdiyne, MXene, MOFs, and AuNPs. Furthermore, coupling these sensing methodologies with AI-driven machine learning, signal processing algorithms and data analysis systems will improve accuracy and pattern recognition. The advanced modification of click-chemistry-assisted DNA biosensors for real-time sensing will enhance their applicability in clinical diagnostics, environmental monitoring, and food safety.

In a nutshell, click chemistry has immense potential for DNA-based biosensors that could revolutionize next-generation diagnostic technologies and on-site detection systems.

## Conflicts of interest

There are no conflicts of interest to declare.

## Abbreviations

AA: Ascorbic acid
ABTS: (2,2′)-Azino-bis(3-ethylbenzothiazoline-6-sulfonic acid)
AFB_1_: Aflatoxin B_1_
AHC: 3-Azido-7-hydroxycoumarin
ALP: Alkaline phosphatase
ATRP: Atom transfer radical polymerisation
AuNPs: Gold nanoparticles
BOL: 3-Butyn-1-ol
Cdna: Capture deoxyribonucleic acid
CFU: Colony-forming unit
CHA: Catalytic hairpin self-assembly
CRISPR: Clustered regularly interspaced short palindromic repeats
CuAAC: Copper(i)-catalysed azide–alkyne cycloaddition
Dnazyme: Deoxyribozyme
DSN: Duplex-specific nuclease
ECL: Electrochemiluminescence
ELISA: Enzyme-linked immunosorbent assay
Fc: Ferrocene
FMMA: Ferrocenylmethyl methacrylate
GCE: Glassy carbon electrode
HP1: Hairpin probe 1
HP2: Hairpin probe 2
LOD: Limit of detection
MB: Methylene blue
Mbs: Magnetic beads
MPBA: 4-Mercaptophenylboronic acid
MNP: Magnetic nanoparticles
Mrsa: Methicillin-resistant *S. aureus*
Mtase: Methyltransferase
NMR: Nuclear magnetic resonance
OTA: Ochratoxin A
PAP: P-Aminophenol
PBIB: Propargyl 2-bromoisobutyrate
PDA: Polydopamine
PEG: Polyethylene glycol
PCR: Polymerase chain reaction
Ppi: Inorganic pyrophosphate
RAFT: Reversible addition fragmentation chain transfer
RCA: Rolling circle amplification
ROD: Range of detection
SARS-Cov-2: Severe acute respiratory syndrome coronavirus 2
SERS: Surface-enhanced Raman spectroscopy
*S. typhimurium*
: *Salmonella enterica* serovar *typhimurium*
SWV: Square wave volumetry
TMB: 3,3′,5,5′-Tetramethylbenzidine
Tht: Thioflavin T
*V. parahaemolyticus*
: *Vibrio parahaemolyticus*
VEGF: Vascular endothelial growth factor

## Data Availability

No primary research results, software or code have been included, and no new data were generated or analysed as part of this review.
